# Confined Spaces in [*n*]Cyclo‐2,7‐pyrenylenes

**DOI:** 10.1002/anie.202102809

**Published:** 2021-05-24

**Authors:** Niklas Grabicki, Khoa T. D. Nguyen, Steffen Weidner, Oliver Dumele

**Affiliations:** ^1^ Department of Chemistry Humboldt Universität zu Berlin Brook-Taylor-Strasse 2 12489 Berlin Germany; ^2^ Bundesanstalt für Materialprüfung Federal Institute for Material Research and Testing Richard-Willstätter-Strasse 11 12489 Berlin Germany

**Keywords:** cycloparaphenylenes, host–guest systems, macrocycles, molecular recognition, supramolecular chemistry

## Abstract

A set of strained aromatic macrocycles based on [n]cyclo‐2,7‐(4,5,9,10‐tetrahydro)pyrenylenes is presented with size‐dependent photophysical properties. The K‐region of pyrene was functionalized with ethylene glycol groups to decorate the outer rim and thereby confine the space inside the macrocycle. This confined space is especially pronounced for n=5, which leads to an internal binding of up to 8.0×10^4^ m ^−1^ between the ether‐decorated [5]cyclo‐2,7‐pyrenylene and shape‐complementary crown ether–cation complexes. Both the ether‐decorated [n]cyclo‐pyrenylenes as well as one of their host–guest complexes have been structurally characterized by single‐crystal X‐ray analysis. In combination with computational methods the structural and thermodynamic reasons for the exceptionally strong binding have been elucidated. The presented rim confinement strategy makes cycloparaphenylenes an attractive supramolecular host family with a favorable, size‐independent read‐out signature and binding capabilities extending beyond fullerene guests.

## Graphical abstract

A series of [*n*]cyclo‐2,7‐pyrenylenes was synthesized. *K*‐region functionalization of the pyrene moieties led to a confined space within those macrocycles. This confined space is especially pronounced for *n=*5 and resulted in a host‐in‐host binding with shape complementary alkaline crown ethers. A multifaceted approach combining computation, spectroscopy, and single‐crystal X‐ray analysis elucidated the binding.

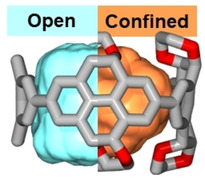

Strained aromatic macrocycles have distinct properties compared to their linear counterparts such as shape persistence, enhanced solubility, and the ability to complex guests in their cavities.[^1^, ^2^, ^3^] Among them, cycloparaphenylenes (CPPs) have gained much interest in the last decade due to their size‐dependent photophysical and electronic properties.[^4^, ^5^, ^6^, ^7^] Several bottom‐up strategies have been developed to synthesize CPPs or derivatives containing hetero‐ or polycyclic aromatic moieties.[^8^, ^9^, ^10^, ^11^, ^12^] Incorporation of such moieties allows to study new functionality within this class of macrocycles and alter their photophysical and electronic properties.[^13^, ^14^, ^15^, ^16^, ^17^] Moreover, the developed synthetic methodology can be used to extend the molecular shape from a macrocycle to a cage.[^18^, ^19^, ^20^, ^21^] While molecular cages intrinsically favor guest inclusion, CPPs and related strained carbon macrocycles have been of limited use as supramolecular hosts, most prominently for fullerenes.[^22^, ^23^, ^24^, ^25^, ^26^, ^27^] However, a higher degree of functionalization in combination with spatial extension of the cyclic structure could enable turning CPPs into a relevant class of supramolecular hosts.

Regarding their rigidity and their size‐dependent UV/Vis absorption and emission, carbon nanorings are unique compared to other supramolecular host structures, such as calix[*n*]arenes, resorcin[*n*]arenes, and cucurbit[*n*]urils.[^28^, ^29^, ^30^, ^31^] Improved guest binding should therefore enhance the applicability of these intrinsically porous and shape persistent molecules in a variety of research areas they are currently investigated for.[^32^, ^33^]

Herein, we report on a series of highly functionalized [*n*]cyclo‐2,7‐(4,5,9,10‐tetrahydro)pyrenylenes (CPYs) as supramolecular hosts with confined binding cavities. The rich chemistry developed on the pyrene scaffold and the reported [4]cyclo‐2,7‐pyrenylene by Yamago and co‐workers (Figure 1, top) make this polycyclic aromatic hydrocarbon a feasible starting point for highly functionalized aromatic macrocycles.[^34^, ^35^] The molecular shape of the pyrene subunit leads to a π‐conjugated concave inner host surface, creating an accessible volume suitable for host–guest interactions.

**Figure 1:**
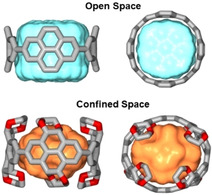
Comparison of the accessible inner space between a non‐functionalized [*n*]cyclo‐2,7‐pyrenylene (CPY) and a highly functionalized [*n*]cyclo‐2,7‐(4,5,9,10‐tetrahydro)pyrenylene derivative (not all ethylene glycol groups are shown for clarity (*n*=4)).

The *K*‐region of pyrene was symmetrically functionalized via Ru‐catalyzed oxidation to obtain pyrene‐4,5,9,10‐tetrone (**5**).^[36]^ Acid‐catalyzed condensation with ethylene glycol readily yielded the wall fragment **4** of the desired macrocyclic structure (Figure 2 a).[^37^, ^38^] We turned to regioselective Ir‐catalyzed C−H borylation of **4** to efficiently produce **3**.^[39]^ Subsequent transmetalation with dichloro(1,5‐cyclooctadiene)platinum(II) gave the macrocyclic precursor **2**.[^11^, ^40^, ^41^] This organometallic precursor is accessible in gram‐scale within four steps from pyrene without the need of column chromatography. Reductive elimination using triphenylphosphine resulted in the targeted CPYs **1_[*n*]_** as a crude reaction mixture of five different macrocycle sizes **1_[4_**_–**8]**_ as observed by MALDI‐TOF MS (Figure 2 b). The different congeners were separated by recycling gel permeation chromatography (GPC).

**Figure 2:**
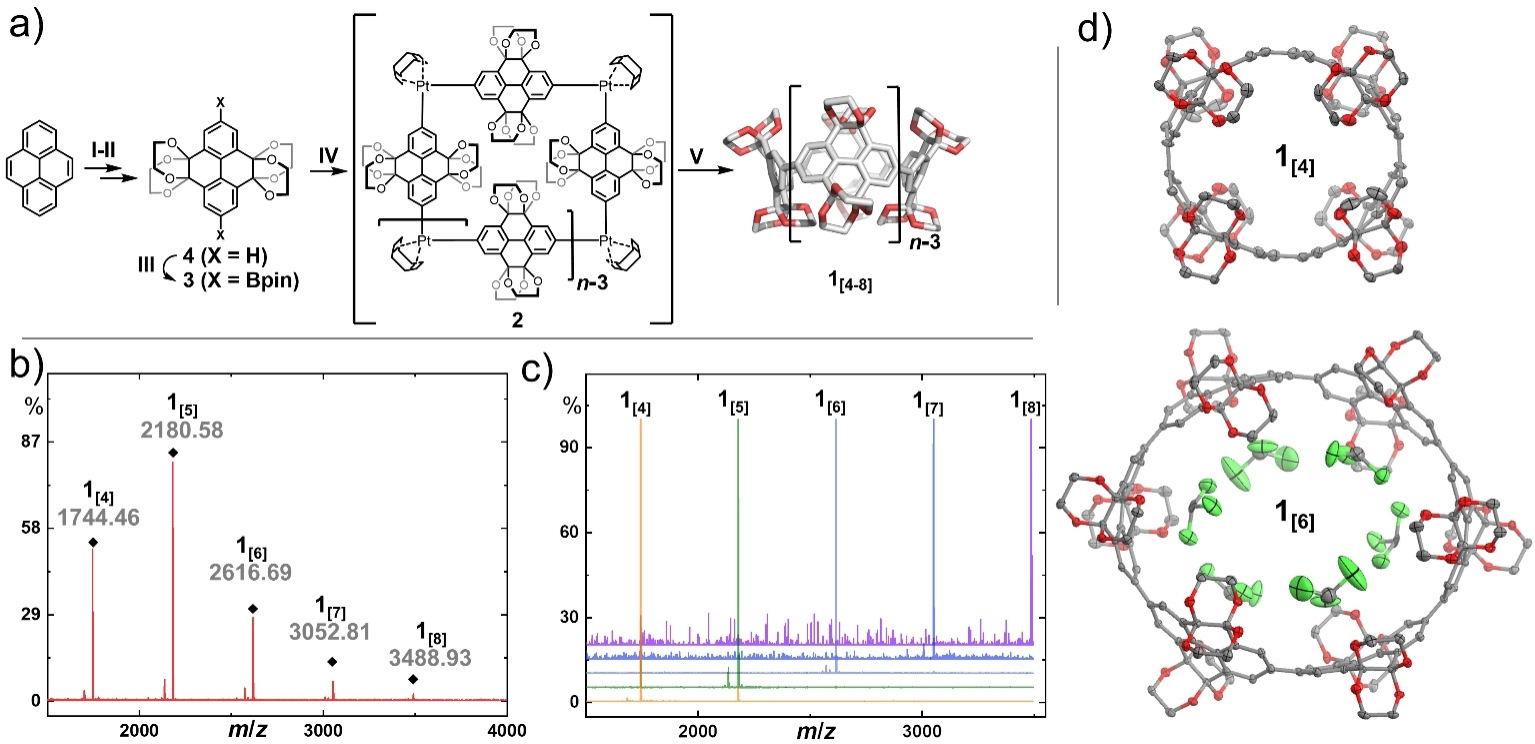
a) Synthesis of **1_[4_**_–**8]**_ starting from pyrene. Reagents and conditions: I) RuCl_3_, NaIO_4_, CH_3_CN/CH_2_Cl_2_/H_2_O, 0 °C, 30 min, then 25 °C, 2 h; II) ethylene glycol, camphorsulfonic acid, MeOH, 120 °C, 24 h; III) 4,4′‐di‐*tert*‐butyl‐2,2′‐bipyridine, [Ir(OMe)(COD)]_2_, bis(pinacolato)diboron, 1,4‐dioxane, 120 °C, 18 h; IV) [PtCl_2_(COD)], CsF, CH_2_Cl_2_, 45 °C, 24 h; V) PPh_3_, 1,2‐dichlorobenzene, 180 °C (microwave), 1 min. MALDI‐TOF mass spectra of b) the filtered crude reaction mixture of step V and c) the isolated macrocycles **1_[4_**_–**8]**_. d) Single‐crystal X‐ray structures of **1_[4]_** and **1_[6]_**, H‐atoms and solvent molecules (except for the ones inside the cavity) are omitted for clarity, thermal ellipsoids are shown at 50 % probability at 100 K.^[77]^ COD=cycloocta‐1,5‐diene.

Formation of various ring sizes is a common feature, observed in several examples following the platinum‐mediated macrocyclization.[^42^, ^43^, ^44^, ^45^, ^46^] While the origin of this effect is still not fully understood, the result is beneficial in the presented example, as a systematic series of hosts with various sizes are obtained in a one‐pot synthesis. During the analysis of the Pt‐containing macrocyclic precursor, we have detected exclusively the mass of tetrameric **2** via MALDI‐TOF MS (see Figure S21 in the Supporting Information), nevertheless formation of other Pt‐ring sizes appears plausible in regard of the literature.[^42^, ^43^, ^44^, ^45^, ^46^] The present triphenylphosphine leads to the formation of Pt(PPh_3_)_4−*m*_, which is known to insert into strain‐activated C−C bonds. Dynamic insertion of active Pt(PPh_3_)_4−*m*_ into the C−C bond between pyrenylene units and subsequent ligand exchange of cyclic fragments can therefore be an explanation for the observed variety of ring sizes.[^12^, ^47^, ^48^, ^49^]

The ^1^H NMR spectra show a single signal in the aromatic region for each macrocycle **1_[*n*]_** owing to their average *D_nh_* (*n*=4–8) symmetry (Figure S8–S19).^[50]^ Due to the limited solubility of **1_[8]_** full characterization by NMR was not possible, pointing towards the feasible limitations in terms of ring size for the presented macrocycles. Unambiguous structural proof was obtained by single‐crystal X‐ray diffraction for **1_[4_**_–**6]**_.^[77]^ Carbon‐rich CPP‐type macrocycles often exhibit a dense herringbone crystal packing, reducing the intrinsic porosity (Figure S34 and S39 for a detailed analysis).[^33^, ^51^, ^52^] In contrast, the solid‐state structures of **1_[4]_** and **1_[6]_** exhibit a slipped–stacked tubular packing presumably caused by the sterically demanding ethylene glycol groups preventing a self‐filling assembly in the solid state. Crystals suitable for X‐ray analysis of **1_[5]_** could only be obtained in the presence of a guest. The defined tubular stacking of this structure can therefore not be compared to the other series members. The crystal structure of **1_[6]_** shows well resolved chloroform molecules, structurally positioned by weak Cl_3_C−H⋅⋅⋅O_host_ hydrogen bonds (3.1–3.3 Å heavy atom distance) and several remarkable C−Cl⋅⋅⋅O_host_ halogen bonds (2.7–3.3 Å; Figure 2 c, for a detailed analysis on the complex interaction network between **1_[6]_** and the solvent CHCl_3_ see Supporting Information Figure S36–S38).

The UV/Vis absorptions of the series **1_[4_**_–**8]**_ show similar absorption maxima *λ*_max_ for all macrocycle sizes (Figure 3 and Table 1), which is a typical property for this class of macrocycles originating from a formally Laporte‐forbidden optical HOMO–LUMO transition.[^6^, ^53^, ^54^] Exceptions to this forbidden transition result in a red‐shifted shoulder of all absorption maxima (400–450 nm). While the entire series of **1_[4_**_–**8]**_ shows rather large apparent Stokes shifts, taking into account the forbidden HOMO–LUMO transition the genuine Stokes shifts are 28–45 nm (0.20–0.31 eV, Table 1). Using time‐dependent density functional theory (TD‐DFT) the calculated oscillator strengths for the HOMO–LUMO transitions of **1_[4_**_–**8]**_ were rather low (*f*<0.2) verifying the forbidden nature of the HOMO–LUMO transition (Figure S79–S83).

**Figure 3:**
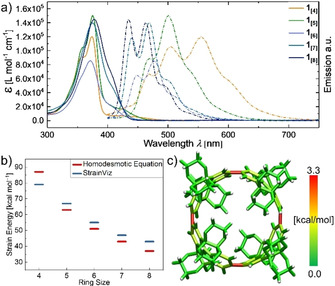
a) UV/Vis absorption (solid lines) and emission (dashed lines) spectra of **1_[4_**_–**8]**_ (CHCl_3_, 25 °C). b) Molecular strain energies calculated using a homodesmotic model and StrainViz (B3LYP/6‐31G(d)). c) Graphical representation of the strain distribution for **1_[4]_** given by StrainViz.

**Table 1:** Photophysical data of the series **1_[4_**_–**8]**_ in CHCl_3_ solution.

	**1_[4]_**	**1_[5]_**	**1_[6]_**	**1_[7]_**	**1_[8]_**
*λ*_max_ [nm]	374	375	371	375	378
*λ*_emission_ [nm]^[a]^	468	468	448	440	435
Stokes shift [eV]	0.28	0.27	0.31	0.24	0.20

[a] Excitation at 375 nm.

The unique photophysical properties of these CPP‐type macrocycles are assigned to their cyclic symmetric structure in combination with the strain necessary to bend their otherwise planar aromatic repeating units. We calculated the strain energy of **1_[4_**_–**8]**_ using an homodesmotic approach and also assessed the ring‐strain energy with StrainViz (Figure 3 b,c).[^55^, ^56^] The results of these calculations generally follow the same and expected trend for the series **1_[5_**_–**8]**_ except for the shortest, most strained tetramer **1_[4]_**, for which the homodesmotic equation calculates a higher strain energy than the results obtained from the StrainViz analysis (Figure 3 b). Strain energies cannot be determined experimentally, yet the calculated results allow for comparison within this set of macrocycles.

A great advantage of StrainViz is the visualization of the strain energy at affected bonds, while classifying the strain energy into bond‐, angle‐, and dihedral strain energy. For CPPs in general, dihedral strain has the highest impact because an average biphenyl angle of circa 44° cannot be fulfilled between all Clar‐sextet units (Figure S84–S88).^[57]^ Comparison of **1_[4]_** to the equivalently sized parent [8]CPP revealed an evenly distributed dihedral strain energy on all eight biphenyl units for [8]CPP but a localized dihedral strain energy over only four pyrene units in **1_[4]_** (visualized by red bonds, Figure 3 c).

Confined spaces within the volumetric centers of **1_[4_**_–**8**]_ are established by the radially converging ethylene groups into the cavity gates of the macrocycles. A crucial parameter in this concept is the ratio of the gate diameter formed by the ethylene glycol groups to the cavity diameter in the center of the macrocycle. If the gate diameter becomes very small the energetic barrier to pass the gate of the cavity might be too high, whereas for a large opening the effect of tight binding to the confined space vanishes (see Supporting Information, Section S7).[^58^, ^59^, ^60^] These considerations are experimentally supported by our finding that **1_[5]_** shows no binding with C_60_ using ^1^H NMR spectroscopy (Figure S75), while the structurally similar [10]CPP (with the same amount of phenylene units as **1_[5]_**) shows very strong binding towards fullerene (*K*_a_=10^10^ m ^−1^).^[61]^ Besides that ratio, the cavity volume is the second crucial parameter, which we visualized for each ring size using the MS Roll suite implemented in X‐Seed^[62]^ (Figure 4 and Section S7). Shape and size of this volume preclude several potential guests according to earlier reports on space filling.[^63^, ^64^] While the ratio between the gate diameter and the central diameter for [*n*]CPPs is 1, this ratio is with 0.64 and 0.67 considerably smaller for **1_[4]_** and **1_[5]_** (Table S8). To investigate the influence on confinement we decided to first study the guest properties of **1_[5]_** since the average gate diameter at the van der Waals surface of **1_[4]_** was calculated to be 3.65 Å, exhibiting a too small opening into the macrocycle's cavity.

**Figure 4:**
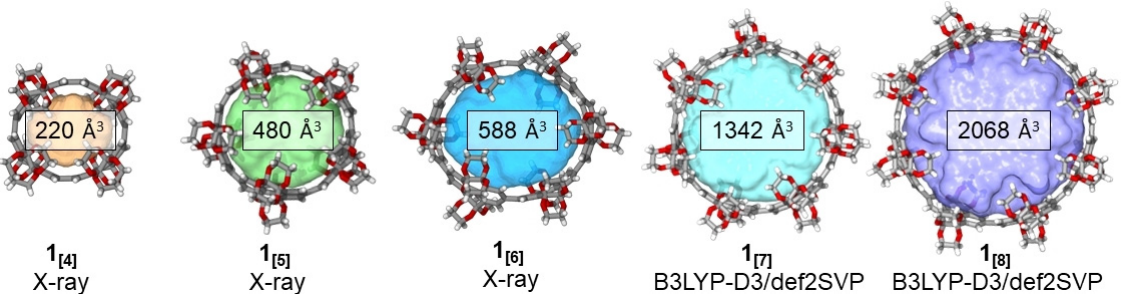
Ball‐and‐stick representation of the X‐ray (**1_[4_**_–**6]**_) and the geometry‐optimized (DFT) structures **1_[7_**_–**8]**_ containing the calculated cavity volume using the MS Roll suite implemented in X‐Seed.

Host‐in‐host complexes are an intriguing starting point in the construction of more complex superstructures.[^65^, ^66^, ^67^] A well‐studied example is the encapsulation of crown ethers or their nitrogen‐containing equivalents into cages or capsular hosts.[^68^, ^69^, ^70^, ^71^, ^72^] We selected crown ethers as guests because they are conformationally flexible allowing them to pass the gate of the macrocycle, and being readily available in various sizes. We first studied the binding of several crown ether metal cation complexes to **1_[5]_** using UV/Vis absorption spectroscopy in CHCl_3_ at 25 °C. Addition of 12‐crown‐4⋅LiCl did not lead to a change in absorption (Figure S61). In contrast, upon addition of 15‐crown‐5⋅Na(BF_4_) a bathochromic shift combined with a narrowing of the absorption band and multiple distinct isosbestic points were observed. The new absorption maximum *λ*_max_ of the complex **1_[5]_**⋅⋅⋅[15‐crown‐5⋅Na(BF_4_)] is red‐shifted by 9 nm as compared to **1_[5]_**. Isothermal UV/Vis absorption titration with addition of up to 54 equivalents of 15‐crown‐5⋅Na(BF_4_) and subsequent non‐linear least‐square curve fitting afforded an association constant *K*_a_=8.0×10^4^ m ^−1^ (Figure 5 a and Figure 5 b, top). A perfect fit in size is further suggested by DFT calculations (Figure S54 and Table S9). To the best of our knowledge, this is the strongest binding observed for a non‐fullerene guest to a CPP or a CPP‐related macrocycle.

**Figure 5:**
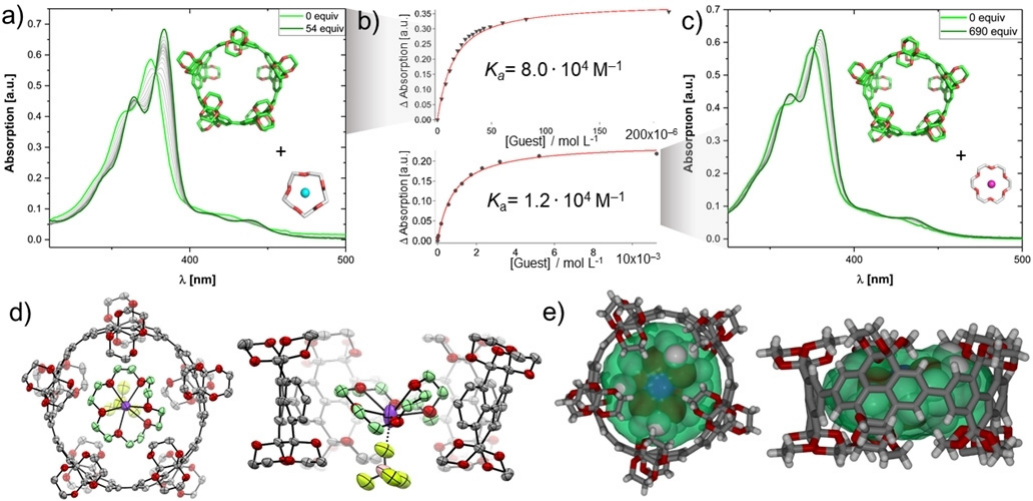
a) Isothermal UV/Vis binding titration of **1_[5]_** (*c*_0_=3.8 μm) upon addition of 15‐crown‐5⋅NaBF_4_ at 25 °C in CHCl_3_. b) Differences in absorption in relation to the guest concentration and curve‐fitting to 1:1 isotherms (solid line). c) Isothermal UV/Vis binding titration of **1_[5]_** (*c*_0_=3.8 μm) upon addition of 18‐crown‐6⋅K(BF_4_) at 25 °C in CHCl_3_. d) Single‐crystal X‐ray structure of **1_[5]_**⋅⋅⋅[18‐crown‐6⋅K(BF_4_)], H‐atoms and solvent molecules are omitted for clarity, thermal ellipsoids shown at 50 % probability, the structure on the right shows the conformational distortion of the 18‐crown‐6 ether.^[77]^ e) Top and side view on X‐ray structure of **1_[5]_**⋅⋅⋅[18‐crown‐6⋅K(BF_4_)], where **1_[5]_** is shown as sticks, 18‐crown‐6⋅K^+^ as van der Waals spheres, and the green surface represents the calculated cavity volume.

To get more structural information on the binding we turned to isothermal ^1^H NMR binding titration, but the necessity for higher concentrations than in the UV/Vis experiments led to peak broadening and precipitation upon addition of 15‐crown‐5⋅Na(BF_4_) to a solution of **1_[5]_** in CDCl_3_ (Figure S74). To investigate the size‐dependency of the binding, we studied the next larger crown ether 18‐crown‐6 and its K^+^ complexes.

Isothermal UV/Vis absorption titration upon addition of 18‐crown‐6⋅K(BF_4_) (Figure 5 c) gave a bathochromic shift of 5 nm for *λ*_max_, accompanied by a narrowing of the absorption, and several distinct isosbestic points. Non‐linear least‐square curve fitting of the absorption titration data afforded an association constant of *K*_a_=1.2×10^4^ m ^−1^ (Figure 5 b, bottom). This only slightly lower *K*_a_ value compared to 15‐crown‐5⋅Na^+^ with **1_[5]_** results from the conformational flexibility of crown ethers. 18‐Crown‐6 can adapt to the rigid host cavity by bending out one ethylene glycol unit, as confirmed by X‐ray crystallography (see below). In contrast, the equally sized but unconfined parent [10]CPP shows significantly smaller binding constants of *K*_a_=103 m ^−1^ and *K*_a_=24 m ^−1^ for 15‐crown‐5⋅Na(BF_4_) and 18‐crown‐6⋅K(BF_4_), respectively (25 °C, CHCl_3_, Figure S70–S73). To complete the series, we investigated the binding of the larger dibenzo‐18‐crown‐6—a guest even too large to be included inside **1_[7]_**—and we observed no binding event (neither for **1_[5]_** nor for **1_[7]_**, see Figure S59 and Figure S69, respectiveley).

Investigating the host abilities of **1_[6]_** towards crown ethers, no pronounced binding was observed for the addition of neither 15‐crown‐5⋅Na(BF_4_) (*K*_a_=28 m ^−1^) nor 18‐crown‐6⋅K(BF_4_) (*K*_a_ too weak to be detected, Figure S66–S68). We assign this result to the drastic increase of the cavity volume by 23 % from **1_[5]_** to **1_[6]_** with the latter therefore requiring much larger guests for reasonable strong binding. Further, no binding event could be determined for **1_[4]_** with the smallest crown ether 12‐crown‐4⋅LiCl, which we assign to the already mentioned very small gate opening, presumably requiring much higher energies to overcome this barrier (Figure S56).

Crystals suitable for synchrotron‐based X‐ray crystallography were obtained for the complex **1_[5]_**⋅⋅⋅[18‐crown‐6⋅K(BF_4_)] (Figure 5 d). The cationic 18‐crown‐6⋅K^+^ is located in the center of the cavity of **1_[5]_** and the ethylene glycol groups of **1_[5]_** embrace the guest complex as a secondary coordination sphere.^[72]^ 18‐crown‐6⋅K^+^ is bound in an unexpected non‐planar conformation with one ethylene glycol unit twisted out‐of‐plane. The energetic penalty of this bent conformation compared to its optimized planar conformation was calculated to be 2.0 kcal mol^−1^ (DFT:B3LYP‐D3/QZVP in vacuum, Figure S45), well compensated by a non‐covalent interaction free enthalpy. Measuring the distance between centroids of the phenyl subunits of **1_[5]_** and the C‐atoms of the crown ether shows ten close contacts (3.5–4.0 Å) assigned to C−H⋅⋅⋅π interactions (Figure S43). Several further C−H⋅⋅⋅O hydrogen bonds were determined between the strongly polarized C−H groups of the crown ether as hydrogen bond donors and the oxygens of the ethylene glycol groups of **1_[5]_** acting as hydrogen bond acceptors (Figure S45). The crystal structure shows a tubular packing of **1_[5]_**, where the void space is filled with 18‐crown‐6⋅K^+^ and the BF_4_^−^ anion. These columnar stacks form a hexagonal circle‐packing motif (Figure S42).[^73^, ^74^]

In summary, we successfully synthesized a series of five [*n*]cyclo‐2,7‐(4,5,9,10‐tetrahydro)pyrenylenes **1_[4_**_–**8]**_ carrying ethylene glycol groups at the outer rim to create a confined space in the center of the macrocycles. The effect of confinement was demonstrated by a significantly weaker binding of 15‐crown‐5⋅Na(BF_4_) in [10]CPP or 18‐crown‐6⋅K(BF_4_) in [10]CPP. We assign the improved binding to the confined space of **1_[5]_** and the additional hydrogen bond acceptors created by the introduction of the dipolar ethylene glycol groups to form a cooperative interaction network.^[75]^ The observed association constant of 15‐crown‐5⋅Na(BF4) to **1_[5]_** is the largest of a non‐fullerene guest to a CPP‐type macrocycle, thereby illustrating the potential of our reported extension strategy to develop these highly fluorescent and shape persistent macrocycles into a new class of supramolecular hosts beyond fullerene guests.[^61^, ^76^]

## Conflict of interest

The authors declare no conflict of interest.

## Supporting information

As a service to our authors and readers, this journal provides supporting information supplied by the authors. Such materials are peer reviewed and may be re‐organized for online delivery, but are not copy‐edited or typeset. Technical support issues arising from supporting information (other than missing files) should be addressed to the authors.

Supplementary
