# Supplementary material for: Confined Spaces in [n]Cyclo‐2,7‐pyrenylenes
Source: Angew Chem Int Ed Engl. 2021 May 24;60(27):14909–14. doi: 10.1002/anie.202102809 (PMC8251724; doi:10.1002/anie.202102809)
Supplement: Supplementary file 1 — Supplementary [file ANIE-60-14909-s001.pdf]

Angewandte Chemie
Eine Zeitschrift der Gesellschaft Deutscher Chemiker

Supporting Information

Confined Spaces in [*n*]Cyclo-2,7-pyrenylenes

*Niklas Grabicki, Khoa T. D. Nguyen, Steffen Weidner, and Oliver Dumele**

anie_202102809_sm_miscellaneous_information.pdf

Table of Content

S1. General Remarks 2

S2. Synthetic Procedures 4

S3. Selected NMR Spectra 9

S4. IR-Spectra of 2 19

S5. Selected Mass Spectra 20

S6. Single-Crystal X-ray Data 26

S6.1 Single-crystal X-ray Data and Structure analysis for $\mathbf{1}_{[4]}$ 31

S6.2 Single-crystal X-ray Data and Structure analysis for $\mathbf{1}_{[6]}$ 34

S6.3 Single-crystal X-ray Data and Structure analysis for $\mathbf{1}_{[5]}$···18-crown-6·K(BF_4) 39

S7. Geometrical Considerations and Volume Analysis of the Hosts [*n*]Cyclo-pyrenylenes $\mathbf{1}_{[4–8]}$ 43

S8. UV/vis Spectroscopy 49

S9. Host–guest Binding Experiments by UV/vis Spectroscopy with $\mathbf{1}_{[4–7]}$ 50

S9.1 Isothermal UV/vis Binding Titrations 53

S9.2 ^{1}H NMR Binding Titration at Slow Exchange of $\mathbf{1}_{[5]}$···(15-crown-5·$NaBF_4$) 60

S9.3 ^{1}H NMR of $\mathbf{1}_{[5]}$ and C_{60} 62

S9.4 ^{1}H NMR of $\mathbf{1}_{[6]}$ and C60 63

S9.5 ^{1}H NMR binding spectroscopy of **[10]CPP** and 15-crown-5·$NaBF_4$ 64

S9.6 ^{1}H NMR binding spectroscopy of **[10]CPP** and 18-crown-6·KBF_4 65

S10. Computational Methods 66

S10.1 Geometry-Optimized Structures and Vertical Transitions 66

S10.2 Strain-Viz calculations[20] 71

S10.3 Strain Calculations *via* Homodesmotic Equation[18] 73

S10.4 Atomic Coordinates of Calculated Structures 75

S11. References 90

S12. Author Contributions 90

S1. General Remarks

Reagents (Acros, AlfaAesar, Sigma-Aldrich, and TCI) were purchased as reagent grade and used without further purification, unless otherwise specified. Solvents for synthesis were dried using a Pure Solv Micro Solvent Purification System from Innovative Technology and stored over molecular sieves 3–4 Å. All non-aqueous reactions were performed in oven-dried glassware and under a N_2 or Ar atmosphere. Automated Medium Pressure Column Chromatography (MPLC) was performed on a Teledyne ISCO CombiFlash R_f 300 system with 200 mL min^{-1} max flow, 200 psi, equipped with integrated ELSD and 200–800 nm UV/Vis variable wavelength detector. Ultra-Performance Liquid Chromatography with Mass Spectrometry coupling (UPLC/MS) was performed with a Waters ACQUITY UPLC H-Class, equipped with a quaternary solvent manager (QSM), a sample manager-flow through needle (SM-FTN), a column heater, a column manager, a ACQUITY UPLC BEH Phenyl 1.7 µm, 2.1x100 mm column, a ACQUITY UPLC BEH C18 1.7 µm, 2.1 x 100 mm column and a photodiode array detector (PDA eλ), and a ACQUITY QDa detector. The mobile phase is a gradient of MeCN/H_2O individually optimized for each separation. Recycling gel permeation chromatography (GPC) was performed with a JAI LC-9210NEXT using trichloromethane with 4% ethanol as a stabilizer as the eluent. The GPC was equipped with the following set of columns: PSS SDV 50 Å, 20x600 mm; PSS SDV 1000 Å, 20x600 mm; Jaigel-2H 5 Å, 20x600 mm. The flow rate was kept at 4.5 mL min^{-1}. Microwave synthesis were performed with a CEM Discover SP Microwave Synthesis System equipped with highly accurate infrared (IR) temperature control, variable speed magnetic stirring, rapid compressed air cooling for quick cool-downs in 10 mL borosilicate vials and with teflon silicon caps. Thin layer chromatography (TLC) was conducted on aluminum sheets coated with SiO_2-60 F_{254} obtained from Merck; visualization with a UV lamp (254 or 366 nm). Evaporation *in vacuo* was performed at 40–60 °C and 700–10 mbar. All products were dried under high vacuum (ca. 10^{-2} mbar) before analytical characterization. Reported yields refer to spectroscopically and chromatographically pure compounds that were dried under high vacuum (ca. 10^{-2} mbar) before analytical characterization, unless otherwise specified. Nuclear magnetic resonance (NMR) spectra were recorded using a Bruker Avance II 300 (300 MHz for ^{1}H and 75 MHz for ^{13}C) and a Bruker Avance II 500 (500 MHz for ^{1}H and 126 MHz for ^{13}C) at 298 K and are reported as follows: chemical shift (δ) in ppm (multiplicity, coupling constant J in Hz, number of protons; assignment). The residual deuterated solvent was used as the internal reference ($CDCl_3$: δ_H = 7.26 ppm, CD_2Cl_2: δ_H = 5.32 ppm, CD_3OD: δ_H = 3.31 ppm, $(CD_3)_2SO$: δ_H = 2.50 ppm; ($CDCl_3$: δ_C = 77.16 ppm, CD_2Cl_2: δ_C = 54.00 ppm, CD_3OD: δ_C = 49.00 ppm, $(CD_3)_2SO$: δ_C = 39.52 ppm; The resonance multiplicity is described as s (singlet), d (doublet), t (triplet), q (quartet), quint (quintet), sept. (septet), m (multiplet), and br. (broad). Melting Points (m.p.) were measured using an MPM-H2 by Schorpp Gerätetechnik in open capillaries and are uncorrected. Infrared (IR) spectra were recorded on a Vertex 70v spectrometer by Bruker equipped with a diamond ATR attachment and are baseline-corrected. The

spectra were measured between 4000 and 400 cm^{-1}. Absorption bands are reported in wavenumbers (cm^{-1}) and their relative intensities described as s (strong), m (medium), or w (weak). Mass spectrometry (MS) and high-resolution mass spectrometry (HRMS) were recorded on a LTQ FTICR Ultra equipped with a 7 Tesla Oxford magnet by Thermo Fisher and a MALDI-TOF Autoflex Max (Bruker Daltonik, Bremen), 355 nm laser, 4000 shot accumulated on 2 different spots, reflector modus, Matrix DCTB (trans-2-[3-(4-tert-Butylphenyl)-2-methyl-2-propenylidene]) 20 mg mL^{-1} in $CHCl_3$ + 1 mg K trifluoroacetate 50 µL matrix solution pre-mixed with 20 µL sample solution (approximately 0.1 mg mL^{-1} in $CHCl_3$) 1 µL droplet deposited of premixed solution on stainless steel target recording using FlexControl software (Bruker). HRMS was performed on a LTQ-FT ULTRA (Thermo), linear ion trap-7 Tesla Fourier-transform ion cyclotron resonance (FTICR) instrument, equipped with electrospray ionization (ESI). Resolution @ *m*/*z* of 400 = 100,000. Samples were infused by a built-in syringe pump at a flow rate of 10 mL min^{-1} and ionized in positive mode. The Xcalibur software was used to assign molecular formulae and to calculate ppm errors.

Ultraviolet–visible absorbance spectroscopy was performed on Agilent Cary 50 and Cary 60 instruments connected to a cryostat from Unisoku Scientific Instruments (temperature accuracy ±0.1 K) in 10 × 10 mm quartz cuvettes with 3 mL volume. Weighing of small quantities was performed on a Sartorius ME5 analytical microbalance. Ultraviolet–Visible Fluorescence Spectroscopy was performed on a Varian Cary Eclipse Fluorescence spectrometer using 10.0×10.0 mm quartz cuvettes. Single-crystal X-ray data were collected on a BRUKER D8 VENTURE area detector with Mo-Kα radiation (λ = 0.71073 Å). Multi-scan absorption corrections implemented in SADABS were applied to the data. If explicitly mentioned the single-crystal X-ray data was collected at the beamline MX14.2 of the BESSYII synchrotron and processed using XDS.[1,2] The structures were solved by intrinsic phasing method (SHELXT-2013)[3] and refined by full matrix least square procedures based on F2 with all measured reflections (SHELXL-2014)[4] in the graphical user interface (OLEX2)[5] with anisotropic temperature factors for all non-hydrogen atoms. If applicable and mentioned, remaining electron density originating from solvent molecules that could not be refined were masked using the solvent mask tool implemented in OLEX2.[6]

S2. Synthetic Procedures

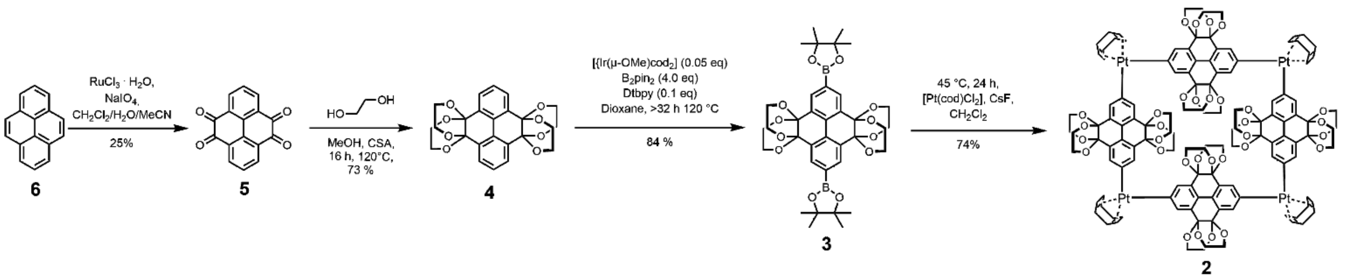

Scheme S1. Overview on the synthetic approach to access Pt-macrocycle **2**.

Pyrene-4,5,9,10-tetrone (5)[7]

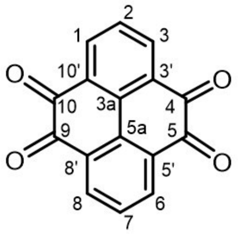

The following procedure was adapted from the literature and modified.[7] A solution of pyrene (20 g, 98.9 mmol) in CH_2Cl_2 (300 mL) was treated with MeCN (300 mL), H_2O (375 mL), and $RuCl_3{\cdot}H_2O$ (2.5 g, 11.1 mmol) and degassed by bubbling N_2 through the solution for 15 min. The suspension was cooled to 3 °C using an ice–water bath, $NaIO_4$ (85 g, 398 mmol) was added and the suspension was stirred with an overhead mechanical stirrer for 15 min. While stirring, the ice bath was removed allowing the suspension to warm up to 25 °C. The suspension was cooled again with an ice bath under nitrogen atmosphere, and treated with an additional portion of $NaIO_4$ (85 g, 398 mmol). The ice bath was removed and the suspension was stirred for 2 h at 25 °C. The organic solvents were removed at 40 °C under reduced pressure and the remaining aqueous phase was vacuum filtered through a Büchner funnel. The obtained solid was purified by liquid extraction using a Soxhlet apparatus (CH_2Cl_2, 7 d). The crude solid suspension (in CH_2Cl_2) was diluted with the same volume of EtOAc and the volatile phase (mostly CH_2Cl_2) was carefully evaporated under reduced pressure. The resulting orange–brown precipitate in the remaining solvent (mostly ethyl acetate) was filtered, washed with EtOAc, and MeOH. Drying under vacuum afforded **5** (7.10 g, 27%) as a brown solid. The characterization data is in agreement with the literature.[7]

R_f = 0.3 (SiO_2; cyclohexane/EtOAc 2:1); ^{1}H NMR (500 MHz, $(CD_3)_2SO$, 25 °C): δ = 8.33 (d, J = 7.80 Hz, 4H, 4 H–C(1,3,6,8)), 7.44 ppm (t, J = 7.80 Hz, 2H, 2 H–C(2,7)); ^{13}C NMR (126 MHz $(CD_3)_2SO$, 25 °C, assignments based on ^{1}H,^{13}C HSQC NMR spectra): δ = 177.14 (C(4,5,9,10)), 134.2 (C(3a,5a)), 131.5 (C(1,3,6,8)), 130.2 ppm (C(2,7)); IR (ATR): $\tilde{\nu}_{max}$ = 3068 (w), 1672 (s), 1558 (s), 1451 (m), 1421 (s), 1336 (m), 1272 (s), 908 (s), 708 cm^{-1} (s); HR-ESI-MS: m/z: 263.0333 ($[M + H]^+$ calcd. for $C_{16}H_7O_4^+$: 263.0339).

Pyrene-4,5,9,10-tetra(ethyleneglycol)ketal (4)[8]

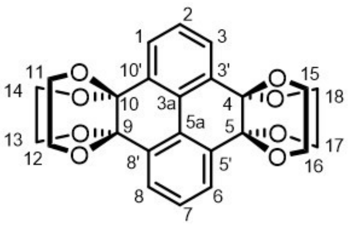

Based on a modified procedure in the literature,[9] a suspension of tetraketone **5** (1.00 g, 3.81 mmol), ethylene glycol (4.00 mL, 71.5 mmol), (+)-camphorsulfonic acid (133 mg, 0.572 mmol), in MeOH (8 mL) was degassed with a constant flow of N_2 in an oven-dried 25 mL-pressure tube, sealed, and heated to 120 °C for 24 h. The mixture was cooled to 23 °C, filtered, the solid was washed with MeOH (3 x 50 mL) and diethyl ether (3 x 50 mL), and dried for 24 h under high-vacuum to obtain **4** (1.22 g, 73%) as a beige solid. The characterization data is in agreement with the literature.[8]

R_f = 0.4 (SiO_2; cyclohexane/EtOAc 2:1); ^{1}H NMR (400 MHz, $CDCl_3$, 25 °C): δ = 7.77 (d, J = 7.77 Hz, 4H, 4 H–C(1,3,6,8)), 7.50 (t, J = 7.72 Hz, 2H, 2 H–C(2,7)), 4.19 (br., 8H, 8 H–C(11–18)), 3.66 ppm (br., 8H, 8 H–C(11–18)); ^{13}C NMR (75 MHz, $CDCl_3$, 25 °C, assignments based on ^{1}H,^{13}C NMR spectra): δ = 133.1 (C(4,5,9,10)), 129.6 (C(2,7)), 129.3 (C(3a,5a)), 127.1 (C(1,3,6,8), 92.7 (C(4,5,9,10)), 61.5 ppm (br., C(11–18)); (FT)ATR-IR: $\tilde{\nu}_{max}$ = 3068 (w), 2988 (w), 2874 (m), 1437 (m), 1267 (m), 1181 (m), 1081 (s), 1012 (s), 957 (s), 816 (m), 744 cm^{-1} (m); HR-ESI-MS: m/z: 439.1386 ([M + H]$^+$calcd. for $C_{24}H_{23}O_8^+$: 439.1387).

2,7-Bis(4,4,5,5-tetramethyl-1,3,2-dioxaborolan-2-yl)-4,5,9,10-tetra(ethyleneglycol)ketal-pyrene (3)[8]

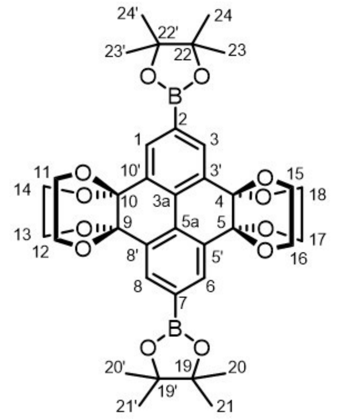

Adapted with changes from the literature,[8,10] a solution of **4** (1.00 g, 2.28 mmol) in degassed dry 1,4-dioxane (23 mL, 22 ppm H_2O by Karl–Fischer titration) was treated with 4,4'-di-*tert*-butyl-2,2'-bipyridine (dtbpy, 61 mg, 228 μmol, 5 mol%), bis(pinacolato)diboron (2.32 g, 9.12 mmol), and $[Ir(OMe)COD]_2$ (76 mg, 114 μmol), degassed for 10 min. until the color changed from dark brown–red to a brighter reddish color, sealed, and heated to 120 °C for 48 h while stirring. The mixture was cooled to 6 °C (fridge)

for 30 min to complete precipitation of a colorless solid. The suspension was filtered, the solid was washed with MeOH and cyclohexane, and dried under high-vacuum for 18 h to afforded **3** (1.36 g, 84%) as a colorless solid. The characterization data is in agreement with the literature.[8]

*R*f = 0.5 (SiO_2; cyclohexane/EtOAc 2:1); ^{1}H NMR (400 MHz, $CDCl_3$, 25 °C): δ = 8.21 (s, 4H, 4 H–C(1,3,6,8)), 4.20 (br., 8H, 8 H–C(11–18)), 3.66 (br., 8H, 8 H–C(11–18), 1.32 ppm (s, 24H, 8 H_3C); ^{13}C NMR (101 MHz, $CDCl_3$, 25 °C, assignments based on ^{1}H,^{13}C-HSQC NMR spectra): δ = 133.5 (C(1,3,6,8)), 132.6 (C(3',5',8',10')), 131.7 (C(3a,5a)), 92.8 (C(4,5,9,10)), 84.1 (C(19,19',22,22'), 63.0–61.0 (br., C(11–18)), 25.0 ppm (C(20,20',21,21',23,23',24,24')) the signal of C(2,7) is hidden by the noise due to quadrupolar relaxation induced by the boron nuclei); (FT)ATR-IR: $\tilde{\nu}_{max}$ = 2975 (s), 1616 (m), 1470 (m), 1373 (s), 1331 (m), 1288 (m), 1261 (s), 1094 (s), 958 (s), 852 (m), 690 cm^{-1} (m); HR-ESI-MS: *m*/*z*: 691.3091 ([*M* + H]$^+$ calcd. for $C_{30}H_{40}B_2O_8^+$: 691.3092).

[*n*]-Cyclo-2,7-(platinum(cyclooctadiene)pyren-4,5,9,10-tetraketal (2)

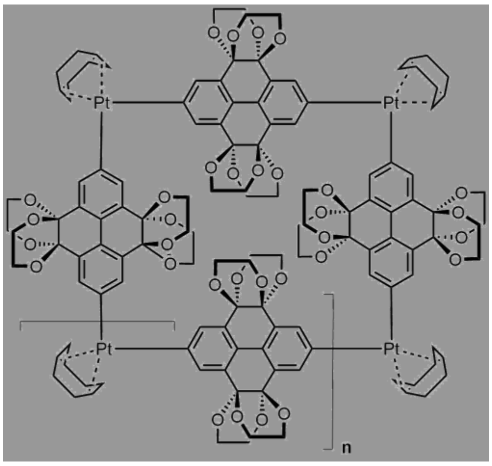

Adapted from the literature,[11] a suspension of dichloro(cycloocta-1,5-diene)platinum(II) (189.7 mg, 507 µmol), CsF (924 mg, 6.1 mmol), and **3** (350.0 mg, 507 µmol) in dry CH_2Cl_2 (140 mL, 5.2 ppm H_2O by Karl-Fischer titration) in an oven-dried 350 mL-pressure tube was degassed using a stream of N_2 for 10 min, sealed, and stirred at 45 °C for 24 h. The mixture was cooled to 23 °C, diluted with cyclohexane (140 mL), and the major volatile CH_2Cl_2 was evaporated to induce precipitation. The resulting suspension was filtered and the pale brown solid was washed with cyclohexane and MeOH. Drying under vacuum afforded **2** (277.5 mg, 74%) as an off-white solid.

*R*f = 0.2 (SiO_2; $CHCl_3$/MeOH 99:1); ^{1}H NMR (400 MHz, CD_2Cl_2, 25 °C): δ = 7.41 (br. s, 16H, 16 H–C(1,3,6,8)), 5.21 (br. s, 16H, 16 H_2–C(cod)), 4.35–2.90 (m, 64H, 16 H–C(11–18)), 2.56 ppm (br. s, 32H, 16 H_2–C(cod); ^{13}C NMR measurements gave no sufficient signal-to-noise ratio due to low solubility; (FT)ATR-IR: $\tilde{\nu}_{max}$ = 3051 (m), 2949 (m), 2873 (m), 2190 (w), 2045 (w), 1438 (m), 1264 (m), 1176 (m), 1091 (s), 1011 (s), 958 (s), 897 (m), 730 (m), 583 cm^{-1} (w); MALDI-TOF-MS: *m*/*z* (%): 2995.39 ([*M* + K]$^+$, calcd. for $C_{120}H_{100}O_{40}K^+$: 2995.66).

[*n*]Cyclo-2,7-(4,5,9,10-tetra(ethyleneglycol)ketal)-pyrenylene (1$_{[n=4–8]}$)

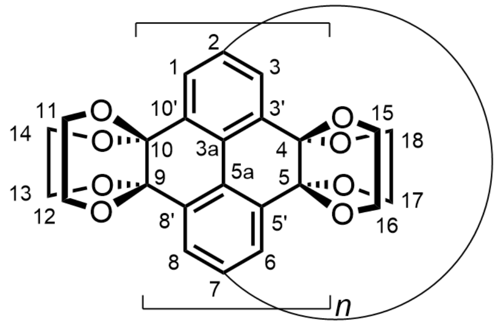

A solution of **2** (50.9 mg, 16.9 µmol) and triphenylphosphine (180.6 mg, 688.5 µmol) in dry 1,2-dichlorobenzene (5 mL) was degassed for 30 min with a constant flow of N_2 under sonication. The reaction vial was sealed and the mixture was heated in a microwave reactor to 180 °C and kept at that temperature for 1 min (typical heating time: 5 min). After cooling to 100 °C, the brown–orange mixture was vacuum-filtered through a PTFE filter (pore size 0.2 µm) to remove insoluble linear oligomers. The filtrate was loaded onto a short pad of silica and was eluted with $CHCl_3$ (ca. 150 mL — major side products and remaining triphenylphosine are first eluted) followed by $CHCl_3$/MeOH 96:4 (ca. 100 mL), which elutes the cyclic product fraction. Evaporation gave a yellow solid (containing macrocyclic products *n* = 4–8 with minor amounts of linear oligomers) that was further purified via GPC (five cycles in sum). Each macrocyclic product fraction (*n* = 4–8) was again purified individually via GPC to get analytically pure compound.

1$_{[n=4]}$: Yellow solid (2.79 mg, 7.3% with regard to the total amount of pyrene fragments added to the reaction). R_f = 0.4 (SiO_2; $CHCl_3$/MeOH 96:4); ^{1}H NMR (500 MHz, $CDCl_3$, 25 °C): δ = 7.90 (s, 16H, 16 H–C(1,3,6,8)), 4.23 (br. m, 16H), 4.11 (br. m, 16H), 3.66 (br. m, 16H, 16 H–C), 3.58 ppm (br. m, 16H); ^{13}C NMR (126 MHz, $CDCl_3$, 25 °C, assignments based on ^{1}H,^{13}C-HSQC and HMBC NMR spectra): δ = 139.5 (C(3a,5a)), 134.0 (C(2,7 or 3’,5’,8’10’)), 128.23 (C(1,3,6,8)), 125.7 (C(2,7 or 3’,5’,8’10’)), 93.2 (C(4,5,9,10)) 61.8 (C(11–18)), 61.4 ppm (C(11–18)); MALDI-TOF-MS: *m*/*z*: 1744.42 ([*M*], calcd. for $C_{96}H_{80}O_{32}$: 1744.46).

1$_{[n=5]}$: Yellow solid (8.2 mg, 12.3% with regard to the total amount of pyrene fragments added to the reaction). R_f = 0.3 (SiO_2; $CHCl_3$/MeOH 96:4); ^{1}H NMR (500 MHz, $CDCl_3$, 25 °C, assignments based on ^{1}H,^{13}C-HSQC and HMBC NMR spectra): δ = 7.89 (s, 16H, 16 H–C(1,3,6,8)), 4.19 (broad s, 20H, 16 H–C(out of C11–C18)), 4.13 (broad s, 20H, 16 H–C(out of C11–C18)), 3.69 (broad s, 20H, 16 H–C(out of C11–C18)), 3.65 ppm (broad s, 20H, 16 H–C(out of C11–C18)); ^{13}C NMR (126 MHz, $CDCl_3$, 25 °C, assignments based on ^{1}H,^{13}C-HSQC and HMBC NMR spectra): δ = 140.2 (C(3a,5a)), 134.0 (C(2,7 or 3’,5’,8’10’)), 129.1 (C(1,3,6,8)), 125.6 (C(2,7 or 3’,5’,8’10’)), 93.0 (C(4,5,9,10)), 61.7 (C(11–18)), 61.4 ppm (C(11–18)); MALDI-TOF-MS: *m*/*z*: 2180.57 ([*M*], calcd. for $C_{120}H_{100}O_{40}$: 2180.59).

1$_{[n=6]}$: Yellow solid (5.8 mg, 8.7% with regard to the total amount of pyrene fragments added to the reaction). R_f = 0.3 (SiO_2; $CHCl_3$/MeOH 96:4); ^{1}H NMR (500 MHz, $CDCl_3$, 25 °C, assignments based on ^{1}H,^{13}C HMBC NMR spectra): δ = 7.89 (s, 24H, 16 H–C(1,3,6,8)), 4.17 (broad s, 48H, 16 H–C(out of C11–C18)), 3.70 (broad s, 24H, 16 H–C(out of C11–C18)), 3.60 ppm (broad s, 24H, 16 H–C(out of C11–C18)); ^{13}C NMR measurements gave no sufficient signal-to-noise ratio due to low solubility; ^{1}H,^{13}C-HMBC NMR spectra, extracted ^{13}C signals (600 MHz, $CDCl_3$, 25 °C) δ = 141.2, 128.8, 125.4, 92.7 ppm; MALDI-TOF-MS: *m*/*z* (%): 2616.76 (**1**$_{[6]}$, [*M*], calcd. for $C_{144}H_{120}O_{48}$: 2616.70).

1$_{[n=7]}$: Yellow solid (5.9 mg, 8.8% with regard to the total amount of pyrene fragments added to the reaction). R_f = 0.3 (SiO_2; $CHCl_3$/MeOH 96:4); ^{1}H NMR (500 MHz, $CDCl_3$, 25 °C, assignments based on ^{1}H,^{13}C-HSQC NMR spectra): δ = 7.98 (s, 28H, 16 H–C(1,3,6,8)), 4.20 (broad s, 56H, 16 H–C(out of C11–C18)), 3.71 ppm (broad s, 56H, 16 H–C(out of C11–C18)); ^{13}C NMR (126 MHz, $CDCl_3$, 25 °C, assignments based on ^{1}H,^{13}C-HSQC, ^{1}H,^{13}C-HMBC and HMBC NMR spectra): δ = 140.8 (C(3a,5a)), 133.9 (C(2,7 or 3',5',8'10')), 128.8 (C(1,3,6,8)), 125.7 (C(2,7 or 3',5',8'10')), 92.9 (C(4,5,9,10)), 61.7 ppm (C(11–18)); MALDI-TOF-MS: *m*/*z*: 3052.95 ([*M*], calcd. for $C_{168}H_{140}O_{56}$: 3052.81).

1$_{[n=8]}$: Beige solid (2.1 mg, 3.1% with regard to the total amount of pyrene fragments added to the reaction). R_f = 0.2 (SiO_2; $CHCl_3$/MeOH 96:4); ^{1}H NMR (500 MHz, $CDCl_3$, 25 °C): δ = 8.04 (s, 16H, 16 H–C(1,3,6,8)), 4.23 (broad s, 16H, 16 H–C(out of C11–C18)), 3.68 ppm (broad s, 16H, 16 H–C(out of C11–C18)); ^{13}C NMR measurements gave no sufficient signal-to-noise ratio due to low solubility. MALDI-TOF-MS: *m*/*z*: 3489.10 ([*M*], calcd. for $C_{192}H_{160}O_{64}$: 3488.93).

The low yields and the laborious purification process led to variations in yields for the individual ring sizes. Therefore, an average yield from three reaction runs was calculated (Table S1).

Table S1. Scale and yields for **1**$_{[4–8]}$ of three independently performed reactions.

Scale	Yield **1**$_{[4]}$	Yield **1**$_{[5]}$	Yield **1**$_{[6]}$	Yield **1**$_{[7]}$	Yield **1**$_{[8]}$
100 mg	3.8%	4.1%	2.6%	1.6%	0.7%
150 mg	6.2%	8.7%	7.3%	10.3%	4.3%
100 mg	7.3%	12.3%	8.7%	8.8%	3.1%
ø	5.8%	8.4%	6.2%	6.9%	2.7%

S3. Selected NMR Spectra

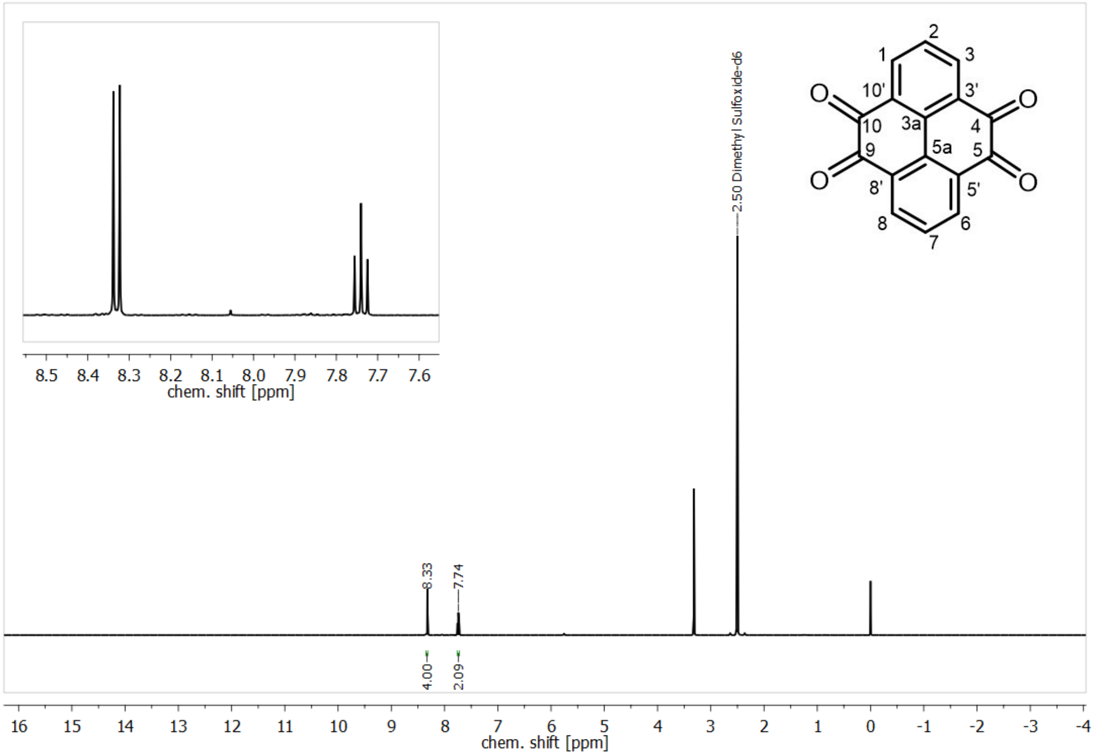

Figure S1. ^{1}H NMR spectrum (500 MHz, 298 K) of pyrene-4,5,9,10-tetrone (**5**) in $(CD_3)_2SO$.

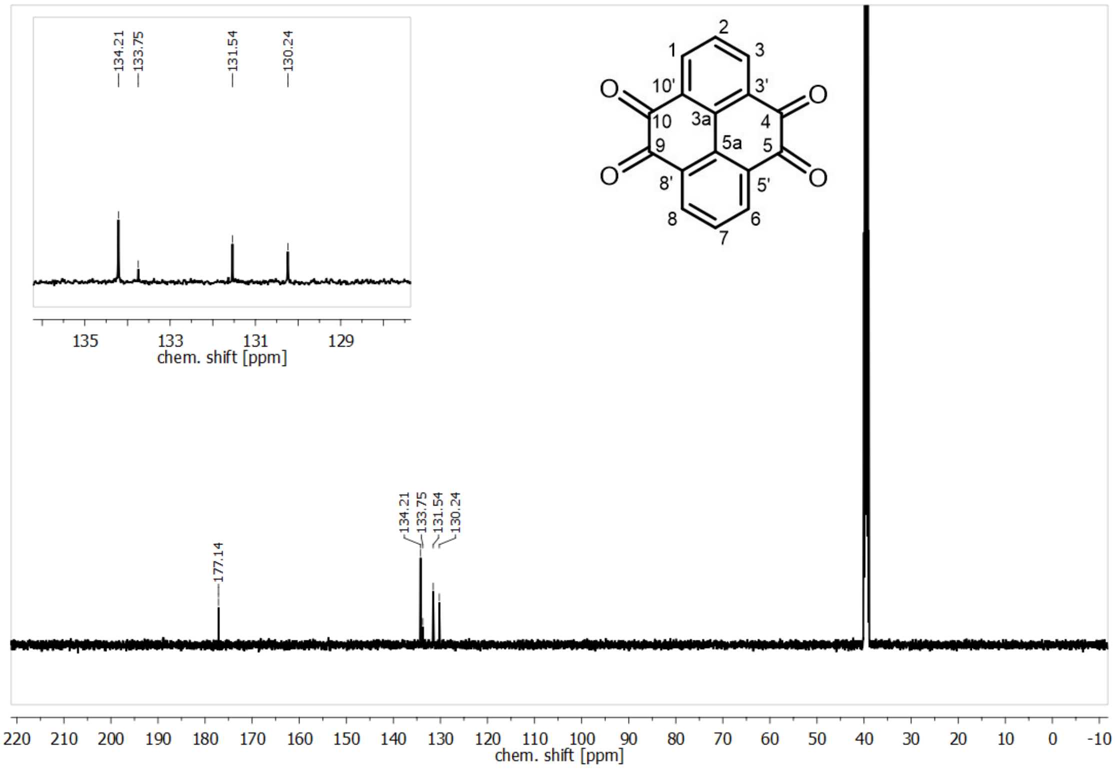

Figure S2. ^{13}C NMR spectrum (126 MHz, 298 K) of pyrene-4,5,9,10-tetrone (**5**) in $(CD_3)_2SO$.

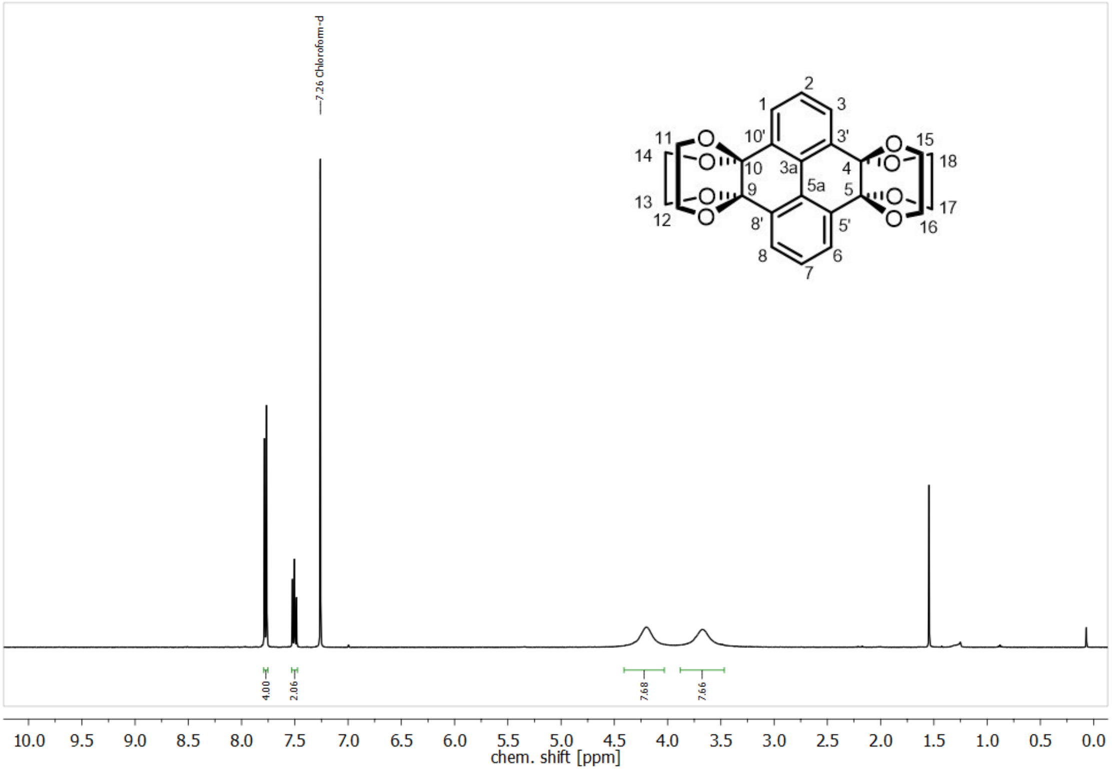

Figure S3. ^{1}H NMR spectrum (400 MHz, 298 K) of pyrene-4,5,9,10-tetra(ethyleneglycol)ketal (**4**) in $CDCl_3$.

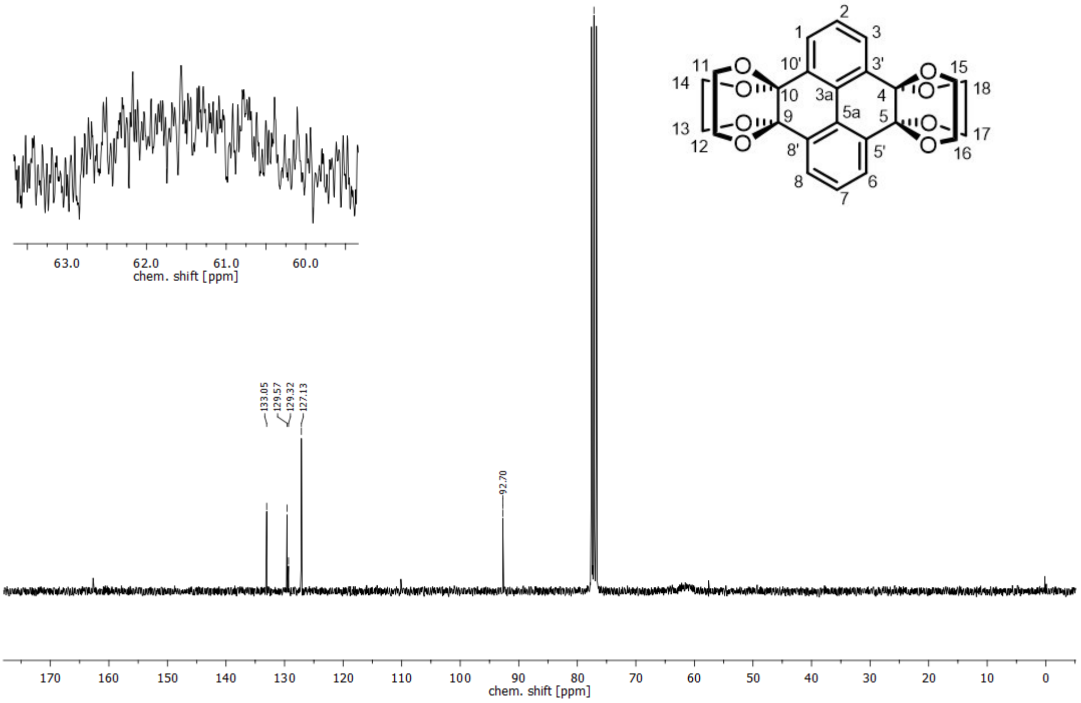

Figure S4. ^{13}C NMR spectrum (75 MHz, 298 K) of pyrene-4,5,9,10-tetra(ethyleneglycol)ketal (**4**) in $CDCl_3$.

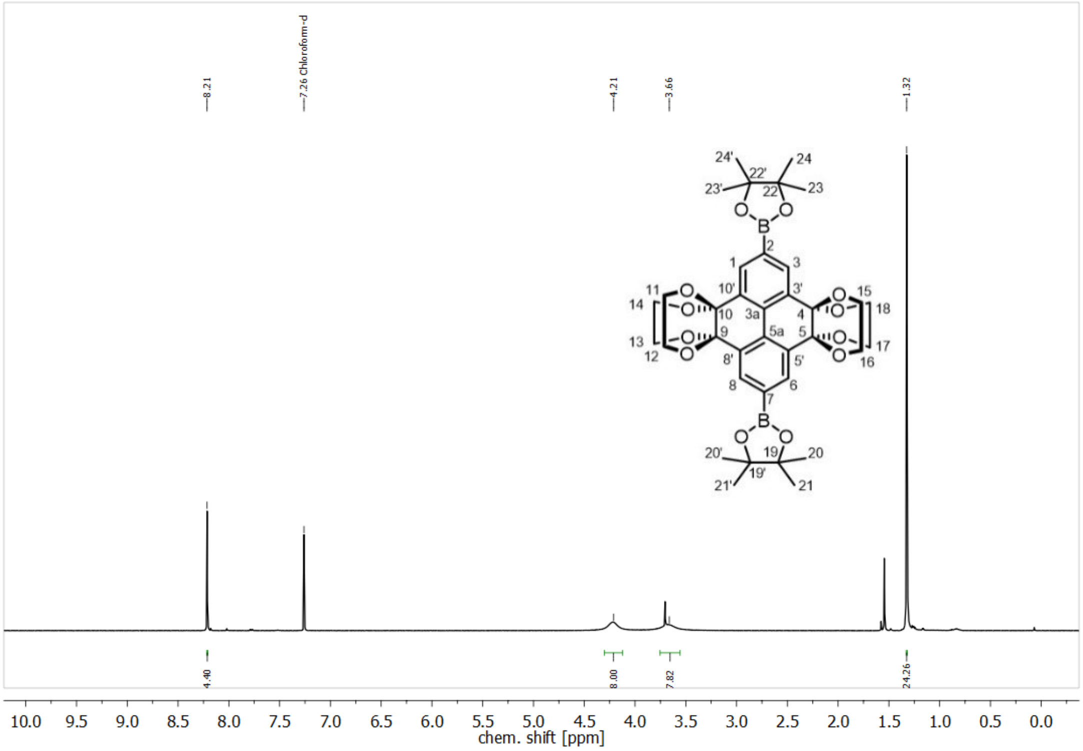

Figure S5: ^{1}H NMR spectrum (400 MHz, 298 K) of 2,7-bis-pinacolatoboron-4,5,9,10-tetra(ethyleneglycol)ketal-pyrene (**3**) in $CDCl_3$.

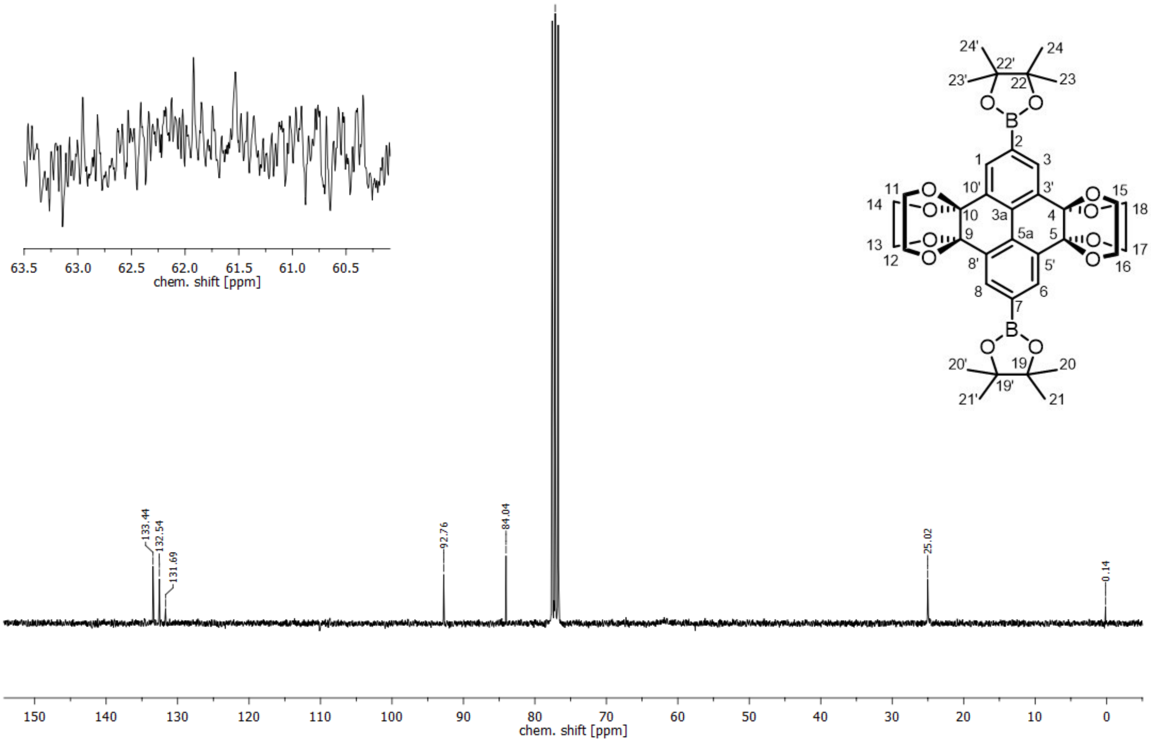

Figure S6. ^{13}C NMR spectrum (75 MHz, 298 K) of 2,7-bis-pinacolatoboron-4,5,9,10-tetra(ethyleneglycol)ketal-pyrene (**3**) in $CDCl_3$.

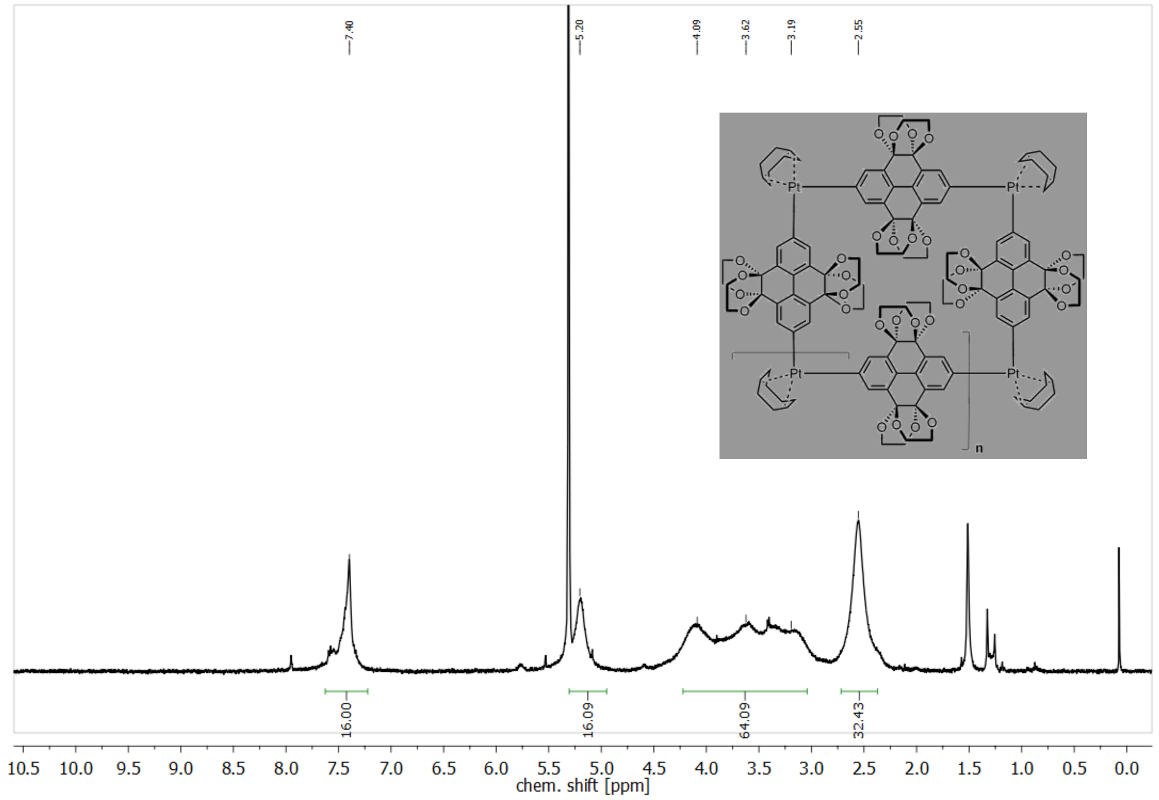

Figure S7. ^{1}H NMR spectrum (400 MHz, 298 K) of [4]cyclo-2,7-(platinum-(cyclooctadiene)pyren-4,5,9,10-tetraketal (**2**) in CD_2Cl_2.

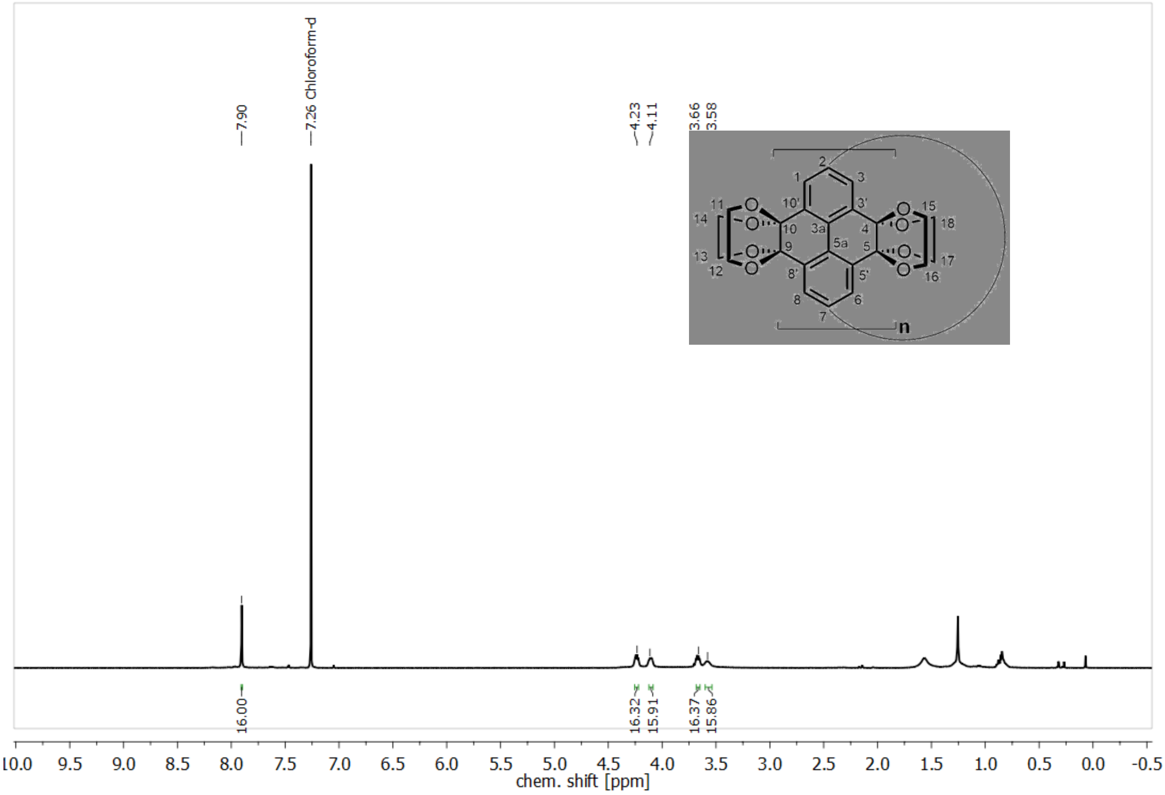

Figure S8. ^{1}H NMR spectrum (500 MHz, 298 K) of [4]cyclo-2,7-pyren-(4,5,9,10-tetra(ethyleneglycol)ketal)ylene (**1**$_{[4]}$) in $CDCl_3$.

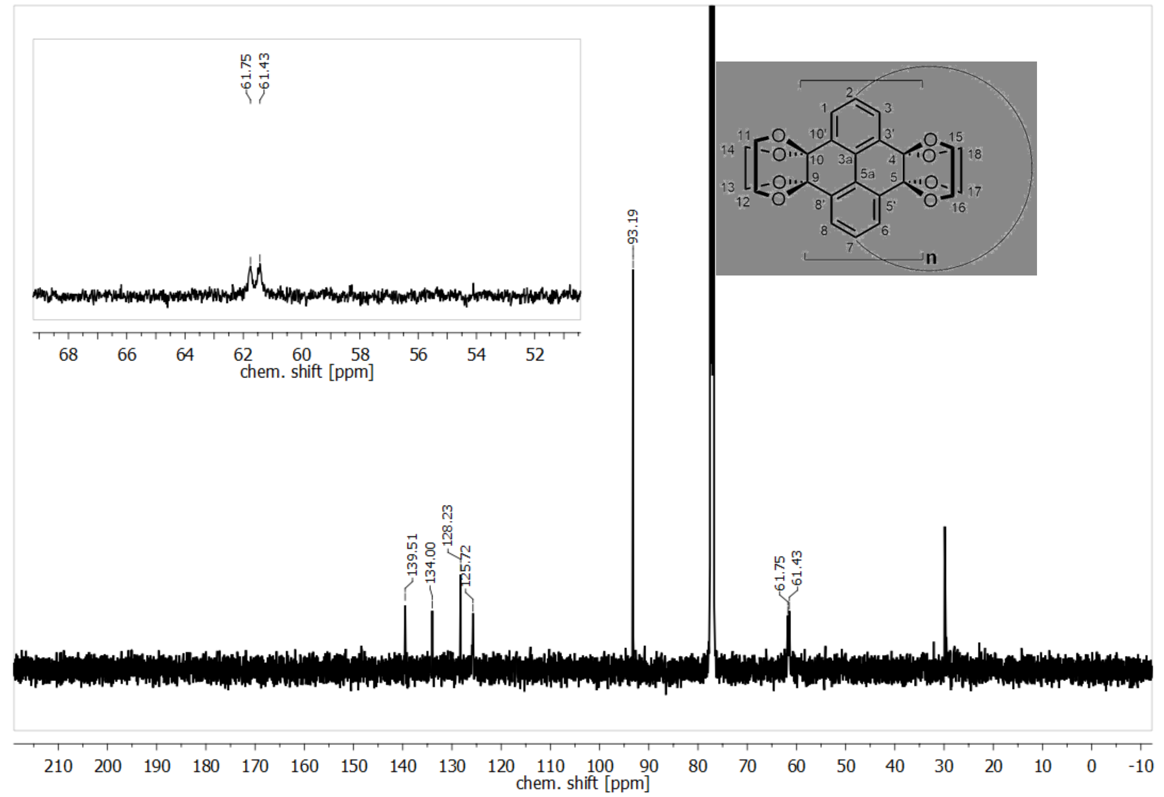

Figure S9. ^{13}C NMR spectrum (500 MHz, 298 K) of [4]cyclo-2,7-pyren-(4,5,9,10-tetra(ethyleneglycol)ketal)ylene (**1**$_{[4]}$) in $CDCl_3$.

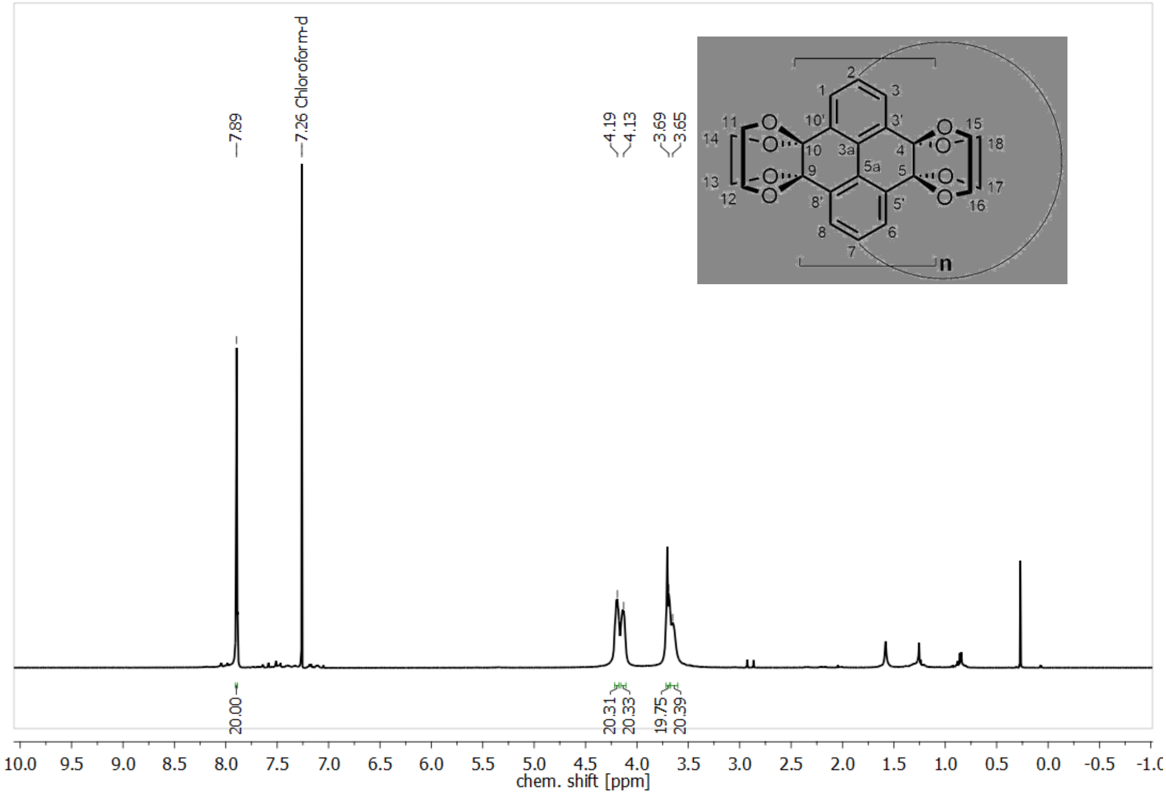

Figure S10. ^{1}H NMR spectrum (500 MHz, 298 K) of [5]cyclo-2,7-pyren-(4,5,9,10-tetra(ethyleneglycol)ketal)ylene (**1**$_{[5]}$) in $CDCl_3$.

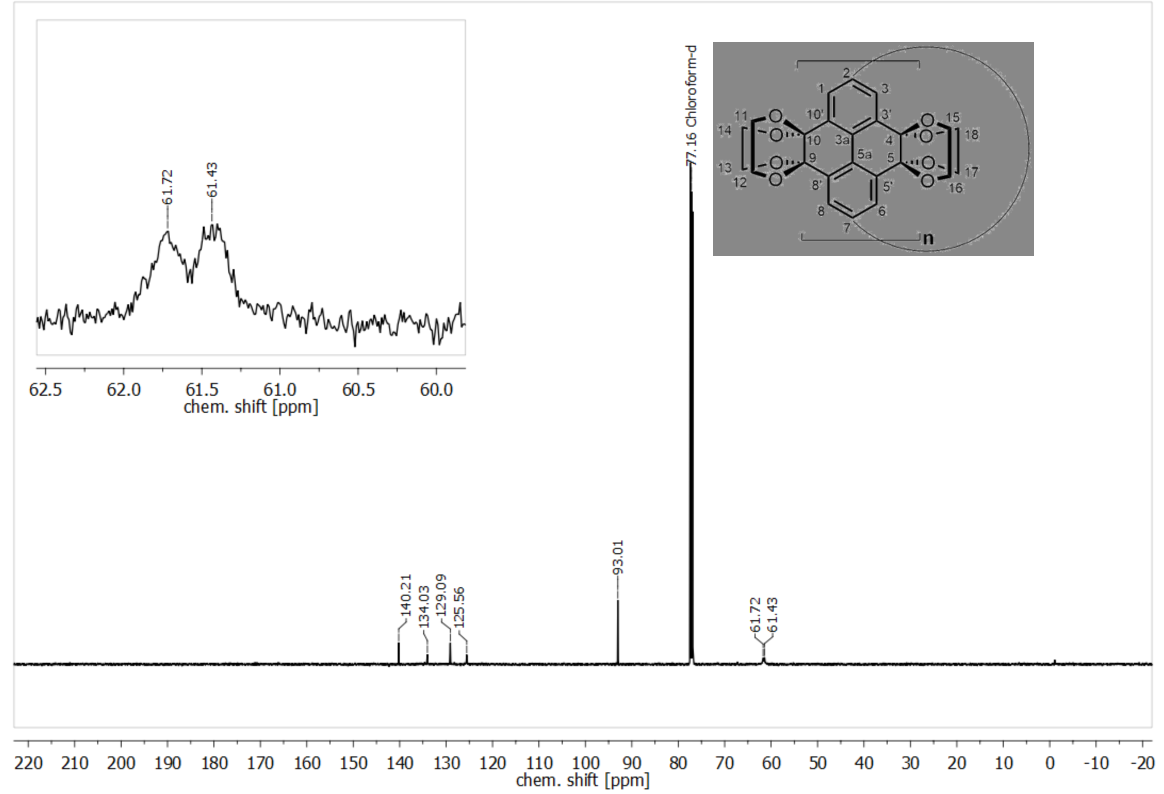

Figure S11. ^{13}C NMR spectrum (126 MHz, 298 K) of [5]-2,7-pyren-(4,5,9,10-tetra(ethyleneglycol)ketal)ylene (**1**$_{[5]}$) in $CDCl_3$.

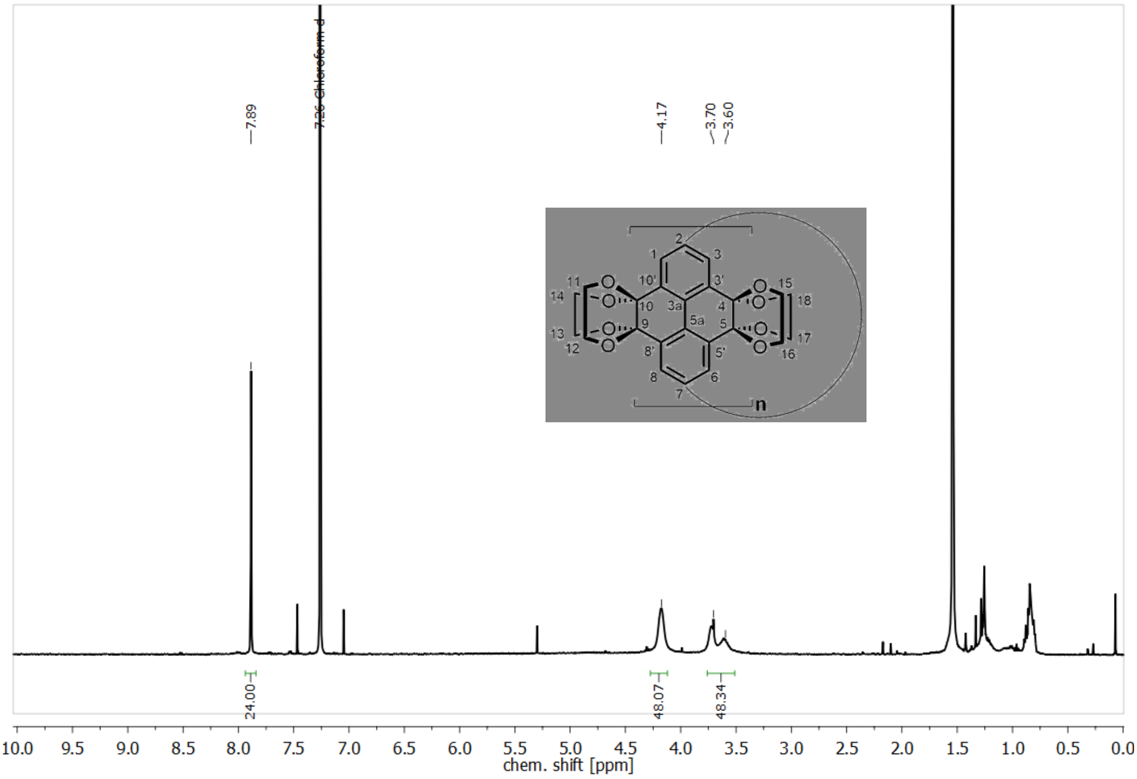

Figure S12. ^{1}H NMR spectrum (500 MHz, 298 K) of [6]cyclo-2,7-pyren-(4,5,9,10-tetra(ethyleneglycol)ketal)ylene (**1**[6]) in $CDCl_3$. (solvent residues 5.32 CH_2Cl_2, 1.56 H_2O, and impurities (grease) 0.8 and 1.3 ppm).

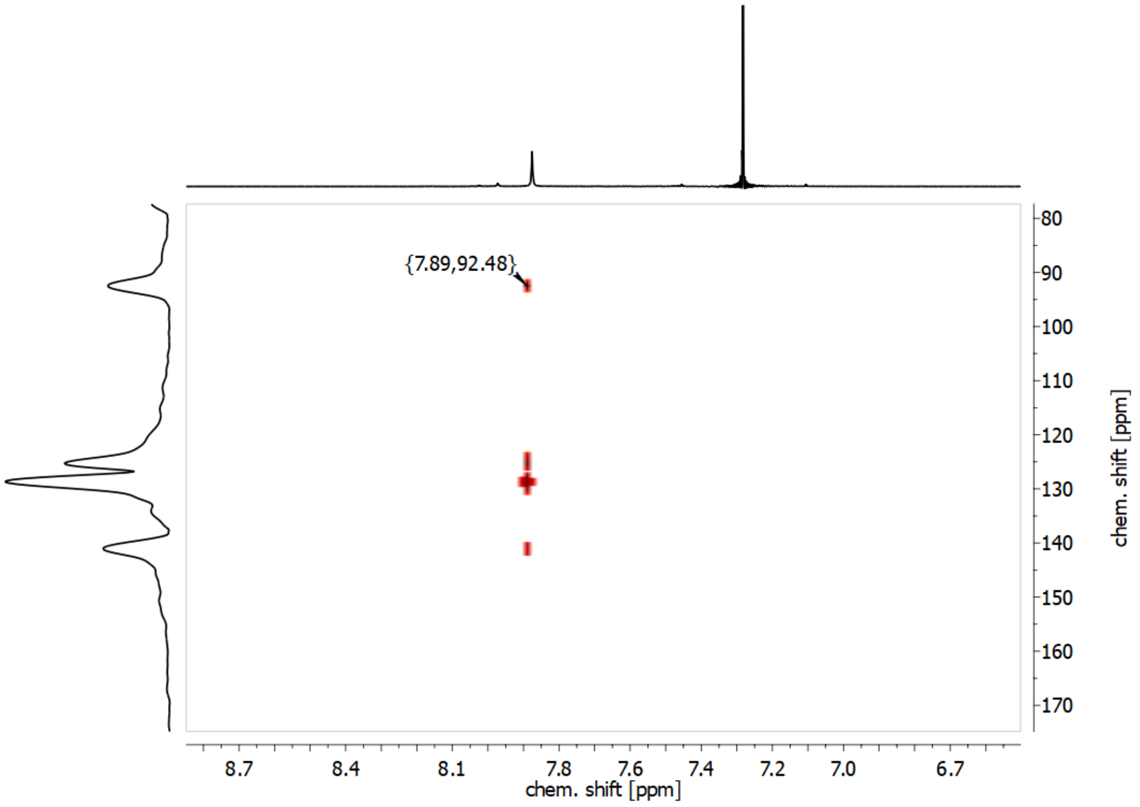

Figure S 13. Enlarged ^{1}H ^{13}C HMBC NMR spectrum (600 MHz, 150 MHz, 298 K) of [6]cyclo-2,7-pyren-(4,5,9,10-tetra(ethyleneglycol)ketal)ylene (**1**[6]) in $CDCl_3$/MeOD (99:1).

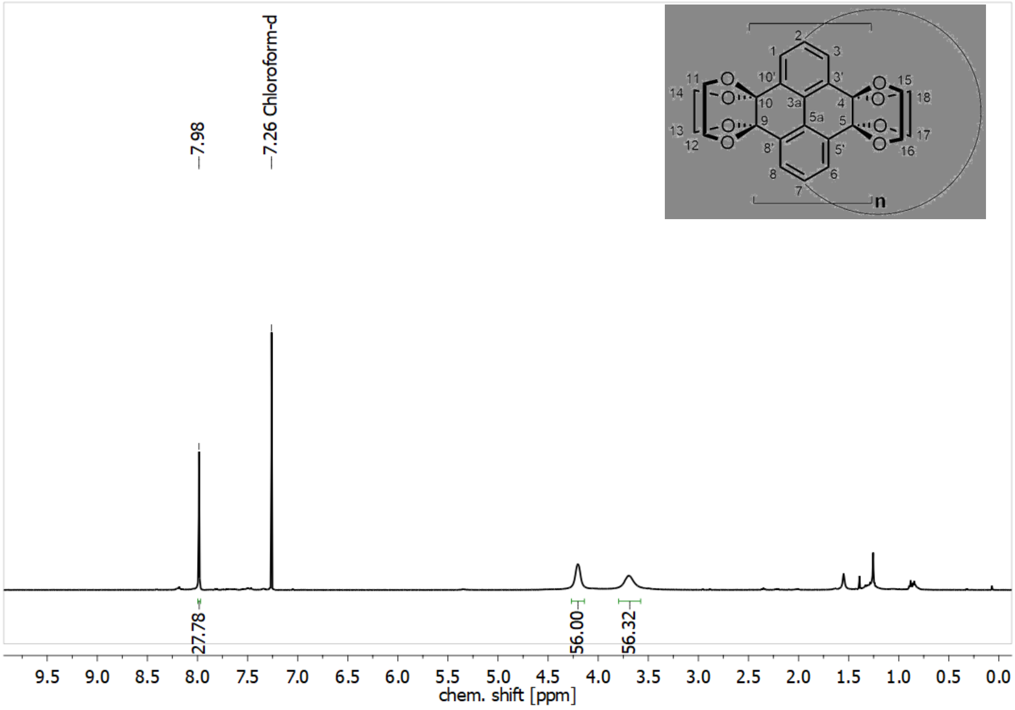

Figure S14. ^{1}H NMR spectrum (500 MHz, 298 K) of [7]cyclo-2,7-pyren-(4,5,9,10-tetra(ethyleneglycol)ketal)ylene (**1**$_{[7]}$) in $CDCl_3$.

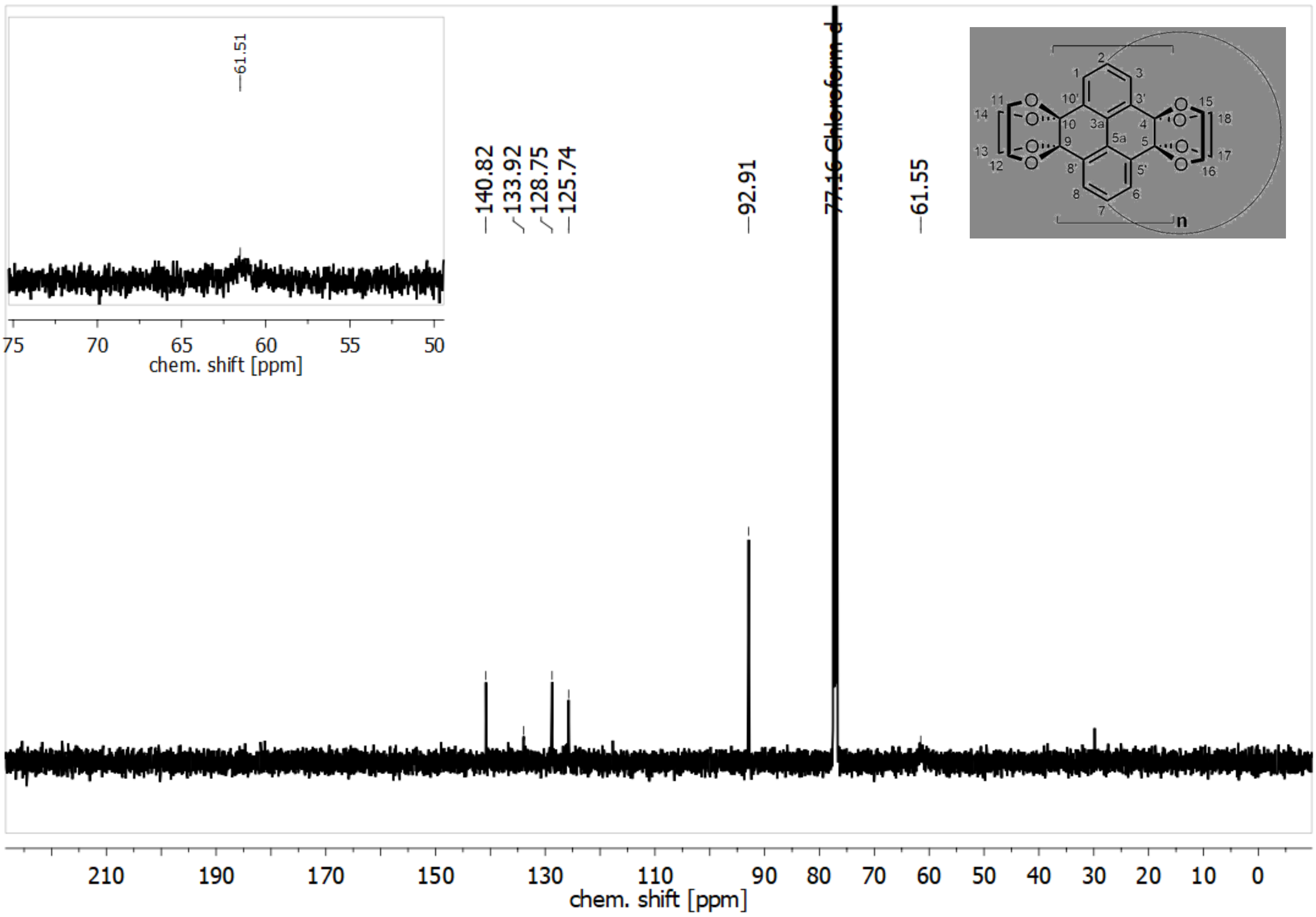

Figure S15. ^{13}C NMR spectrum (126 MHz, 298 K) of [7]cyclo-2,7-pyren-(4,5,9,10-tetra(ethyleneglycol)ketal)ylene (**1**$_{[7]}$) in $CDCl_3$.

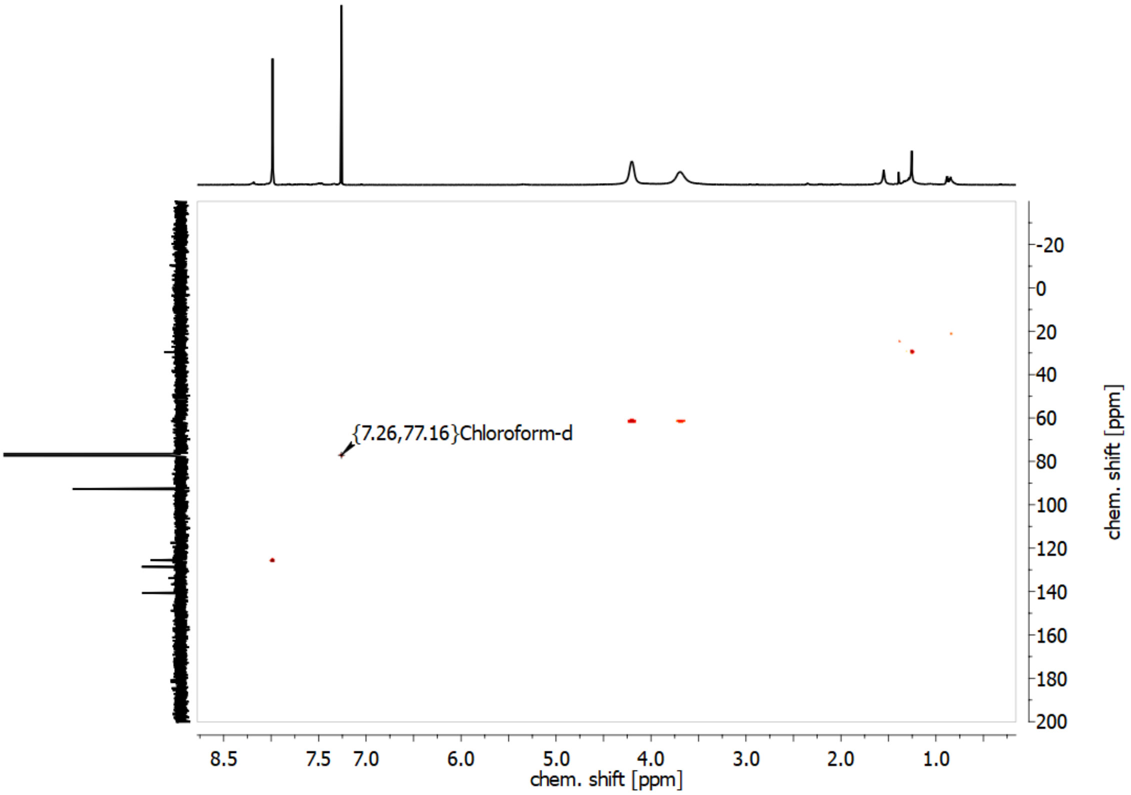

Figure S16. ^{1}H ^{13}C HSQC NMR spectrum (500 MHz, 126 MHz, 298 K) of [7]cyclo-2,7-pyren-(4,5,9,10-tetra(ethyleneglycol)ketal)ylene (**1**$_{[7]}$) in $CDCl_3$.

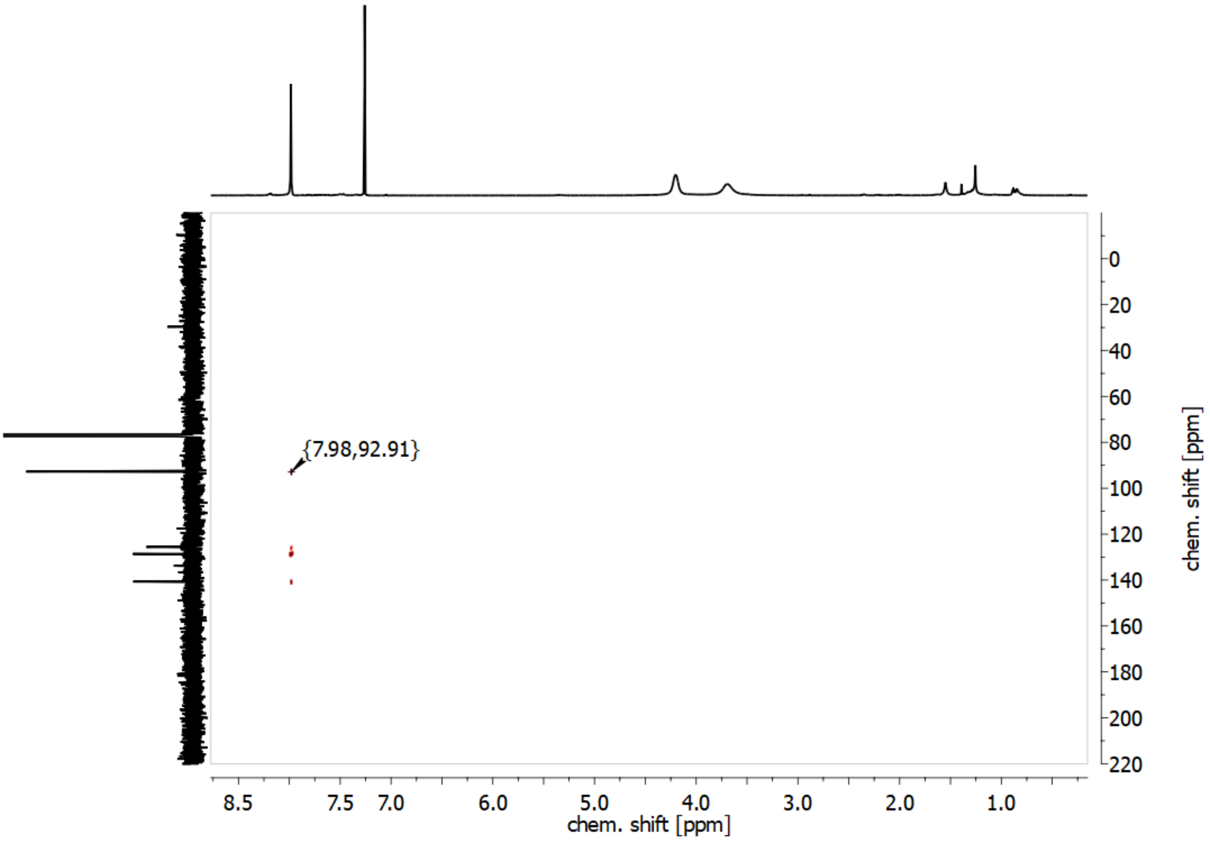

Figure S17. ^{1}H ^{13}C HMBC NMR spectrum (500 MHz, 126 MHz, 298 K) of [7]-2,7-pyren-(4,5,9,10-tetra(ethyleneglycol)ketal)ylene (**1**$_{[7]}$) in $CDCl_3$.

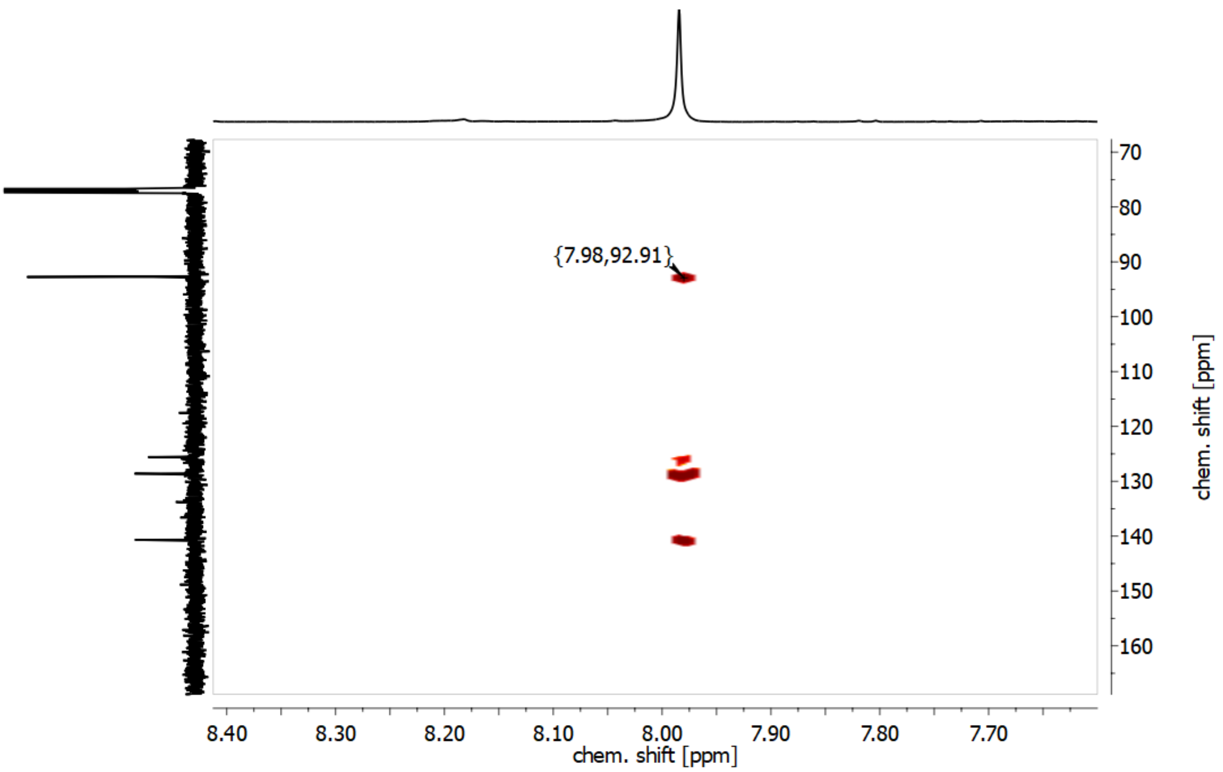

Figure S18. Enlarged ^{1}H ^{13}C HMBC NMR spectrum (500 MHz, 126 MHz, 298 K) of [7]-2,7-pyren-(4,5,9,10-tetra(ethyleneglycol)ketal)ylene (**1**$_{[7]}$) in $CDCl_3$.

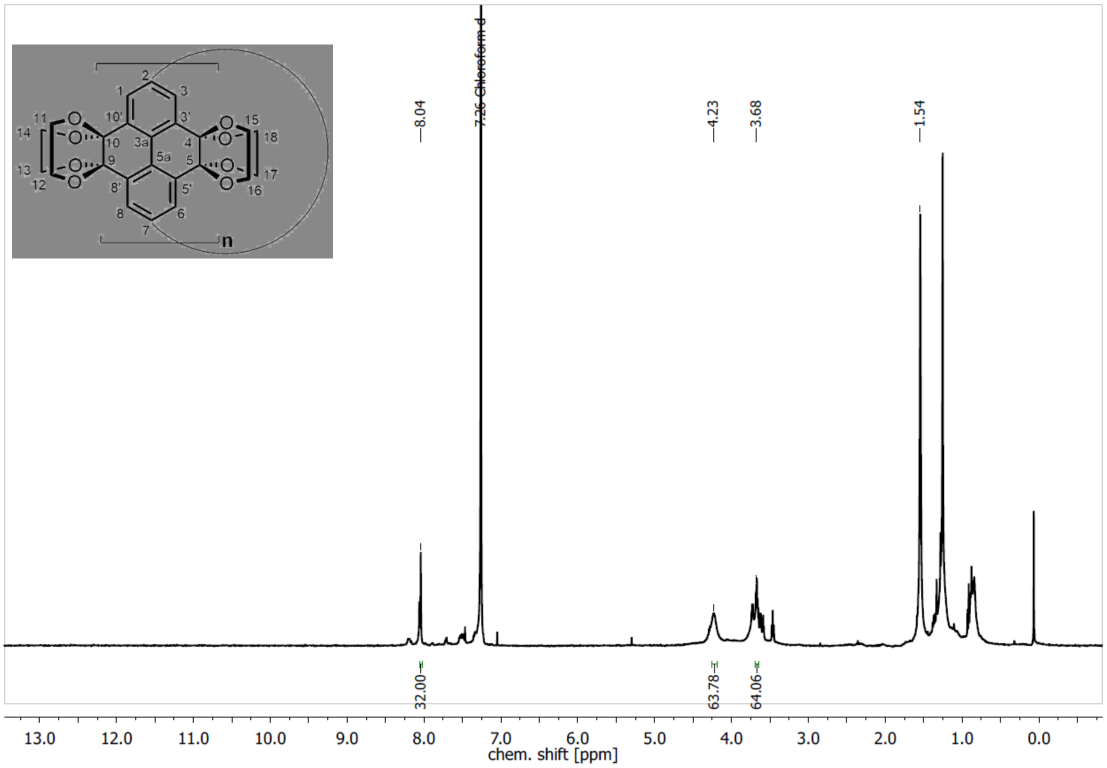

Figure S19. ^{1}H NMR spectrum (500 MHz, 298 K) of [8]-2,7-pyren-(4,5,9,10-tetra(ethyleneglycol)ketal)ylene (**1**$_{[8]}$) in $CDCl_3$.

S4. IR-Spectra of 2

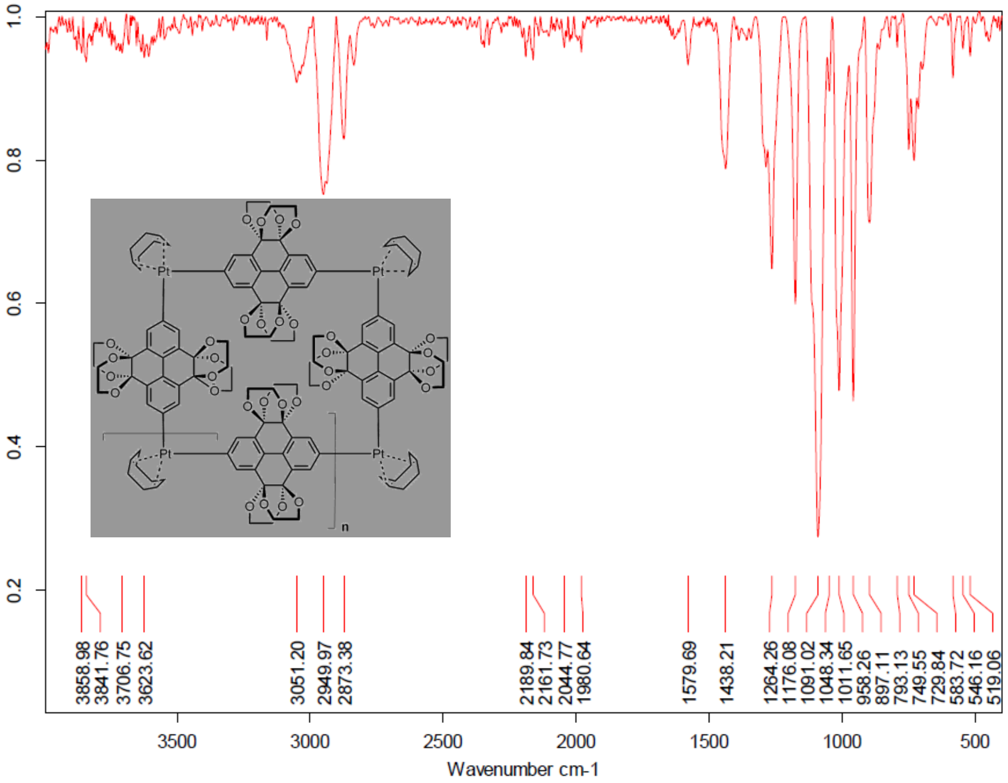

Figure S20. (FT)AT-IR spectrum of [4]-cyclo-2,7-(Pt(cyclooctadiene)pyren-4,5,9,10-tetra(ethyleneglycol)ketal **(2)**.

S5. Selected Mass Spectra

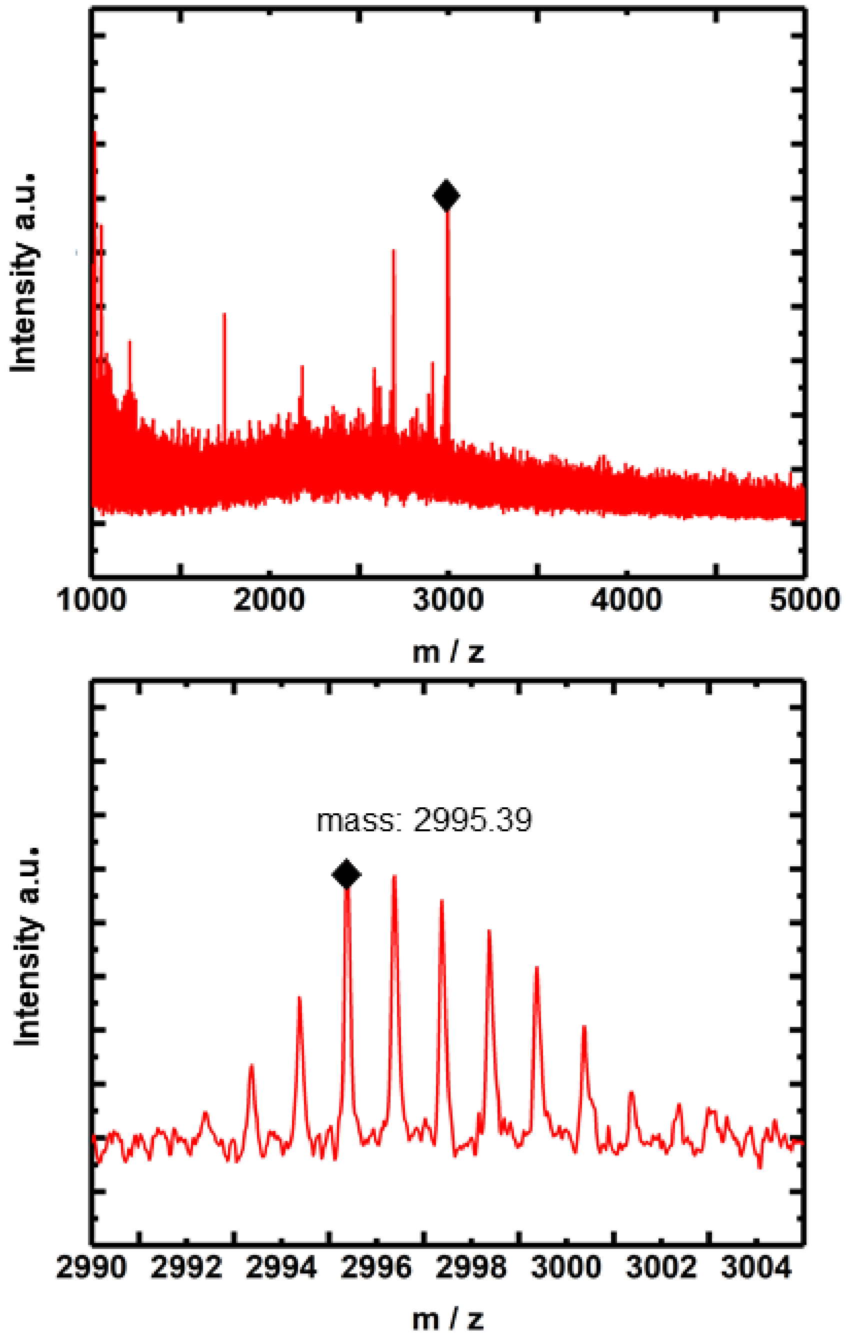

Figure S21. MALDI-TOF mass spectra of [4]cyclo-2,7-(platinum(cyclooctadiene)pyren-4,5,9,10-tetra(ethyleneglycol)ketal (top) and enlarged representation to show the isotope pattern (bottom).

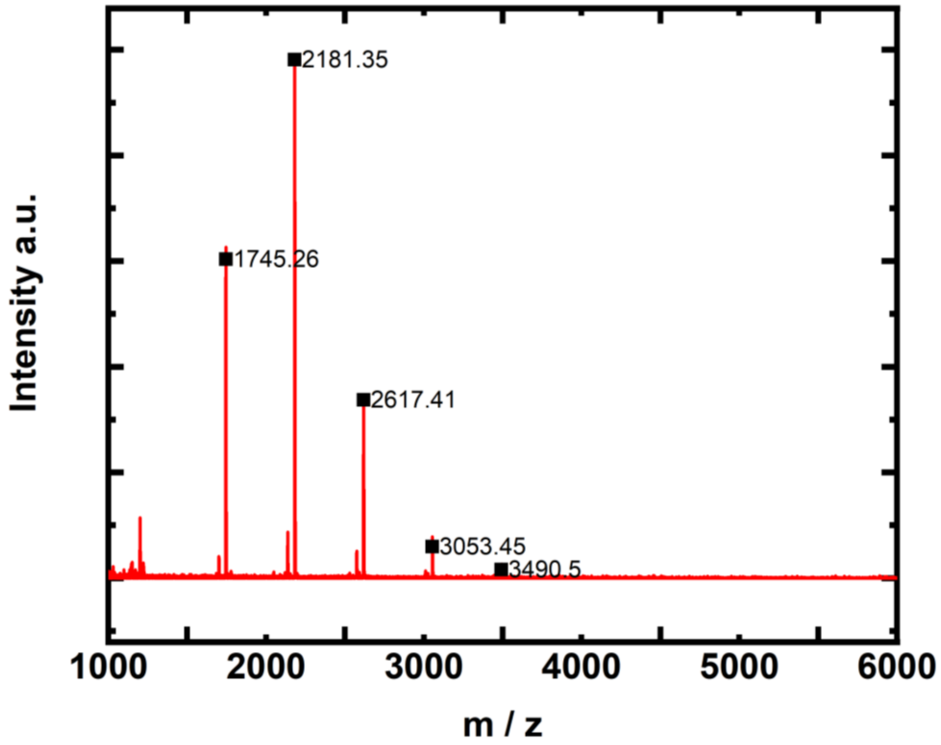

Figure S22. MALDI-TOF-Mass spectra of the filtrate from a reductive elimination of **2**, containing the masses of the individual ring sizes $\mathbf{1}_{[4\text{–}8]}$ marked as black squares.

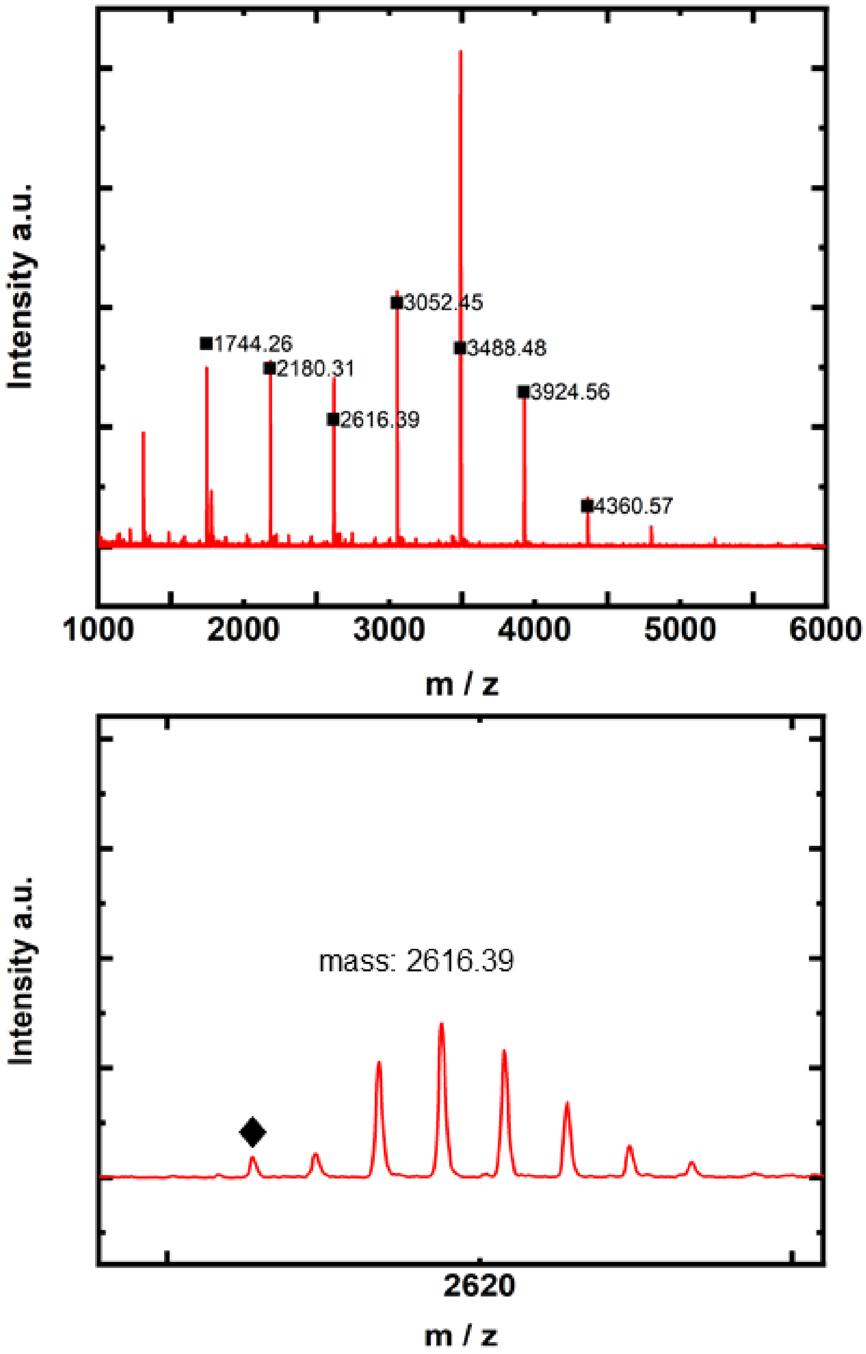

Figure S23. MALDI-TOF mass spectra of the solid precipitate isolated from a reductive elimination of **2**, containing the masses of the individual ring sizes $\mathbf{1}_{[4-8]}$ and linear oligomers (top). Enlarged display of the isotope pattern showing the mass of the smallest peak black rhomb (bottom). The isotope pattern shows a mix of linear and cyclic oligomer of $\mathbf{1}_{[6]}$.

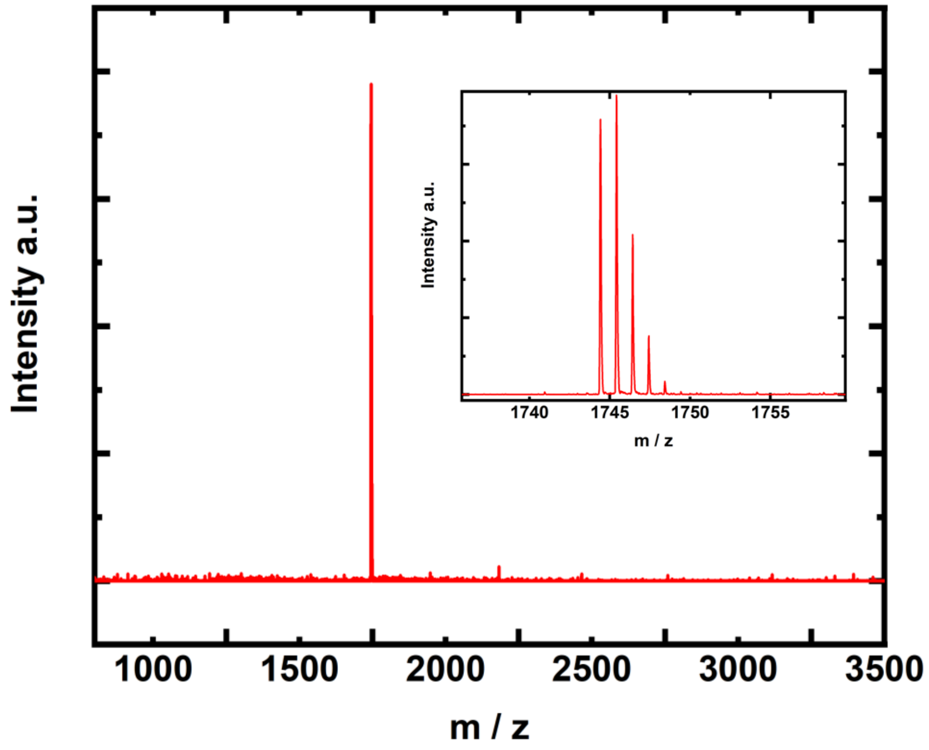

Figure S24. MALDI-TOF MS of $\mathbf{1}_{[4]}$.

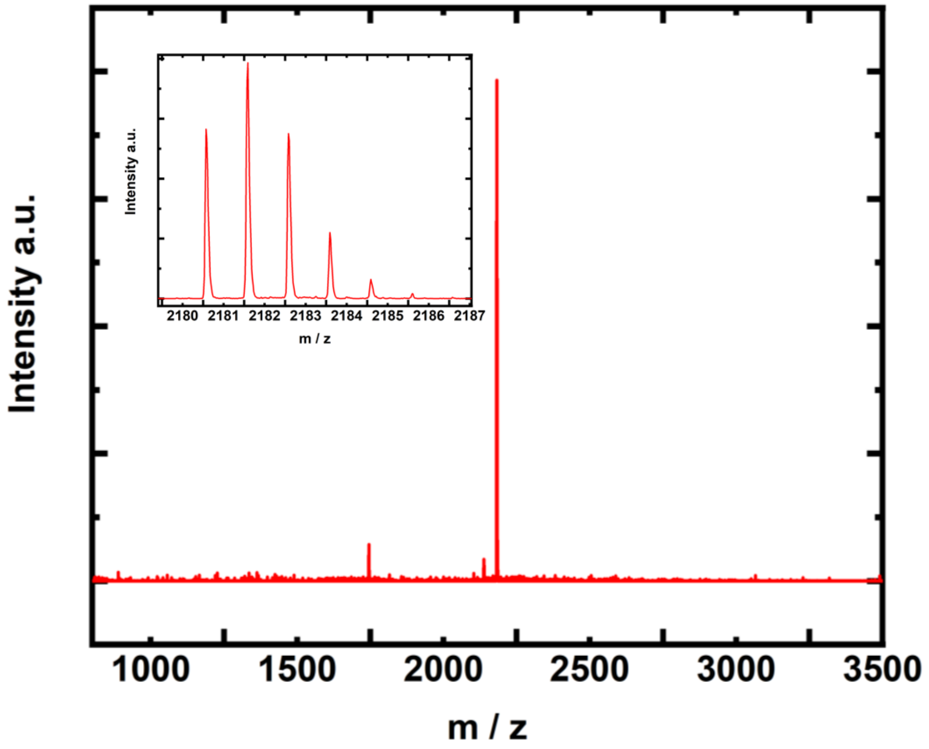

Figure S25. MALDI-TOF MS of $\mathbf{1}_{[5]}$.

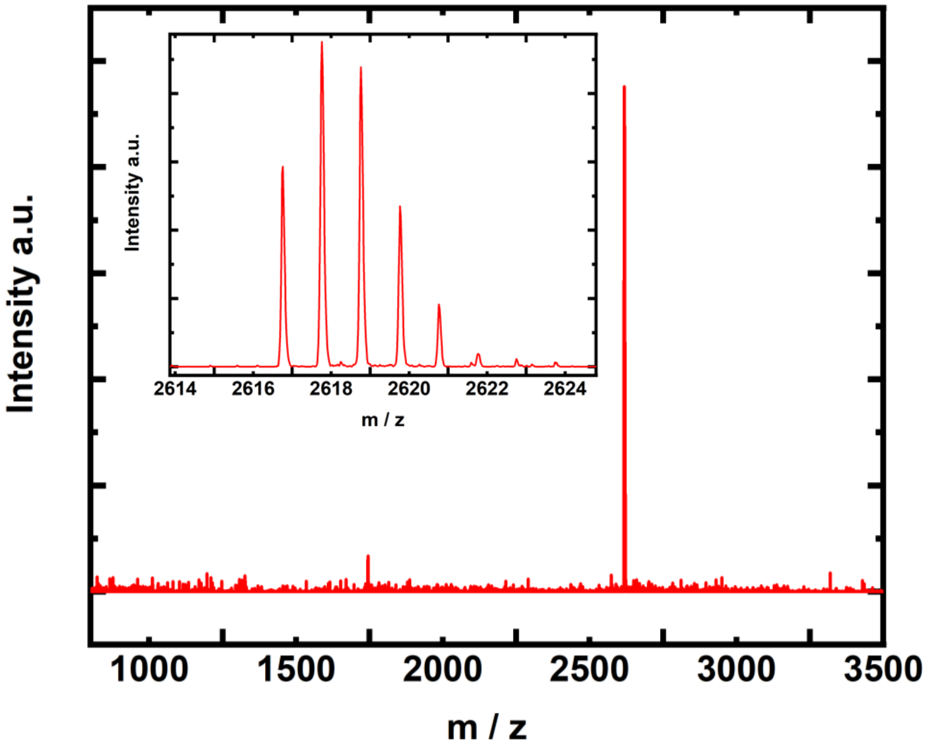

Figure S26. MALDI-TOF MS of **1**[6].

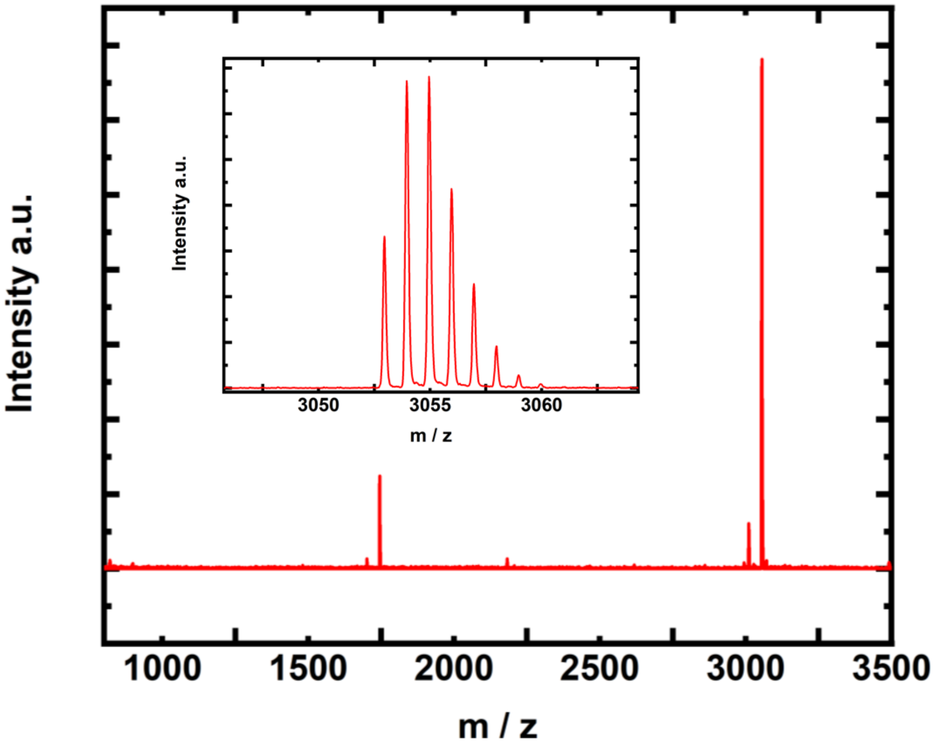

Figure S27. MALDI-TOF MS of **1**[7].

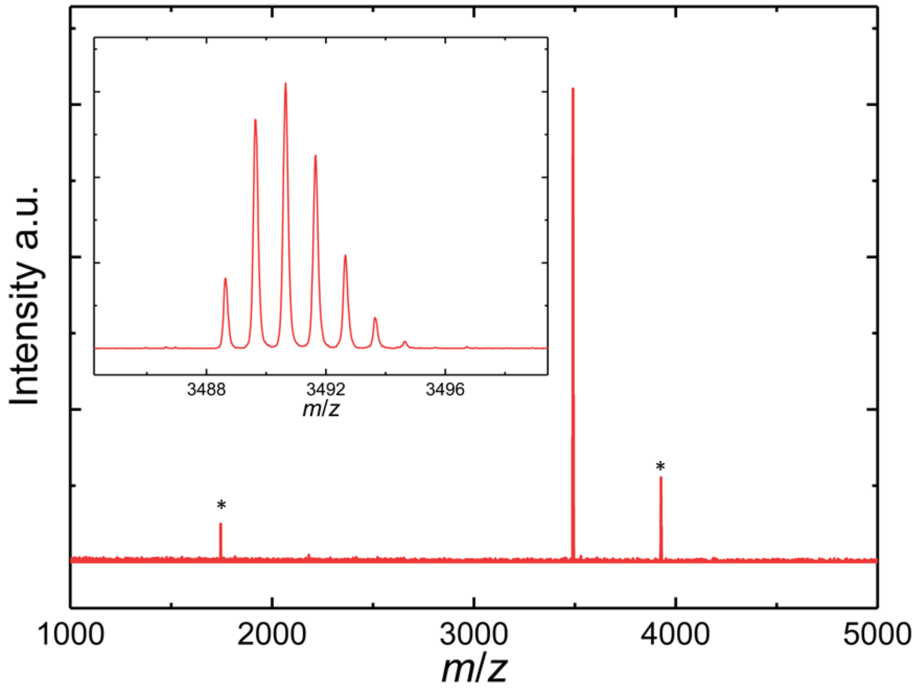

Figure S28. MALDI-TOF MS of **1**[8]. The stars mark the mass signal for **1**[4] and **1**[9].

S6. Single-Crystal X-ray Data

X-ray Crystallography. CCDC-1940871 (**5**), CCDC-2049516 (**4**), CCDC-2049515 (**3**), CCDC-2050148 (**1**[4]), CCDC-1876252 (**1**[6]), CCDC-2049763 (**1**[5]···18-crown-6·K^+ (BF_4^-)), contain the supplementary crystallographic data for this paper, including structure factors and refinement instructions. These data can be obtained free of charge from the joint Cambridge Crystallographic Data Centre and Fachinformationszentrum Karlsruhe Access Structures service (Cambridge Crystallographic Data Centre, 12 Union Road, Cambridge CB2 1EZ, UK (fax: +44(1223)-336-033; e-mail: deposit@ccdc.cam.ac.uk), or online via www.ccdc.cam.ac.uk/structures.

Single-crystal X-ray Data for Pyrene-4,5,9,10-tetrone (5)

Clear red plates were grown by recrystallization from hot $(CH_3)_2SO$ upon slow cooling.

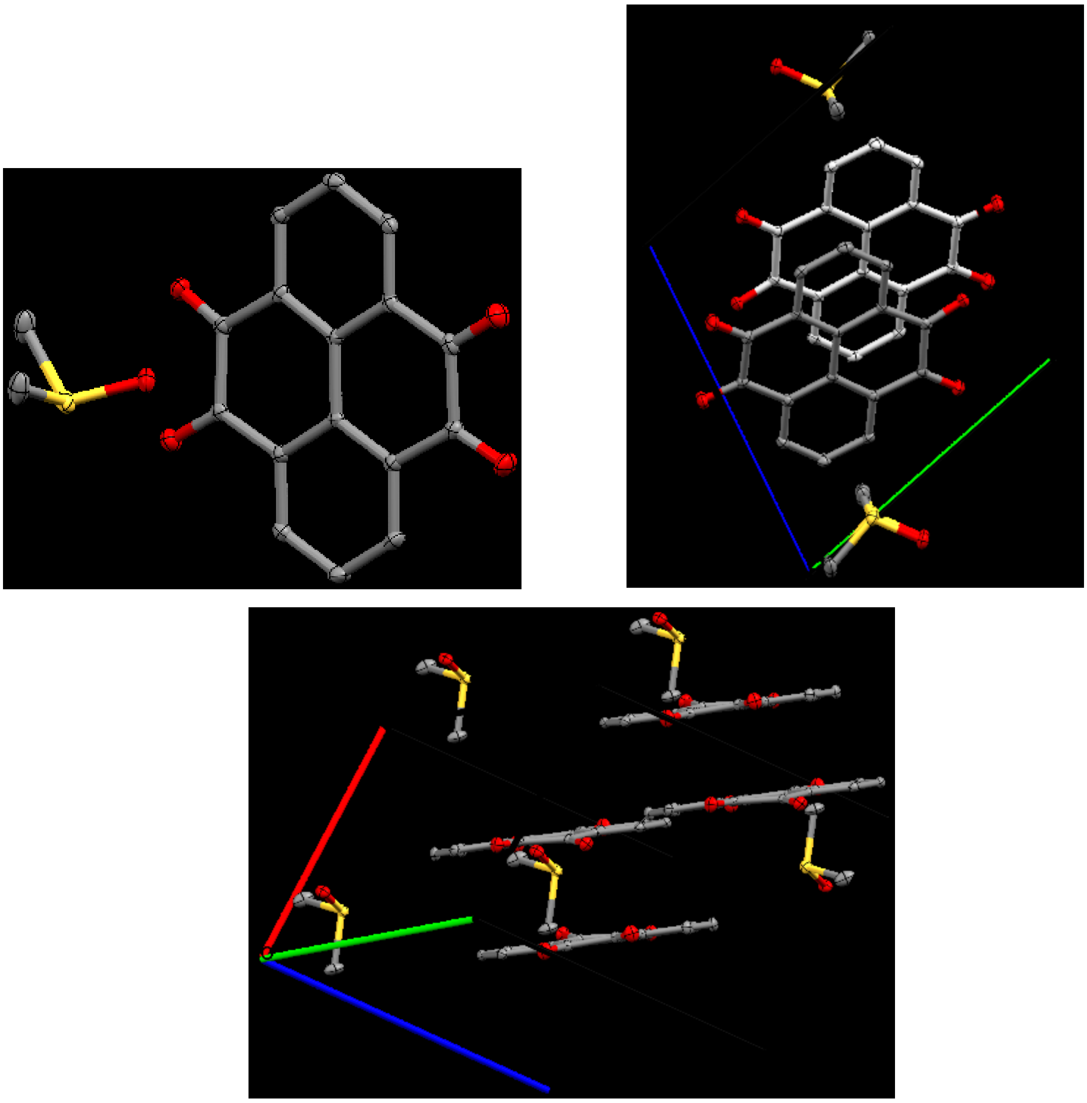

Figure S29. X-ray structure of **5** at 103 K. Ellipsoids are shown at 50% probability, hydrogen atoms are omitted for clarity. Color code: carbon, grey; oxygen, red; sulfur, yellow.

Table S2. Crystal data and structure refinement for **5**

CCDC Deposition number	1940871
Empirical formula	$C_{16}H_6O_4 \cdot (CH_3)_2SO$
Formula weight	340.34
Temperature/K	100.0
Crystal system	triclinic
Space group	P-1
a/Å	8.1346(6)
b/Å	9.4800(7)
c/Å	10.2701(7)
α/°	72.789(2)
β/°	83.483(2)
γ/°	72.502(2)
Volume/Å^3	721.26(9)
Z	2
ρ_{calc}g/cm^3	1.567
μ/mm^{-1}	0.252
F(000)	352.0
Crystal size/mm^3	1.036 × 0.24 × 0.234
Radiation	MoKα (λ = 0.71073)
2Θ range for data collection/°	4.688 to 56.712
Index ranges	$-10 \le h \le 10$, $-12 \le k \le 12$, $-13 \le l \le 13$
Reflections collected	24164
Independent reflections	3594 [R_{int} = 0.0773, R_{sigma} = 0.0496]
Data/restraints/parameters	3594/0/219
Goodness-of-fit on F^2	1.061
Final R indexes [$I \ge 2\sigma(I)$]	R_1 = 0.0504, wR_2 = 0.1114
Final R indexes [all data]	R_1 = 0.0732, wR_2 = 0.1219
Largest diff. peak/hole /e Å^{-3}	0.46/–0.44

Single-crystal X-ray Data for Pyrene-4,5,9,10-tetra(ethyleneglycol)ketal (4)

Clear colorless cubes were grown by slow evaporation from $CHCl_3$.

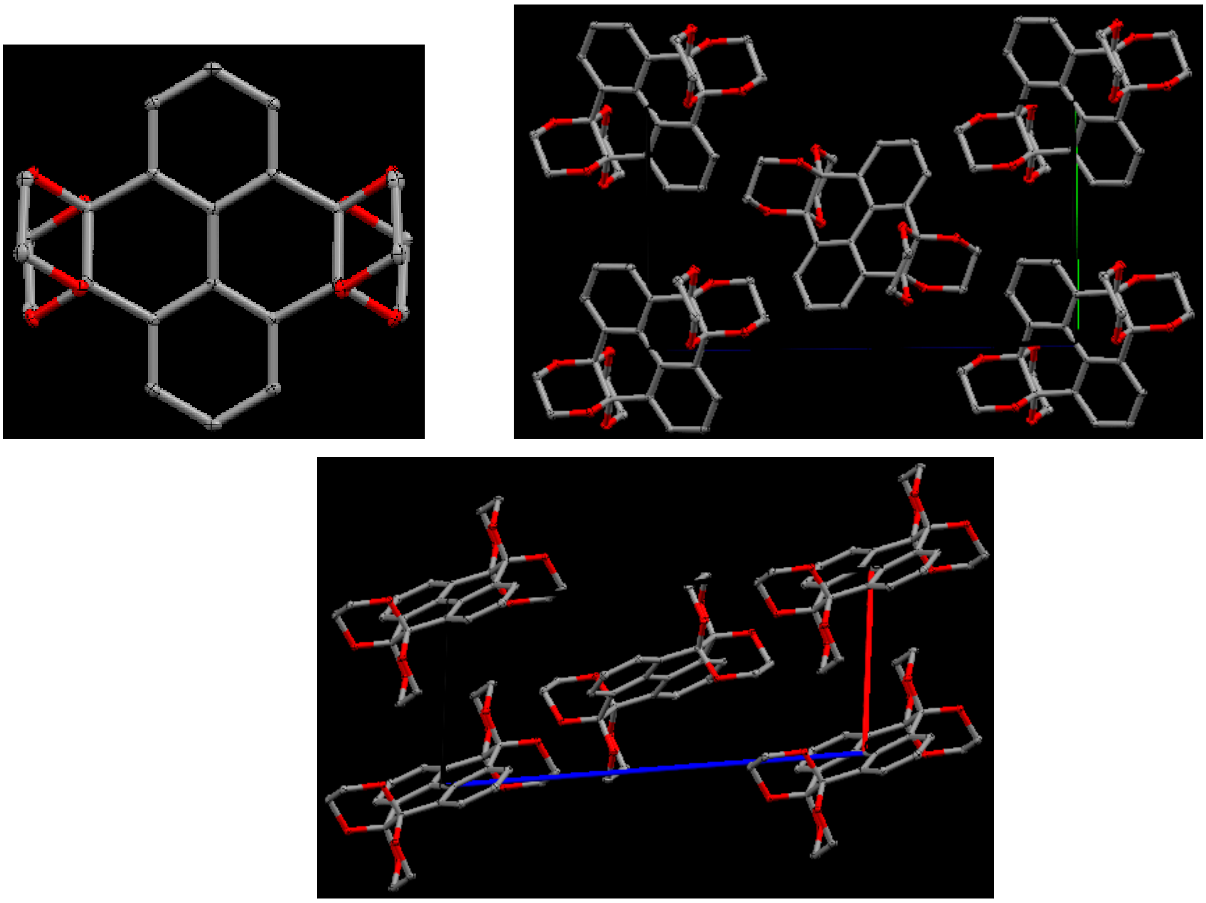

Figure S30. X-ray structure of **4** at 100 K. Ellipsoids are shown at 50% probability, hydrogen atoms are omitted for clarity. Color code: carbon, grey, oxygen, red.

Table S3. Crystal data and structure refinement for **4**.

CCDC Deposition number	2049516
Empirical formula	$C_{24}H_{22}O_8$
Formula weight	438.41
Temperature/K	100.01
Crystal system	monoclinic
Space group	$P2_1/n$
a/Å	6.866(3)
b/Å	8.750(3)
c/Å	15.594(6)
α/°	90
β/°	96.617(16)
γ/°	90
Volume/Å^3	930.6(6)
Z	2
ρ_{calc}g/cm^3	1.565
μ/mm^{-1}	0.118
F(000)	460.0

Crystal size/mm^3	0.13 × 0.1 × 0.08
Radiation	MoKα (λ = 0.71073)
2Θ range for data collection/°	5.26 to 61.186
Index ranges	$-9 \le h \le 9$, $-12 \le k \le 12$, $-22 \le l \le 22$
Reflections collected	34316
Independent reflections	2855 [R_{int} = 0.0719, R_{sigma} = 0.0306]
Data/restraints/parameters	2855/0/145
Goodness-of-fit on F^2	1.041
Final R indexes [$I \ge 2\sigma(I)$]	R_1 = 0.0411, wR_2 = 0.1059
Final R indexes [all data]	R_1 = 0.0496, wR_2 = 0.1110
Largest diff. peak/hole /e Å^{-3}	0.56/–0.28

Single-crystal X-ray Data for 2,7-Bis-pinacolatoboron-pyren-4,5,9,10-tetra(ethyleneglycol)ketal (3)

Clear colorless blocks were grown via slow evaporation from a $CDCl_3$ solution.

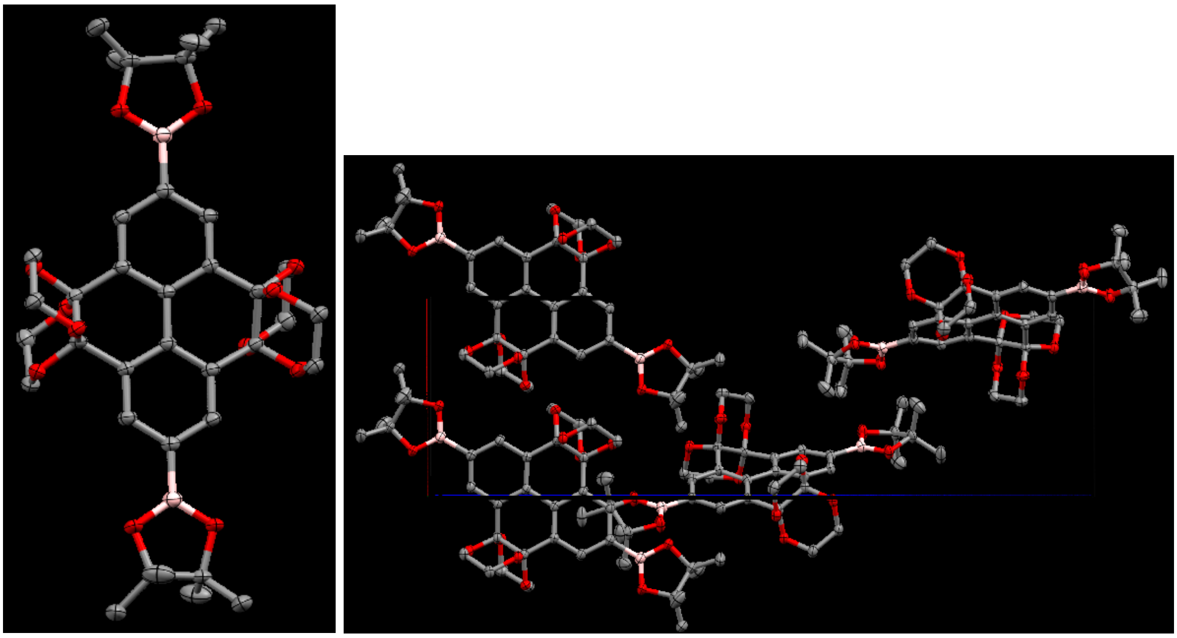

Figure S31. X-ray structure of **3** at 100 K. Ellipsoids are shown at 50% probability, hydrogen atoms are omitted for clarity. Color code: carbon, grey; oxygen, red; boron, rosé.

Table S4. Crystal data and structure refinement for **3**.

CCDC Deposition number	2049515
Empirical formula	$C_{36}H_{44}B_2O_{12}$
Formula weight	690.33
Temperature/K	100.15
Crystal system	trigonal
Space group	$P3_121$
a/Å	10.1130(8)
b/Å	10.1130(8)
c/Å	28.979(2)
α/°	90
β/°	90
γ/°	120

Volume/Å^3	2566.7(4)
Z	3
ρ_{calc}g/cm^3	1.340
μ/mm^{-1}	0.818
F(000)	1098.0
Crystal size/mm^3	0.1 × 0.1 × 0.1
Radiation	CuKα (λ = 1.54178)
2Θ range for data collection/°	10.1 to 144.992
Index ranges	$-12 \leq h \leq 12, -12 \leq k \leq 12, -35 \leq l \leq 35$
Reflections collected	52326
Independent reflections	3399 [R_{int} = 0.1063, R_{sigma} = 0.0333]
Data/restraints/parameters	3399/0/231
Goodness-of-fit on F^2	1.019
Final R indexes [$I \geq 2\sigma (I)$]	R_1 = 0.0439, wR_2 = 0.1123
Final R indexes [all data]	R_1 = 0.0515, wR_2 = 0.1174
Largest diff. peak/hole /e Å^{-3}	0.86/–0.20

Remark on X-ray Crystal Structure Determination of Macrocycles

Generally, single crystals of macrocycles are difficult to grow due to limited solubility, and they must be handled, picked, and mounted carefully to prevent solvent loss. The resulting statistical indicators cannot be compared to results typically obtained for small molecule structures due to several problems (e.g. disordered solvent, low resolution). Even though some solvent molecules could be refined, the solvent mask tool implemented in OLEX2 was used for the following three structures.[6]

S6.1 Single-crystal X-ray Data and Structure analysis for **1**[4]

Yellow cuboids were grown via layer diffusion of MeOH into a $CDCl_3$ solution of the compound.

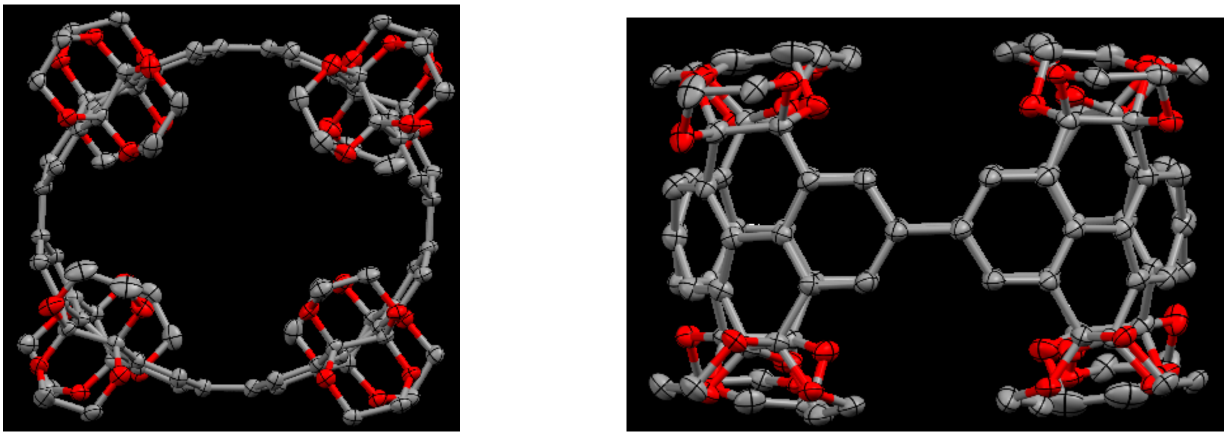

Figure S32. X-ray structure of **1**[4] at 100 K. Ellipsoids are shown at 50% probability, hydrogen atoms and solvent molecules are omitted for clarity. Color code: carbon, grey; oxygen; red.

Table S5. Crystal data and structure refinement for **1**[4].

CCDC Deposition number	2050148
Empirical formula	$C_{96}H_{78}O_{32}$
Formula weight	1743.58
Temperature/K	100.0
Crystal system	triclinic
Space group	*P*-1
a/Å	14.1927(11)
b/Å	14.2123(13)
c/Å	15.9989(14)
α/°	103.089(3)
β/°	112.535(3)
γ/°	100.195(3)
Volume/Å^3	2775.7(4)
Z	1
ρ_{calc}g/cm^3	1.043
μ/mm^{-1}	0.079
F(000)	910.0
Crystal size/mm^3	0.17 × 0.126 × 0.124
Radiation	MoKα (λ = 0.71073)
2Θ range for data collection/°	3.764 to 50.92
Index ranges	$-17 \leq h \leq 17$, $-17 \leq k \leq 17$, $-19 \leq l \leq 19$
Reflections collected	108475
Independent reflections	10206 [R_{int} = 0.0562, R_{sigma} = 0.0253]
Data/restraints/parameters	10206/0/577
Goodness-of-fit on F^2	1.114
Final *R* indexes [$I \geq 2\sigma (I)$]	R_1 = 0.0655, wR_2 = 0.1743
Final *R* indexes [all data]	R_1 = 0.0822, wR_2 = 0.1852
Largest diff. peak/hole /e Å^{-3}	0.57/–0.44

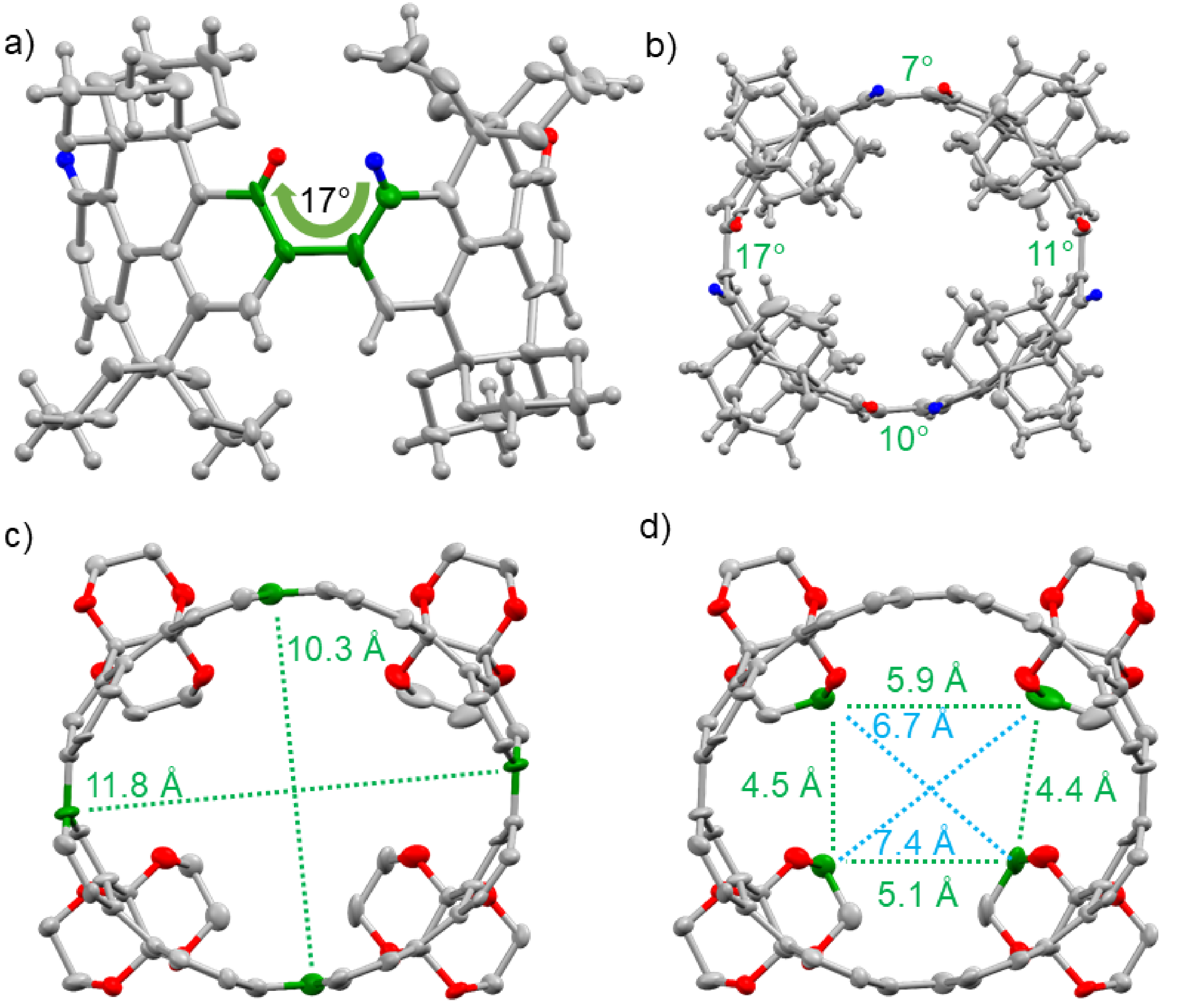

Figure S33. a) Exemplary representation of dihedral angle measurement (green); b) dihedral angles (green) between the different pyrene units, according H atoms pointing into the ring center are marked red and out of the ring center are marked blue; c) short and long diameter in the central plane of the molecule between the respective carbon atoms (green); d) heavy atom distances between the carbons atoms (green) of the protecting groups marking the opening of the internal molecular cavity.

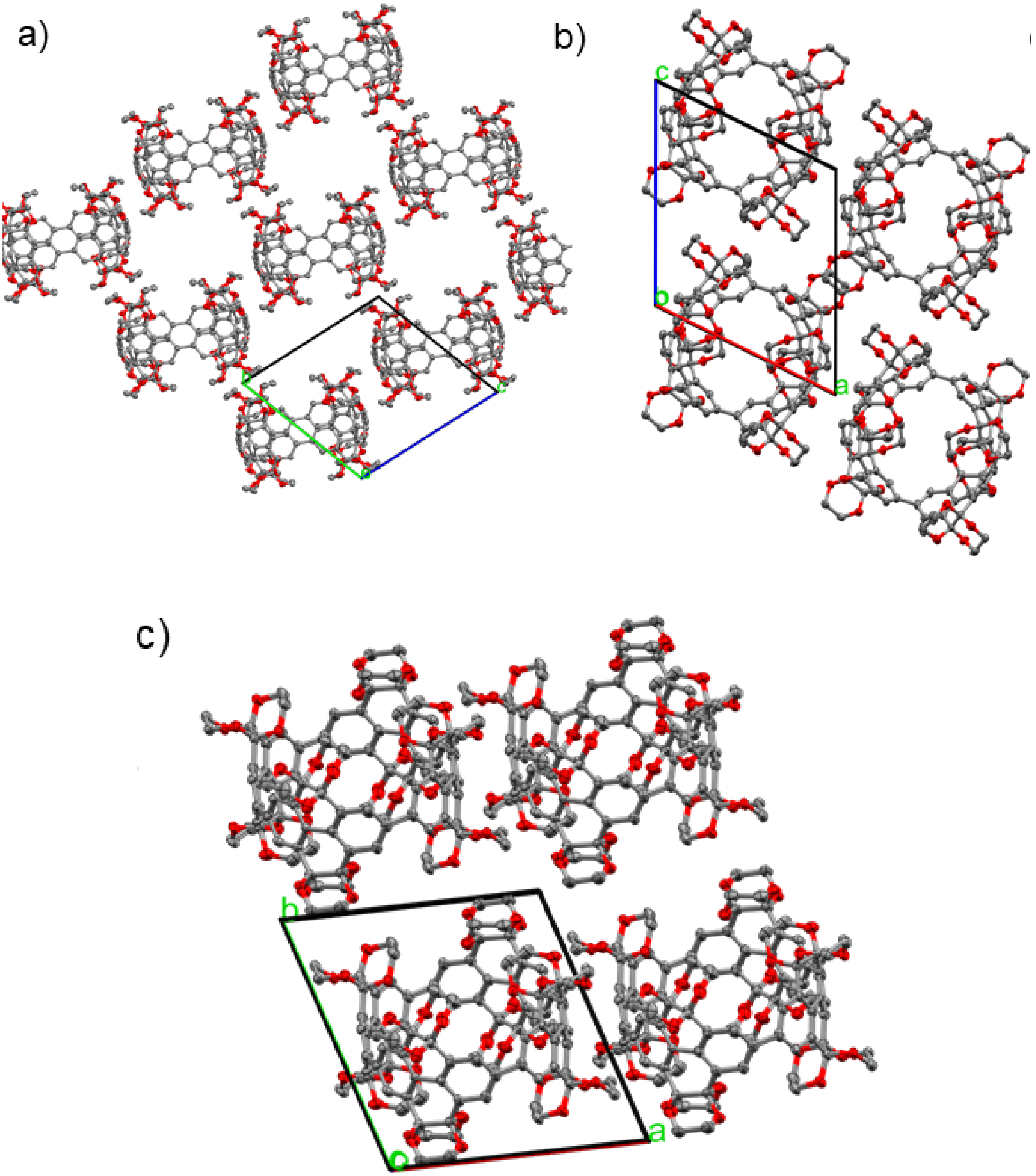

Figure S34. Single crystal X-ray packing of **1**[4] a) along the *a*-axis, b) along the *b*-axis, c) along the *c*-axis.

S6.2 Single-crystal X-ray Data and Structure analysis for **1**[6]

Clear plates were grown via slow evaporation from a $CHCl_3$ solution.

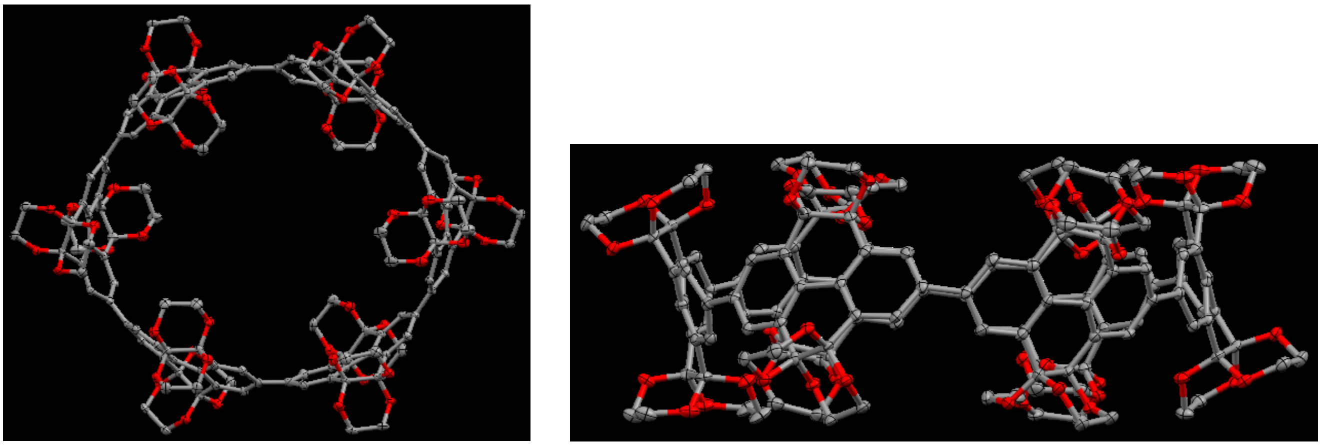

Figure S35. X-ray structure of **1**[6] at 100 K. Ellipsoids are shown at 50% probability, hydrogen atoms and solvent molecules are omitted for clarity. Color code: carbon, grey; oxygen, red.

Table S6. Crystal data and structure refinement for **1**[6].

CCDC Deposition number	1876252
Empirical formula	$C_{144}H_{120}O_{48} \cdot 18\ CHCl_3$
Formula weight	4767.02
Temperature/K	100.15
Crystal system	triclinic
Space group	*P*-1
a/Å	14.5936(12)
b/Å	18.1558(15)
c/Å	20.4131(18)
α/°	93.925(3)
β/°	99.896(3)
γ/°	103.790(3)
Volume/Å^3	5140.6(8)
Z	1
ρ_{calc}g/cm^3	1.540
μ/mm^{-1}	0.780
F(000)	2412.0
Crystal size/mm^3	0.1 × 0.1 × 0.1
Radiation	MoKα (λ = 0.71073)
2Θ range for data collection/°	3.88 to 51.54
Index ranges	$-17 \le h \le 17$, $-22 \le k \le 22$, $-24 \le l \le 24$
Reflections collected	207617
Independent reflections	19611 [R_{int} = 0.1092, R_{sigma} = 0.0494]
Data/restraints/parameters	19611/0/1189
Goodness-of-fit on F^2	1.032
Final R indexes [$I \ge 2\sigma (I)$]	R_1 = 0.0858, wR_2 = 0.2253
Final R indexes [all data]	R_1 = 0.1231, wR_2 = 0.2546
Largest diff. peak/hole /e Å^{-3}	2.12/–1.45

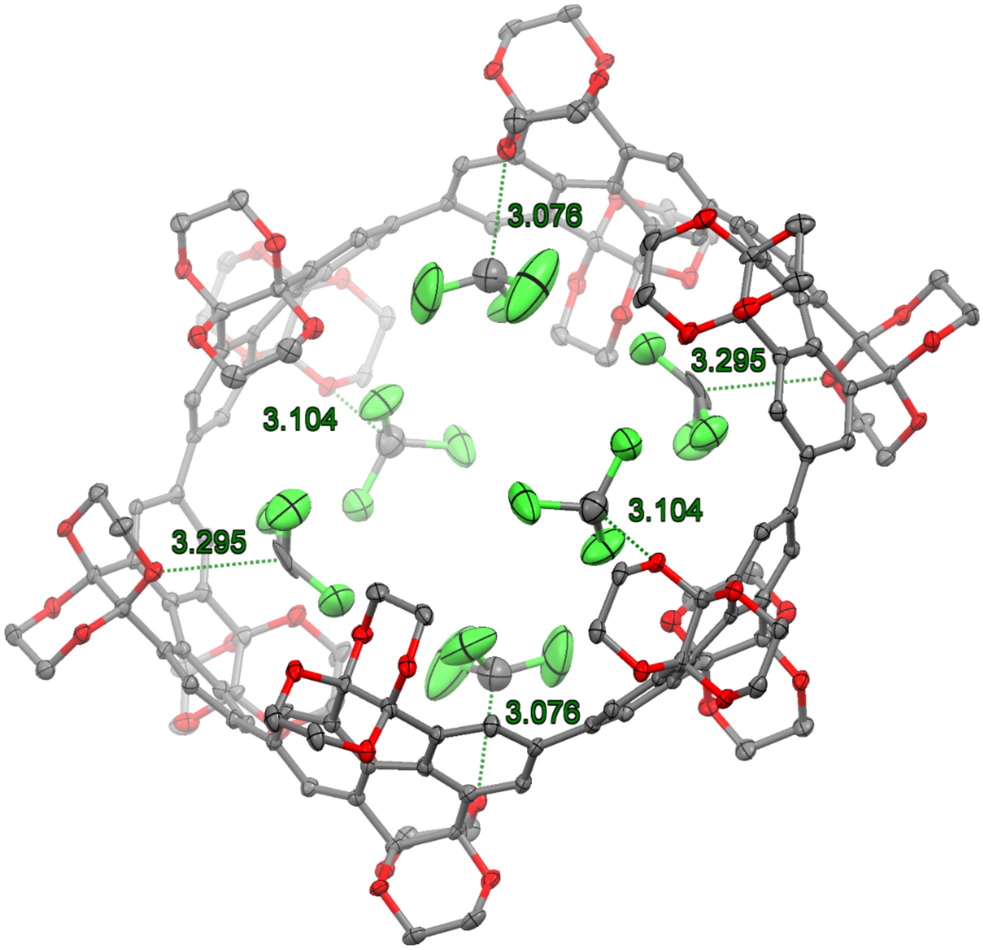

FigureS36. Singe-crystal X-ray structures of **1**$_{[6]}$ and six chloroform molecules within the cavity. The green dashed lines show the heavy atom distance between the chloroform carbon and the ethylene glycol oxygen demonstrating C–H···O hydrogen bonding. Distances in Å.

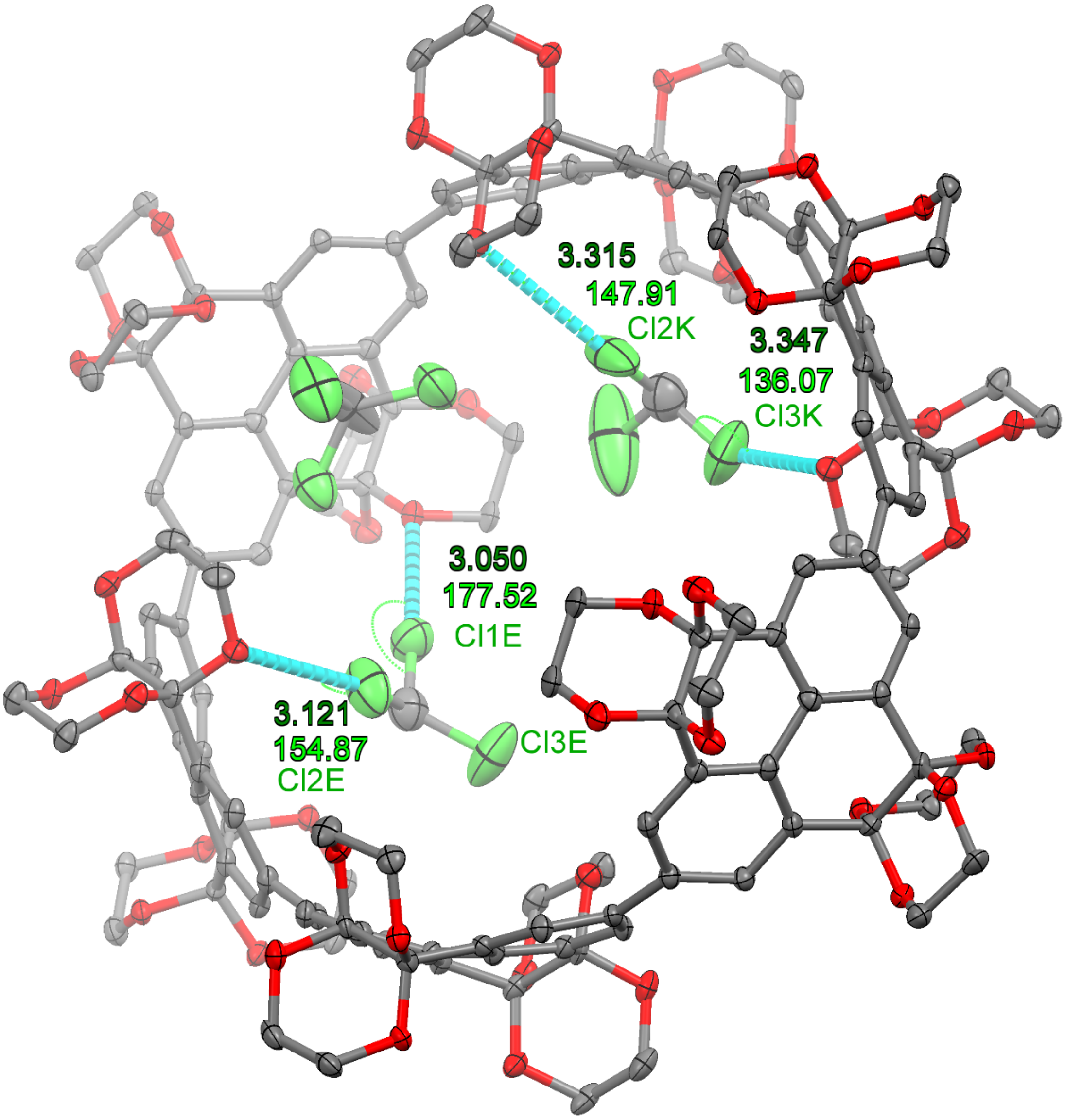

Figure S37. Singe-crystal X-ray structures of **1**[6] and three chloroform molecules inside the cavity. The light blue dashed lines show the heavy atom distance between the chloroform chlorine and the ethylene glycol oxygen demonstrating C–Cl··· O halogen bonding. Distances (dark green) in Å, angles (light green) in degree (top).

XB inside the cavity:

1. Angle(O···Cl—C) = 178°, *d*(O···Cl) = 3.05 Å, **7%** below the sum of the vdW radii (at chlorine 1E).

additional weak XB:
2. angle(O···Cl—C) = 154°, *d*(O···Cl) = 3.12 Å, **5%** below the sum of the vdW radii (at chlorine 2E).
3. angle(O···Cl—C) = 148°, *d*(O···Cl) = 3.32 Å, **4%** below the sum of the vdW radii (at chlorine 2K).
4. angle(O···Cl—C) = 136°, *d*(O···Cl) = 3.35 Å, **3%** below the sum of the vdW radii (at chlorine 3K).

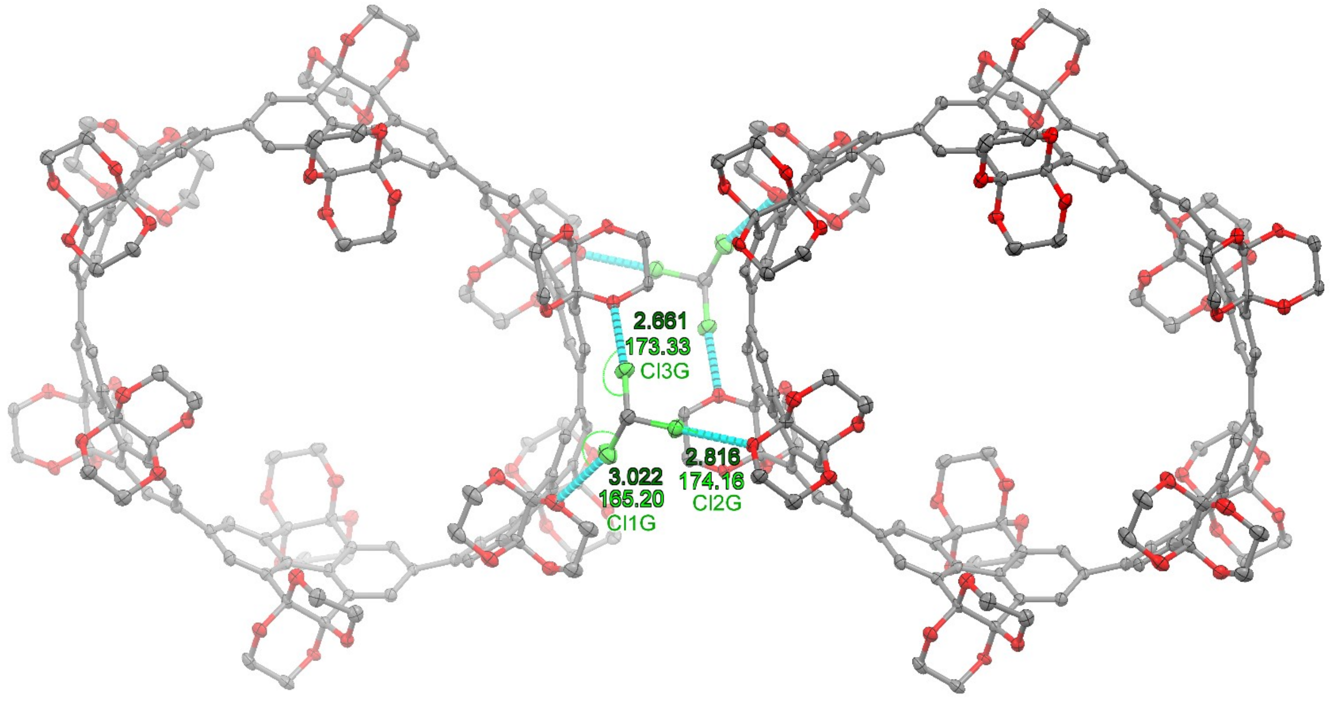

Figure S38. Singe-crystal X-ray structures of two molecules of **1**$_{[6]}$ interconnected by two chloroform molecules. Each chlorine is part of a C–Cl···O halogen bond shown with the light blue dashed lines (angles C–Cl···O in ° (light green), $d_{Cl\cdots O}$ in Å (dark green)).

Ring-to-ring side-on packing along one dimension of **1**$_{[6]}$ is dominated by an impressive network of six halogen bonds, where two chloroform solvates contribute with all six chlorine atoms to very short Cl···O distances with down to –19% of the sum of the vdW radii (*r*(Cl) + *r*(O)= 327 pm) and nearly linear angles O···Cl–C of 165°–174°. Additional halogen bonds are found inside the cavity, where chloroform solvates gain structural resolution through a combination of O···Cl (178°, –7% vdWr) interactions and O···H–CCl3 hydrogen bonds.

Ring-to-ring $CHCl_3$-mediated XB:

5. angle(O···Cl—C) = 173°, *d*(O···Cl) = 2.66 Å, **19%** below the sum of the vdW radii (at chlorine 3G).
6. angle(O···Cl—C) = 174°, *d*(O···Cl) = 2.82 Å, **14%** below the sum of the vdW radii (at chlorine 2G).
7. angle(O···Cl—C) = 165°, *d*(O···Cl) = 3.02 Å, **8%** below the sum of the vdW radii (at chlorine 1G)

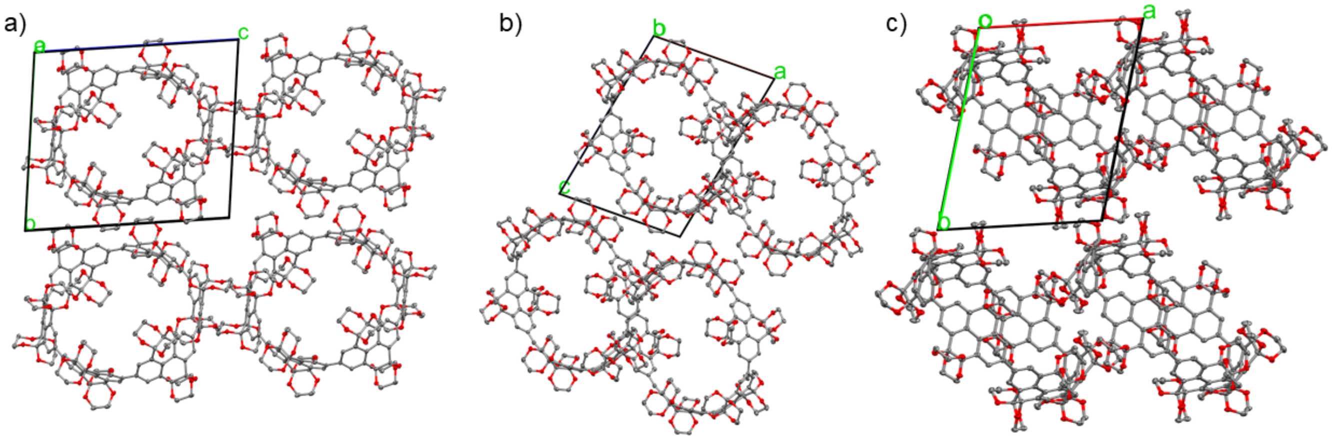

Figure S39. Single crystal X-ray packing of **1**[6] a) along the *a*-axis, b) along the *b*-axis, c) along the *c*-axis.

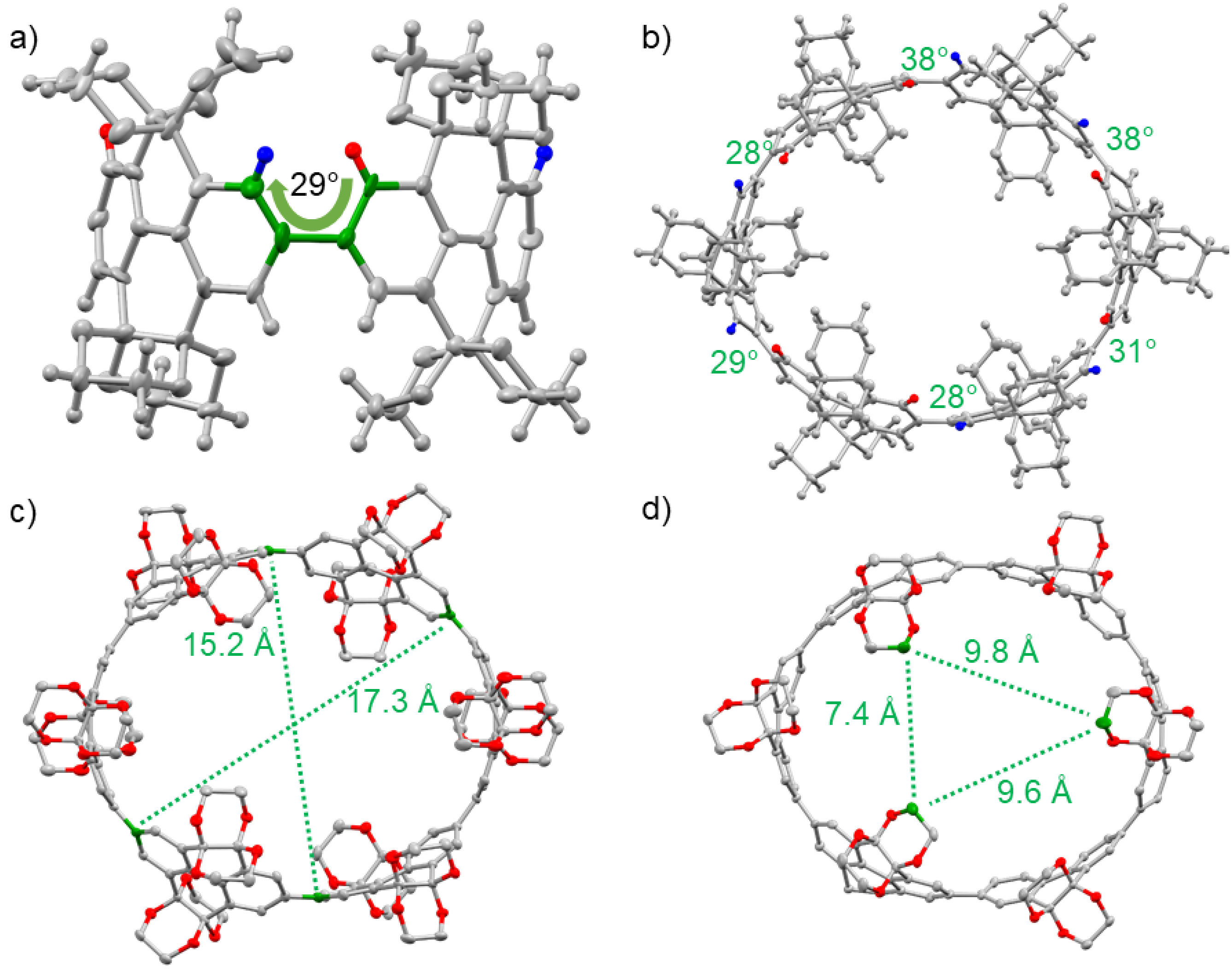

Figure S40. a) Exemplary representation of dihedral angle is measurements (green); b) dihedral angles (green) between the different pyrene units, according H atoms pointing into the ring center are marked red and out of the ring center are marked blue; c) short and long diameter in the central plane of the

molecule between the respective carbon atoms (green); d) heavy atom distances between the carbons atoms (green) of the ethylene glycol protecting groups marking the opening of the internal molecular cavity.

S6.3 Single-crystal X-ray Data and Structure analysis for 1[5]···18-crown-6·K(BF4)

Golden rods were grown via layer diffusion of MeOH into a $CHCl_3$ solution of **1**[5] and an excess of 18-crown-6 as well as KBF_4 at 23 °C. The data was collected at the MX 14.2 Beamline of the BESSYII synchrotron at the Helmholtz-Zentrum Berlin. The low completeness is due to lack of full angular freedom of the sample holder (missing four-circle kappa goniometer) at this beamline. A full data collection on this triclinic (*P*-1) crystal was therefore not obtained. Reflections of multiple crystals were collected, each in different orientation, and gave the same result after refinement each. The large displacement parameters are due to the disorder of the $CHCl_3$ solvent molecules and the BF_4^- anion that could not be improved further. Despite several attempts in other solvents (DMSO, 1,2-dichlorobenzene) and different techniques (vapor diffusion, slow evaporation) higher quality crystals could not be grown.

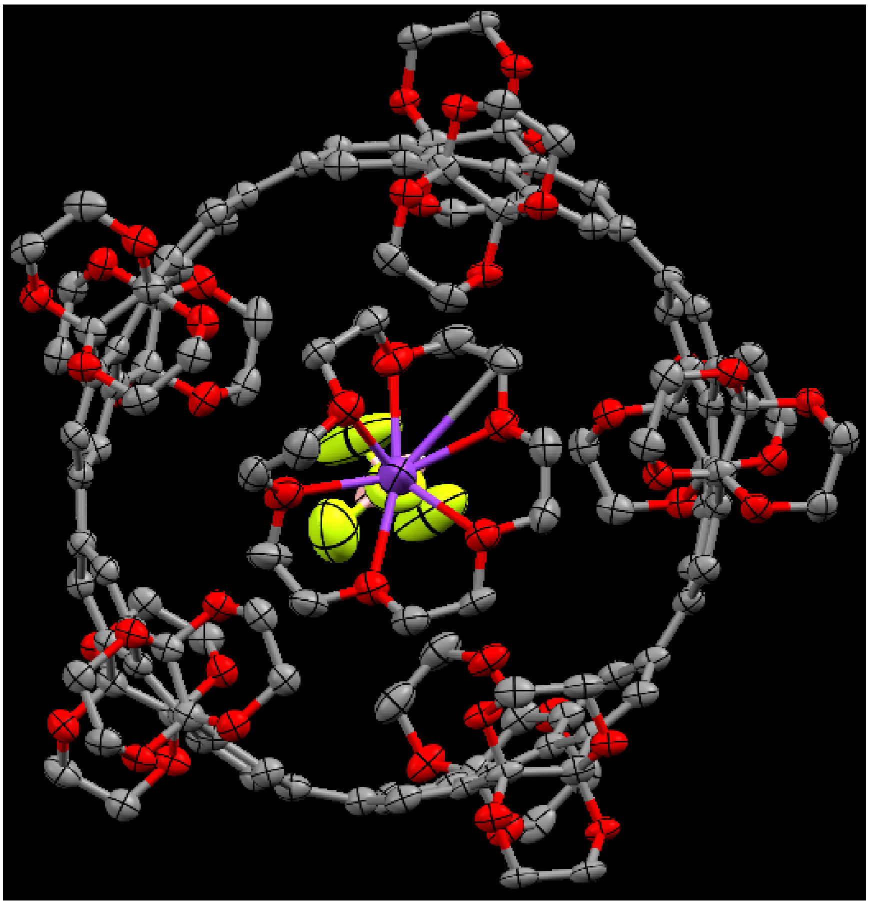

Figure S41. X-ray structure of **1**[5]···18-crown-6·K(BF_4) at 100 K. Ellipsoids are shown at 50% probability, hydrogen atoms, solvent molecules, and disorder are omitted for clarity. Color code: carbon, grey; oxygen, red; potassium, purple; fluorine, yellow; boron, rosé.

Table S7. Crystal data and structure refinement for **1**[5]···18-crown-6·K(BF_4)

CCDC Deposition number	2049763

Empirical formula	$C_{132}H_{124}BF_4KO_{46} \cdot 4\ CHCl_3$
Formula weight	3049.69
Temperature/K	100
Crystal system	triclinic
Space group	*P*-1
a/Å	11.020(2)
b/Å	19.153(4)
c/Å	31.999(6)
α/°	87.92(3)
β/°	81.74(3)
γ/°	82.00(3)
Volume/Å^3	6618(2)
Z	2
ρ_{calc}g/cm^3	1.530
μ/mm^{-1}	0.379
F(000)	3152.0
Crystal size/mm^3	0.1 × 0.1 × 0.1
Radiation	Synchrotron (λ = 0.82656)
2Θ range for data collection/°	1.286 to 48.814
Index ranges	$-12 \leq h \leq 12$, $-20 \leq k \leq 20$, $-37 \leq l \leq 37$
Reflections collected	70246
Independent reflections	19999 [R_{int} = 0.0662, R_{sigma} = 0.0591]
Data/restraints/parameters	19999/224/1847
Goodness-of-fit on F^2	1.560
Final *R* indexes [$I \geq 2\sigma (I)$]	R_1 = 0.1283, wR_2 = 0.3787
Final *R* indexes [all data]	R_1 = 0.1687, wR_2 = 0.4098
Largest diff. peak/hole /e Å^{-3}	1.59/–1.17

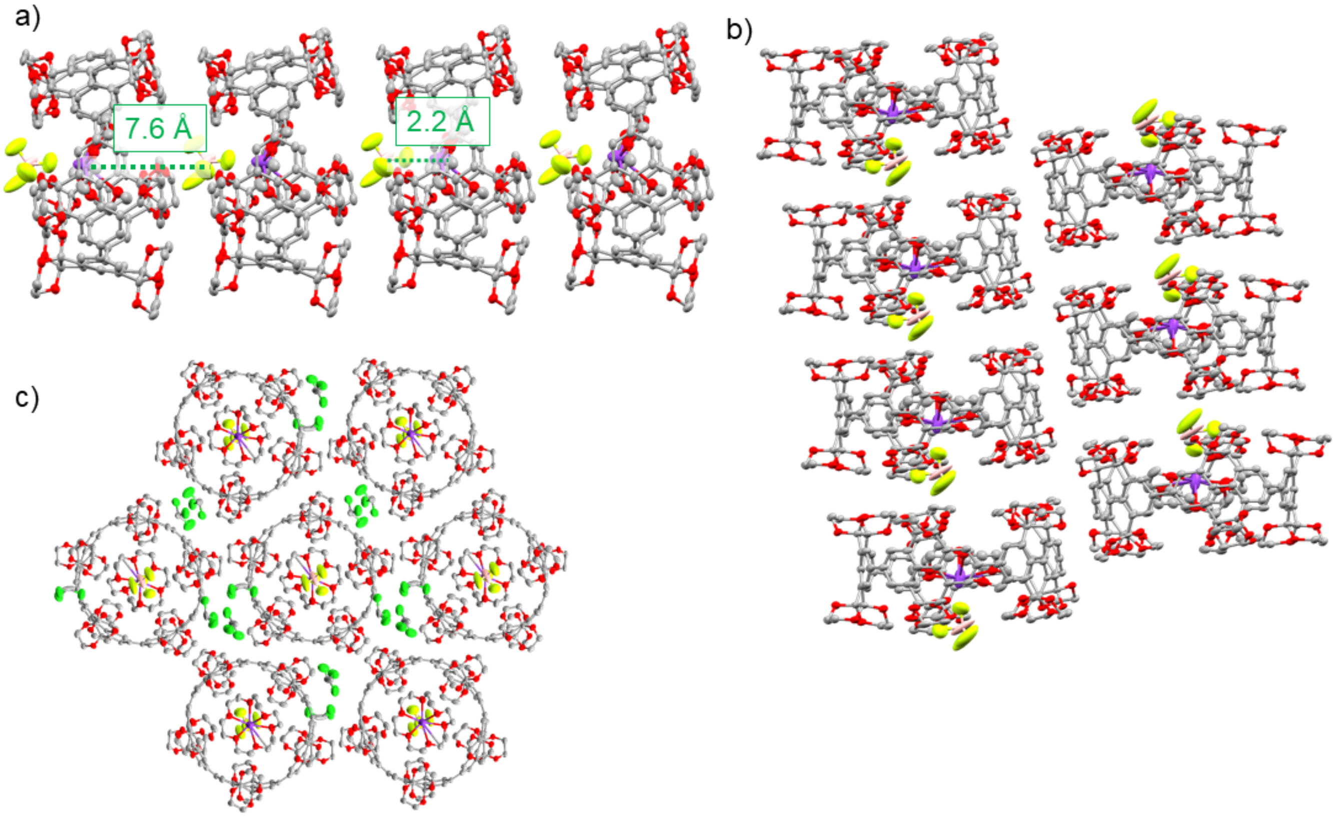

Figure S42. a) Columnar packing of **1**$_{[5]}$···18-crown-6·K(BF_4) without solvent, showing the distances between the K^+ cation and the BF_4^- anion; b) anti-parallel packing of two neighboring columns; c) cross-section through columnar packed **1**$_{[5]}$···18-crown-6·K(BF_4) with solvent molecules.

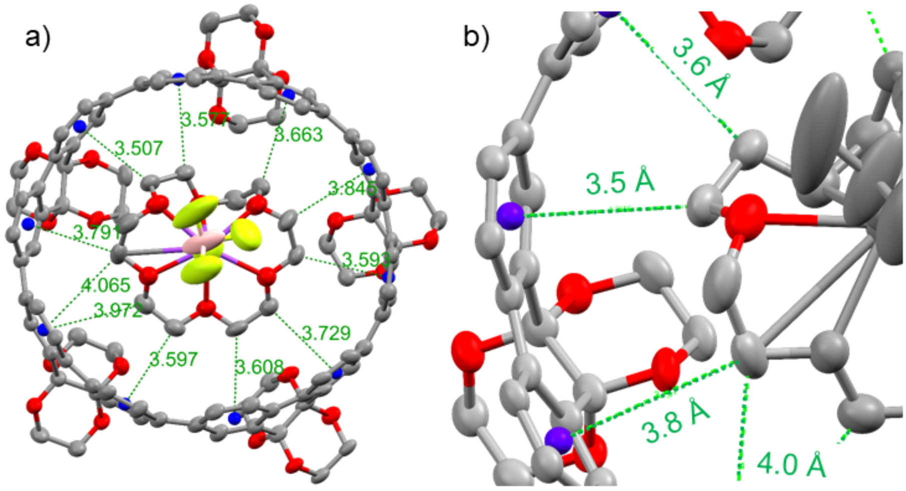

c)

CH-π	Distance [Å]	Ø [Å]
1	3.5	
2	3.6	
3	3.7	
4	3.8	
5	3.6	3.7
6	3.7	
7	3.6	
8	3.6	
9	4.0	
10	3.8	

Figure S43. a) Heavy atom distances (in Å) between the blue centroids places in the middle of the individual phenyl rings and the closest carbon atoms of the 18-crown-6 ether moiety; b) enlarged representation of the interaction; c) table showing all heavy atom distances between the constructed centroids of the regarding phenyl rings and the carbon atoms of the 18-crown-6 ether.

For the following representation of hydrogen bonds in the crystal structure between host and guest, the guest is colored and divided into three different sections (Figure S44b).

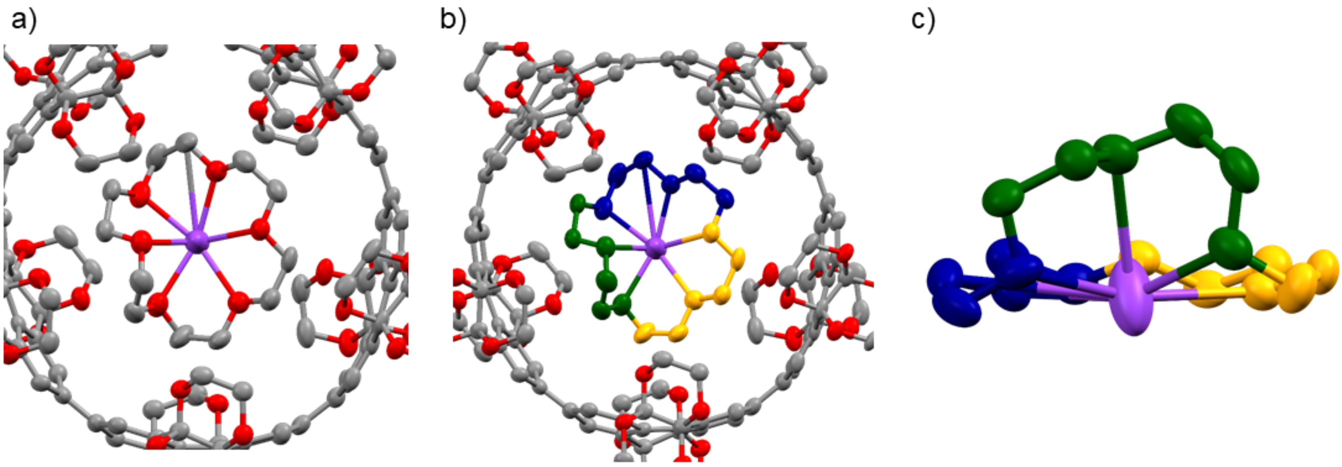

Figure S44. a) Enlarged view on the 18-crown-6 ether·K^{+} inside the cavity, b) coloring of the guest resulting in three parts, c) enlarged view on the colored guest.

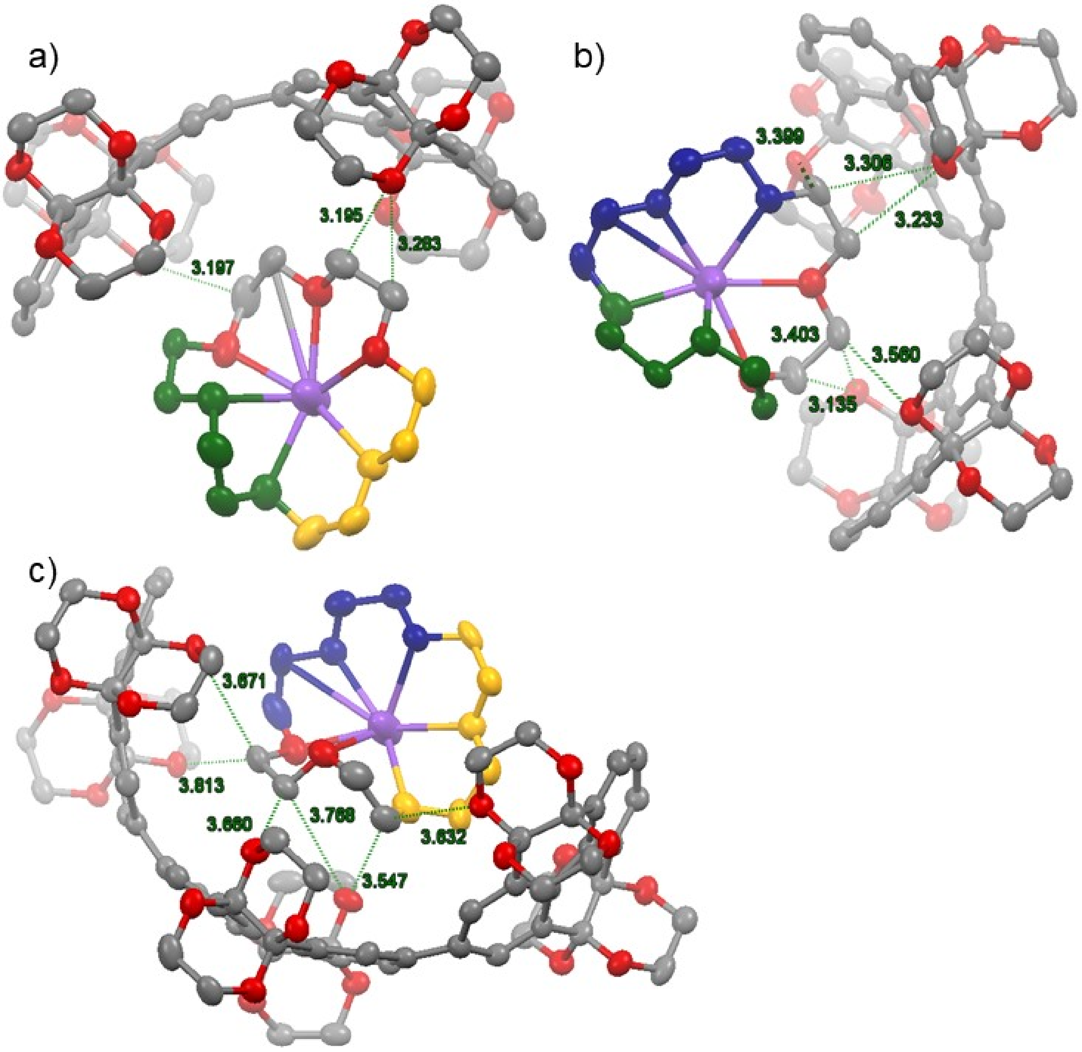

Figure S45. a) Close contacts (in Å) between the blue part of the guest and the host; b) close contacts (in Å) between the blue part of the guest and the host; c) close contacts (in Å) between the blue part of the guest and the host.

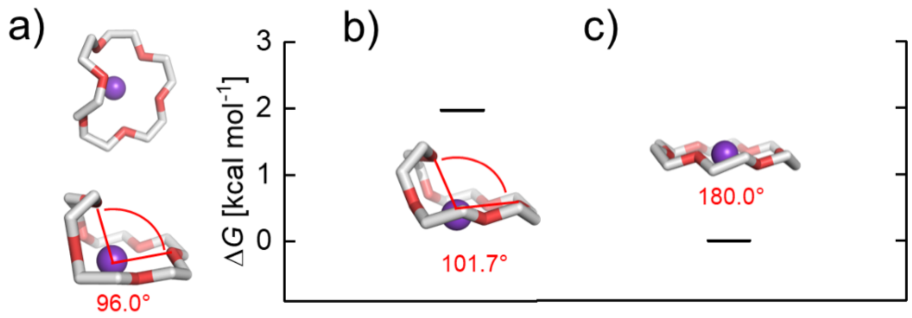

Figure S46. a)Top and side view of 18-crown-6 ether as extracted from the X-ray structure of the **1**$_{[5]}$···18-crown-6·K(BF_4) complex, **b)** geometry optimization of the X-ray structure with constraints dihedral angles of the bend crown ether half, at the B3LYP-D3/def2QZVP level of theory **c)** fully relaxed geometry-optimized structure of 18-crown-6 ether in its the planar conformation without any constrains, at the B3LYP-D3/def2QZVP level of theory. Angles O–M–O of opposite O-ether fragments are depicted in red. The relative calculated Gibbs energies of b) and c) are plotted. Structure b) is 2.0 kcal mol^{-1} higher in energy than structure c).

S7. Geometrical Considerations and Volume Analysis of the Hosts [*n*]Cyclo-pyrenylenes 1$_{[4–8]}$

The following section shows single crystal X-ray and geometry optimized structures (B3LYP-D3/def2SVP) of **1**$_{[4–8]}$ and the heavy atom distances, which define the diameter (d_{gate}) of the opening and the diameter of the cavity (d_{cavity}) in the corresponding macrocycle. Eventually, the average is used to calculate the ratio between the two parameters.

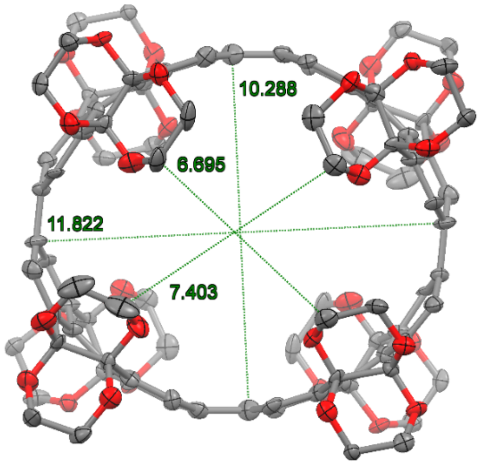

Figure S47. Single crystal X-ray structure of **1**$_{[4]}$, the dashed green lines mark the heavy atom distance between the C atoms of d_{cavity} (11.822 Å, 10.288 Å), and the heavy atom distance between the C atoms of d_{gate} (6.695 Å, 7.403 Å).

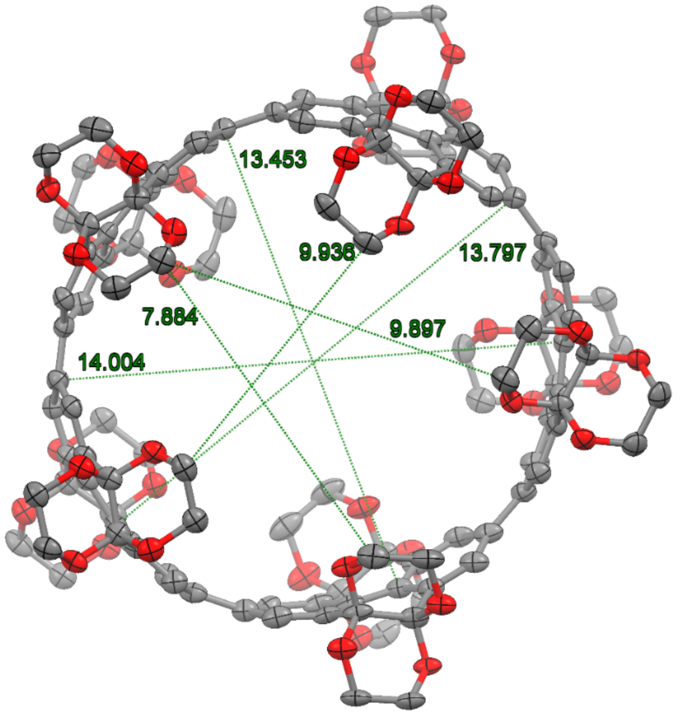

Figure S48. Single crystal X-ray structure of **1**$_{[5]}$ (without guest and solvent molecules), the dashed green lines mark the heavy atom distance between the C atoms of d_{cavity} (14.004 Å, 13.453 Å, 13.797 Å), and the heavy atom distance between the C atoms of d_{gate} (7.884 Å, 9.897 Å, 9.936 Å).

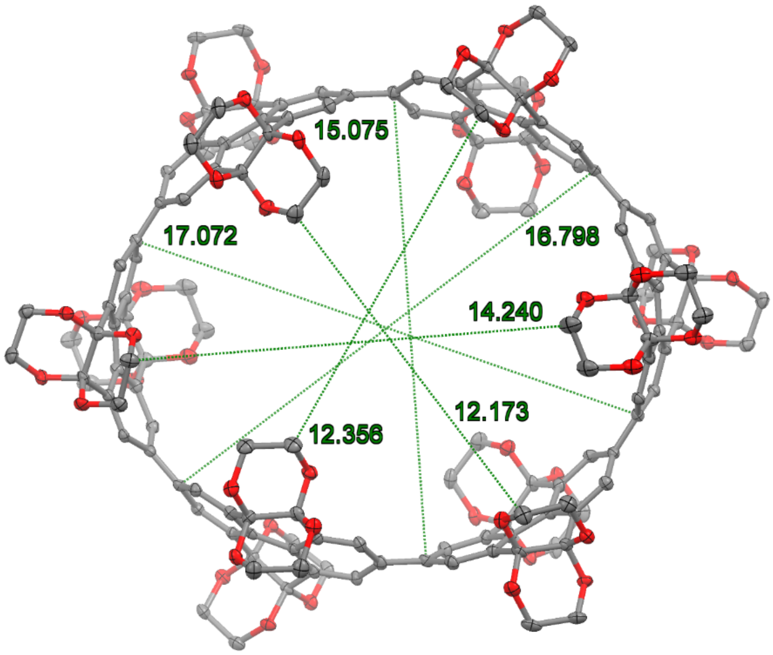

Figure S49. Single crystal X-ray structure of **1**$_{[6]}$, the dashed green lines mark the heavy atom distance between the C atoms of d_{cavity} (17.072 Å, 15.075 Å, 16.798 Å), and the heavy atom distance between the C atoms of d_{gate} (12.356 Å, 12.173 Å, 14.240 Å).

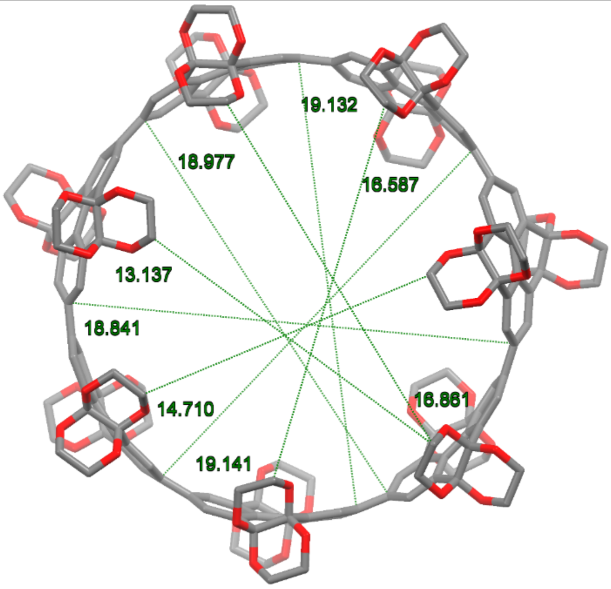

Figure S50. Single crystal X-ray structure of **1**[7], the dashed green lines mark the heavy atom distance between the C atoms of d_{cavity} (18.977 Å, 19.132 Å, 18.841 Å, 19.141 Å), and the heavy atom distance between the C atoms of d_{gate} (14.710 Å, 13.137 Å, 16.587 Å, 16.861 Å).

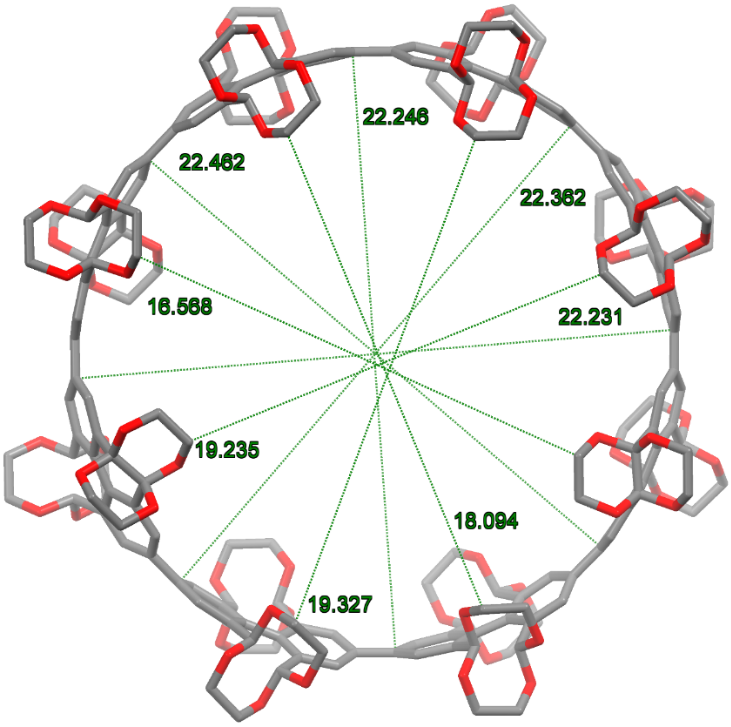

Figure S51. Single crystal X-ray structure of **1**[8], the dashed green lines mark the heavy atom distance between the C atoms of d_{cavity} (22.462 Å, 22.246 Å, 22.362 Å, 22.231 Å), and the heavy atom distance between the C atoms of d_{gate} (16.568 Å, 19.235 Å, 19.327 Å, 18.094 Å).

Table S8. The average of d_{cavity}, d_{gate}, and the corresponding ratio of the two values to determine the degree of confinement of the space within the presented macrocycles.

	$\mathbf{1}_{[4]}$	$\mathbf{1}_{[5]}$	$\mathbf{1}_{[6]}$	$\mathbf{1}_{[7]}$	$\mathbf{1}_{[8]}$
⌀ d_{cavity} [Å]	11.055	13.751	16.315	19.023	22.325
⌀ d_{gate} [Å]	7.049	9.239	12.923	15.324	18.306
ratio	0.64	0.67	0.79	0.81	0.82

The cavity volume of $\mathbf{1}_{[4–6]}$ was calculated using the MS-Roll interface of X-Seed (Version 4.04),[12] employing the default van der Waals atomic radii and a probe sphere with a radius of 1.2 Å as suggested by the developer.[13] For $\mathbf{1}_{[4]}$ a benzene molecule was placed on top and bottom of the crystal structure to prevent the probe exiting the cavity. For $\mathbf{1}_{[5]}$ one coronene and for $\mathbf{1}_{[6]}$ two benzene and one coronene molecules were placed on the top and the bottom to prevent the probe exiting the cavity. The following default van der Waals radii were used: C = 1.70 Å, H = 1.20 Å, and O = 1.52 Å. The cavity shown was imaged using the POV-Ray interface of X-Seed.

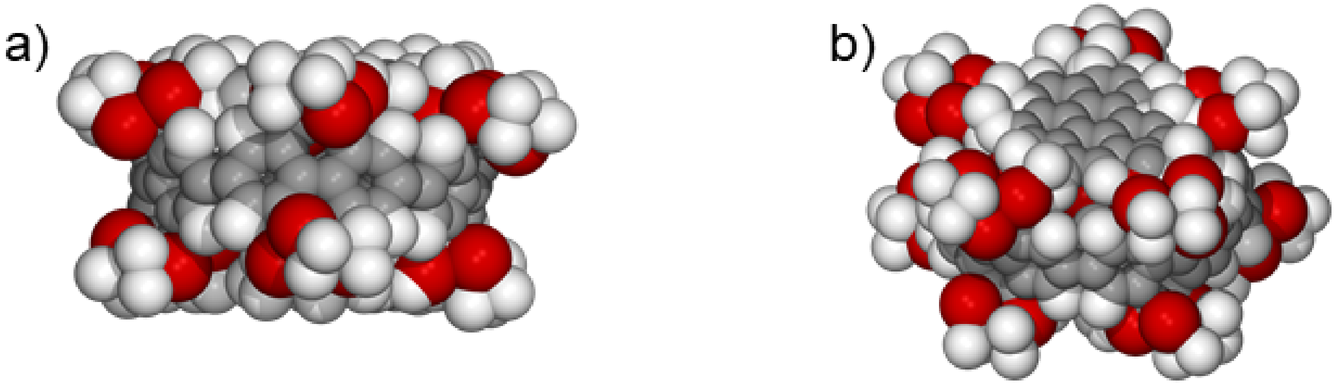

Figure S52. Van der Waals spheres of $\mathbf{1}_{[5]}$ with a modeled corannulene placed on top and bottom of $\mathbf{1}_{[5]}$ as a preparation for the MS Roll cavity volume calculation. a) side view; b) top view.

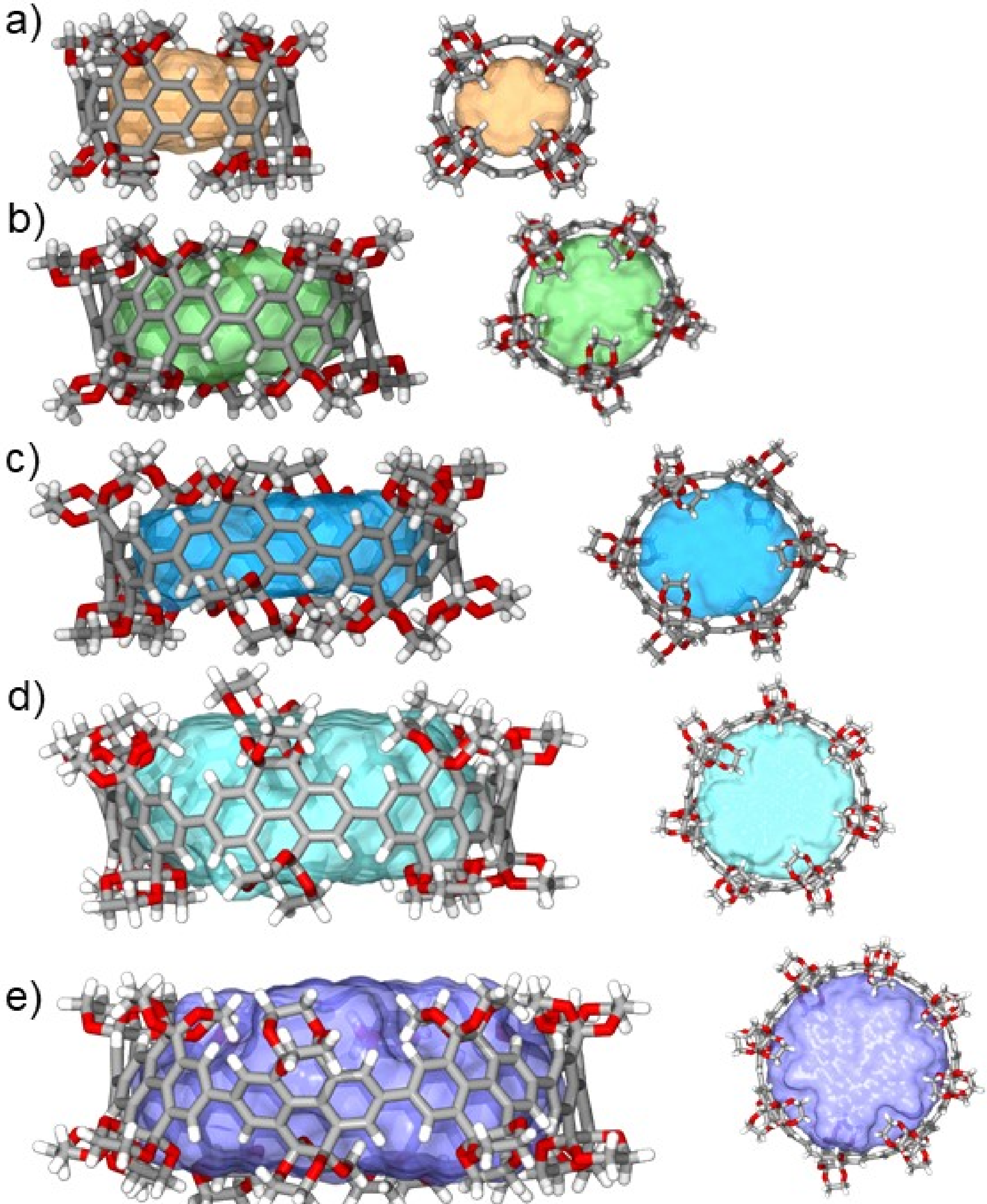

Figure S53. Single Crystal X-ray structures of **1**$_{[4–6]}$ and geometry optimized structures of **1**$_{[7–8]}$ (B3LYP-D3/def2SVP with the respective cavity volume calculated using MS Roll, a) side and top view of **1**$_{[4]}$ with a volume of 220 Å^3, b) side and top view of **1**$_{[5]}$ with a volume of 480 Å^3, c) side and top view of **1**$_{[6]}$ with a volume of 588 Å^3 d) side and top view of **1**$_{[7]}$ with a volume of 1342 Å^3 e) side and top view of **1**$_{[8]}$ with a volume of 2068 Å^3.

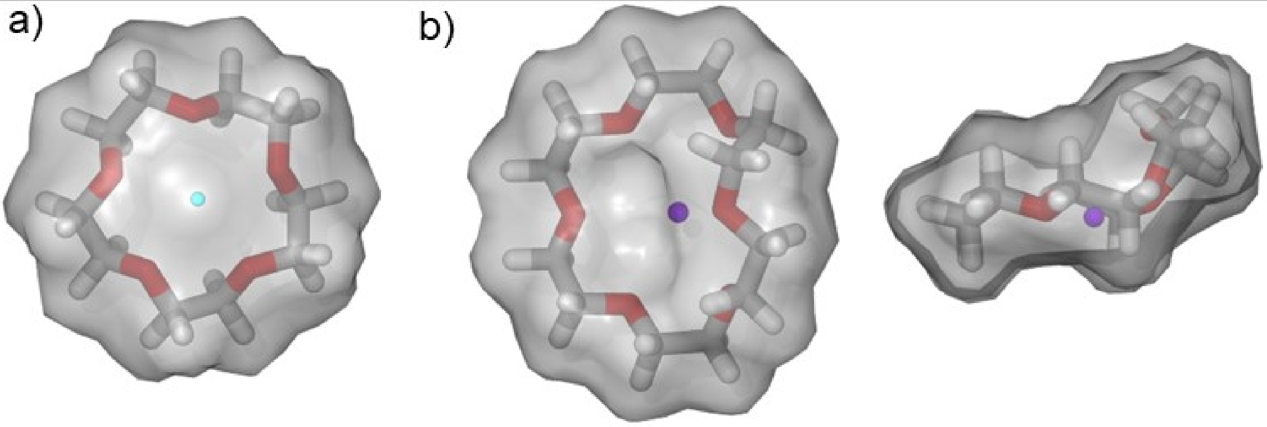

Figure S54. Graphical representation of the molecular volume calculated using the MS Roll suite of X–Seed, with a probe radius of 1.2 Å of a) 15-crown-5 · Na^{+}, geometry optimized structure at the B3LYP-D3/def2QZVP level of theory $V = 247$ Å^3, b) 18-crown-6 · K^{+} structure extracted from the crystal structure of $\mathbf{1}_{[5]}$···18-crown-6·K(BF_4) $V = 288$ Å^3.

Earlier work on packing coefficients found empirically that a packing coefficient is best in the range of (55±9)% for unpolar cavities (Mecozzi–Rebek rule).[14] The shown results for 15-crow-5 · Na^{+} and 18-crown-6 · K^{+} are in that range. The deviation might be explained by polar interactions between host and guest.

Table S9. Calculated host $\mathbf{1}_{[5]}$ and guest volumes with the respective packing coefficients.

	V(guest) [Å^3]	V(cavity of $\mathbf{1}_{[5]}$) [Å^3]	Packing Coefficient
15-crown-5· Na^{+}	247	480	51%
18-crown-6· K^{+}	288		60%

S8. UV/vis Spectroscopy

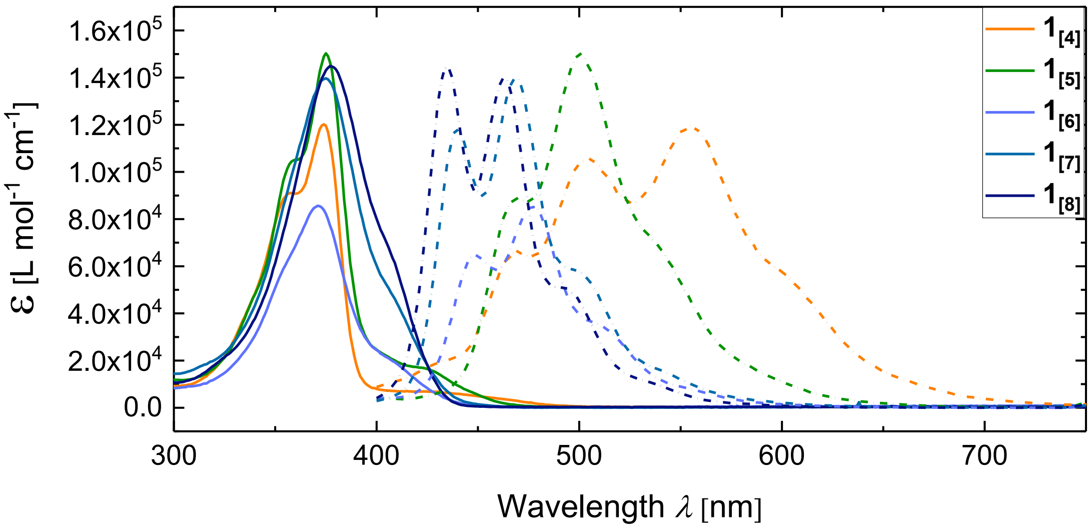

Figure S55. UV/vis absorption and emission spectra of **1**[4–8] in $CHCl_3$ at 25 °C. Concentration for absorption: $c(\mathbf{1}_{[4]}) = 6.5\times10^{-6}$ mol L^{-1}; $c(\mathbf{1}_{[5]}) = 5.8\times10^{-6}$ mol L^{-1}; $c(\mathbf{1}_{[6]}) = 5.7\times10^{-6}$ mol L^{-1}; $c(\mathbf{1}_{[7]}) = 6.8\times10^{-6}$ mol L^{-1}; $c(\mathbf{1}_{[8]}) = 4.3\times10^{-6}$ mol L^{-1}; Concentration for emission: $c(\mathbf{1}_{[4]}) = 2.5\times10^{-6}$ mol L^{-1}; $c(\mathbf{1}_{[5]}) = 2.5\times10^{-6}$ mol L^{-1}; $c(\mathbf{1}_{[6]}) = 2.4\times10^{-7}$ mol L^{-1}; $c(\mathbf{1}_{[7]}) = 1.3\times10^{-7}$ mol L^{-1}; $c(\mathbf{1}_{[8]}) = 2.8\times10^{-8}$ mol L^{-1}.

S9. Host–guest Binding Experiments by UV/vis Spectroscopy with 1[4–7]

Stock solutions containing crown ether and corresponding salts were always prepared according to the following procedure. Crown ether was dissolved in $CHCl_3$. The corresponding salts were added in a slight excess and the solution was sonicated. Afterwards the solution was filtered. This procedure allows the assumption that only insignificant amounts of free crown ether should be present. Whenever crown ether and salts were added as pure substances, ionic salts were always added in a slight excess.

1[4]: In the following graphs of this section, **1**[4] is referred to as host.

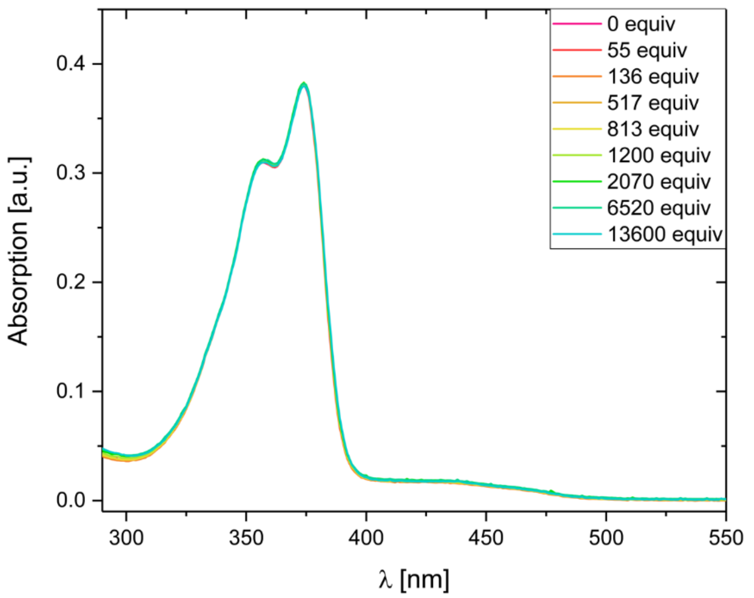

Figure S56. Isothermal UV/vis absorption binding titration. Spectra of **1**[4] at 25 °C, in $CHCl_3$, (c(**1**[4]) = 4.3×10^{-6} mol L^{-1}) upon subsequent addition of a 12-crown-4 and LiCl stock-solution in $CHCl_3$.

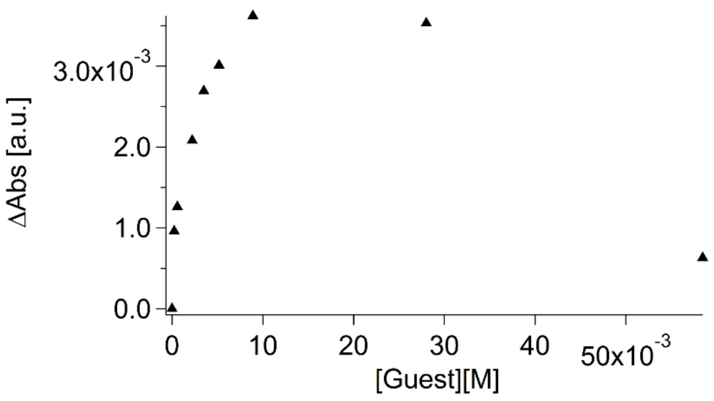

Figure S57. The difference in absorption at 375 nm plotted against the concentration of added 12-crown-4•LiCl. Non-linear least-square curve fitting is not possible with this data obtained from UV/vis spectroscopic isothermal binding titration (c(**1**[4]) = 4.3 ×10^{-6} mol L^{-1}, in $CHCl_3$ at 298 K). The insignificant change in absorption intensity originates from unspecific binding or the change in ionic strengths upon the excessive addition of LiCl salt.

1[5]: In the following, **1**[5] is referred to as host.

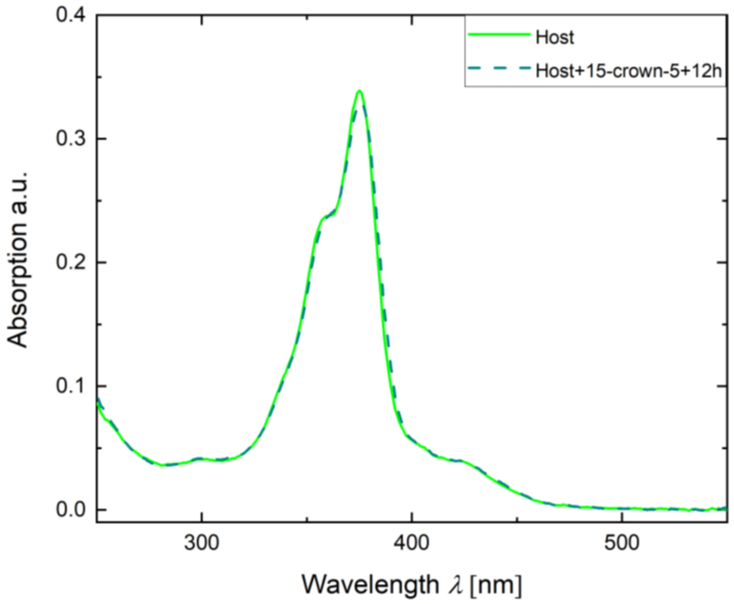

Figure S58. UV/vis absorption spectra of **1**[5] ($c = 2.1\times10^{-6}$ mol L^{-1}) in $CHCl_3$ at 25 °C before and 12 h after the addition of 1080 equiv 15-crown-5 (1.5 µL).

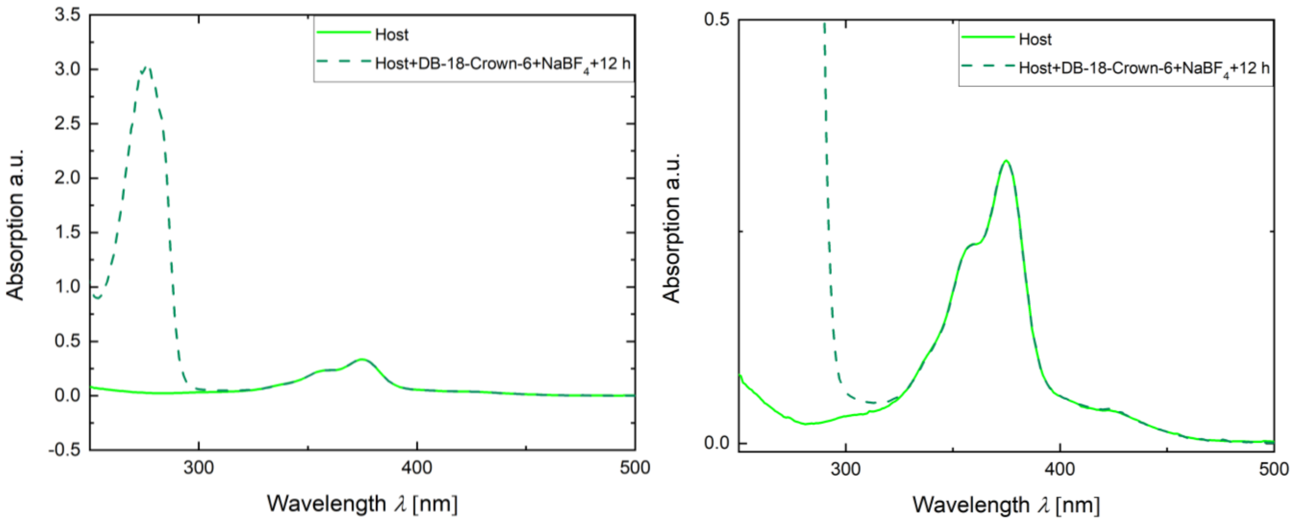

Figure S59. UV/vis absorption spectra of **1**[5] ($c = 2.1\times10^{-6}$ mol L^{-1}) in $CHCl_3$ at 25 °C before and 12 h after the addition of 310 equiv dibenzo(DB)-18-crown-6 (0.67 mg) and $NaBF_4$ (0.6 mg). The full spectra range (left) and the enlarged spectral region in regard of the host absorption (right) are shown.

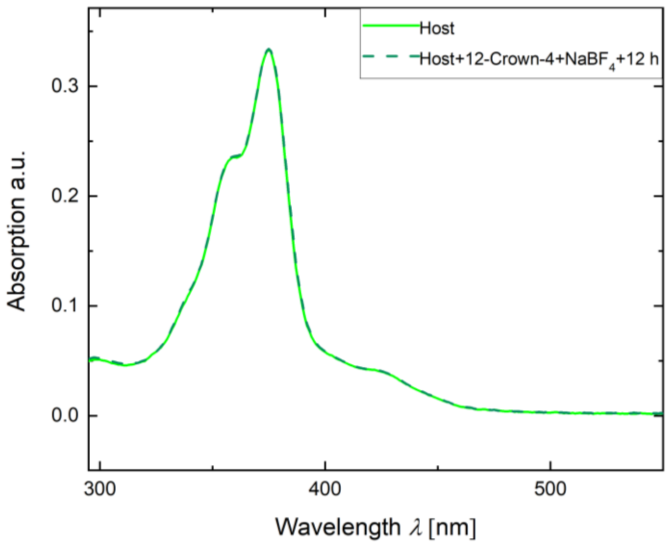

Figure S60. UV/vis absorption spectra of **1**$_{[5]}$ (c = 2.1×10^{-6} mol L^{-1}) in $CHCl_3$ at 25 °C before and 12 h after the addition of 1545 equiv 12-crown-4 (1.5 µL) and $NaBF_4$ (0.71 mg).

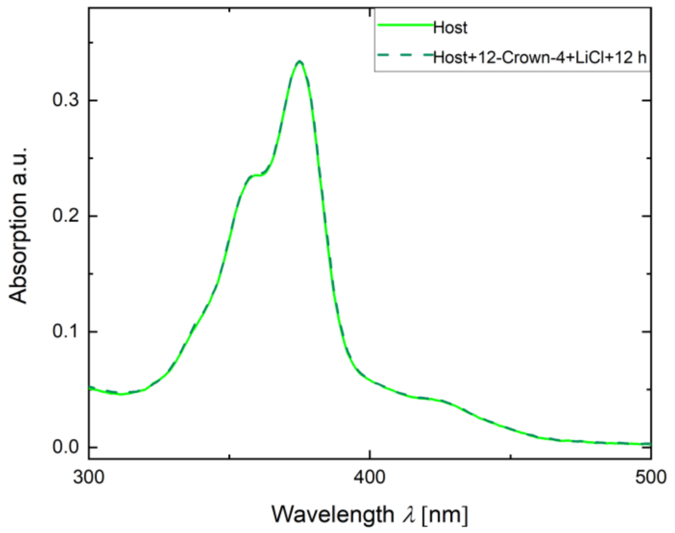

Figure S61. UV/vis absorption spectra of **1**$_{[5]}$ (c = 2.1×10^{-6} mol L^{-1}) in $CHCl_3$ at 25 °C before and 12 h after the addition of 1545 equiv 12-crown-4 (1.5 µL) and LiCl (0.5 mg).

S9.1 Isothermal UV/vis Binding Titrations

Titrations were performed to determine the association constant (K_a) of the complexation between $\mathbf{1}_{[5–6]}$, and different crown ethers with respective ions. Titrations were performed at 298 K.

Preparation of Solutions. A solution of host ($\mathbf{1}_{[5]}$) (~ 4 mM) in an appropriate solvent (typically $CHCl_3$) was prepared first. This solution was then used as parent solution to prepare a second solution as a mixture of host and guest ([G] at least 10-fold of the host concentration). The constant component during the titration is referred to as "host" and the varied component as "guest" throughout.

Association Constant Determination. A solution of a known concentrations of guest $[G]_0$ (excess) with $[H]_0$ was added successively to the parent solution of $[H]_0$ until saturation of the change in UV/vis absorption ΔA at a selected wavelength was observed. The association constants were determined by non-linear curve fitting (using Igor Pro v. 8.04 software) of the titration curves according to the Benesi–Hildebrand method. The fitting curves were obtained by plotting the absorbance changes at a selected wavelength (ΔA) for UV/vis titrations.[15] The data was fitted to the equation:[16]

$$\Delta A = \frac{\varepsilon_{host\cdot guest}}{2} \cdot \left[\left([\text{host}]_{Tot} \cdot [\text{guest}] + \frac{1}{K_a} \right) - \sqrt{\left([\text{host}]_{Tot} + [\text{guest}] + \frac{1}{K_a} \right)^2 - (4 \cdot [\text{host}]_{Tot} \cdot [\text{guest}])} \right] \qquad \text{(Equation S2)}$$

, where the unknown parameters are: $\varepsilon_{host\cdot guest}$ as the extinction coefficient of the complex and K_a as the association constant. An error of 20% was estimated for these values.

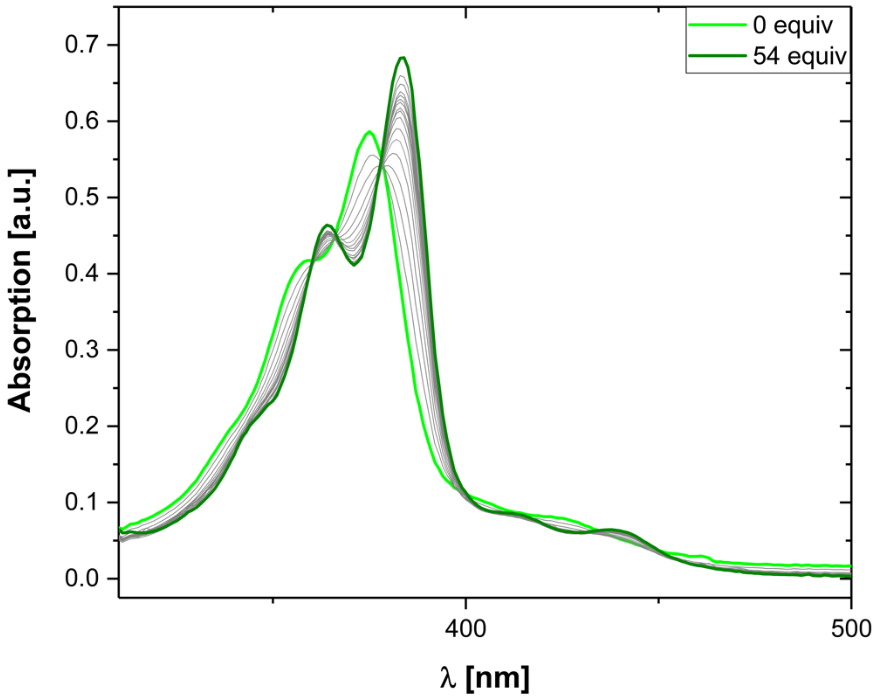

Figure S62. Isothermal UV/vis absorption binding titration. Spectra of $\mathbf{1}_{[5]}$ at 25 °C, in $CHCl_3$, ($c(\mathbf{1}_{[5]}) = 3.8\times10^{-6}$ mol L^{-1}) upon subsequent addition of a 15-crown-5 and $NaBF_4$ stock-solution in $CHCl_3$.

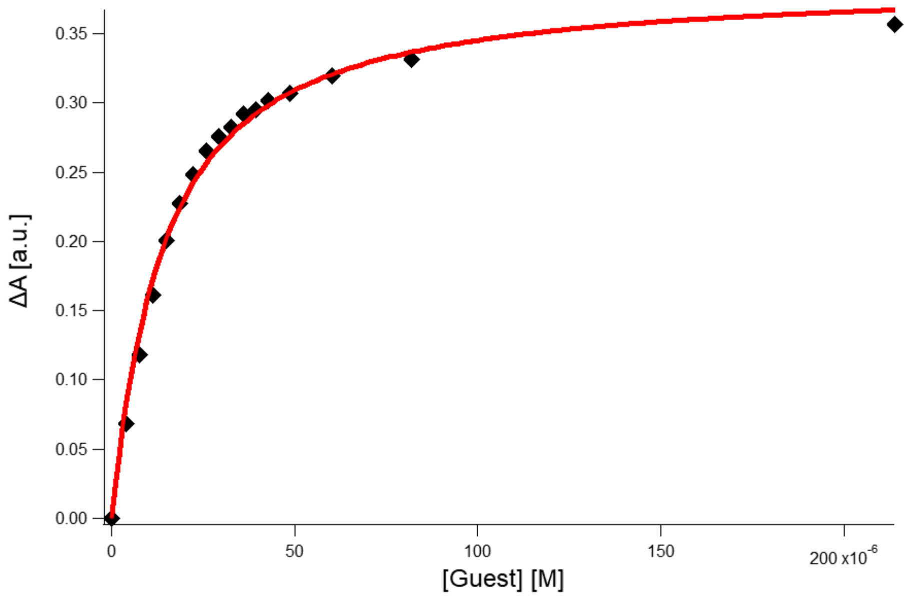

Figure S63. Non-linear least-square curve fitting analysis obtained from UV/vis spectroscopic isothermal binding titration ($c(\mathbf{1}_{[5]}) = 3.8\times10^{-6}$ mol L^{-1}, in $CHCl_3$ at 298 K) upon addition of a 15-crown-5 and $NaBF_4$ stock-solution in $CHCl_3$. ΔA taken at 385 nm. The association constant K_a was determined to be: $K_a = 8.0\times10^4$ M^{-1}.

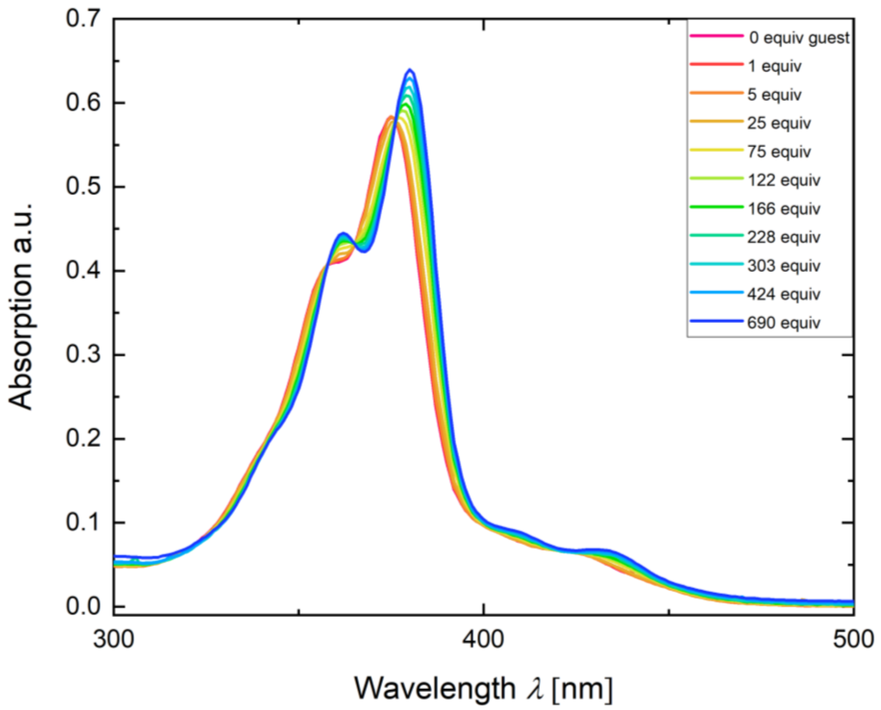

Figure S64. Isothermal UV/vis absorption binding titration. Spectra of $\mathbf{1}_{[5]}$ at 25 °C, in $CHCl_3$, ($c(\mathbf{1}_{[5]}) = 3.8\times10^{-6}$ mol L^{-1}) upon subsequent addition of a 18-crown-6 and KBF_4 stock-solution in $CHCl_3$.

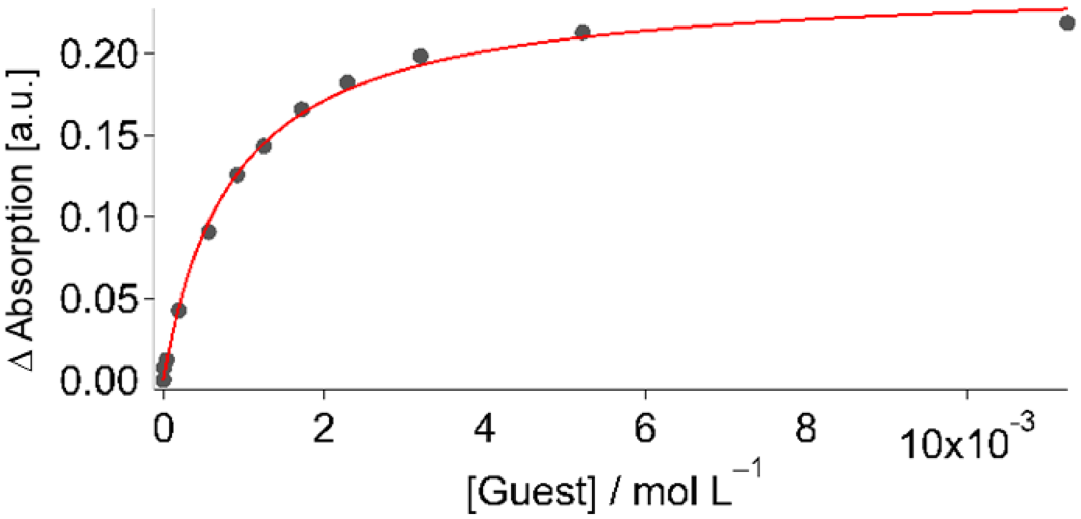

Figure S65. Non-linear least-square curve fitting analysis obtained from UV/vis spectroscopic isothermal binding titration (c(**1**$_{[5]}$) = 3.8×10^{-6} mol L^{-1}, in $CHCl_3$ at 298 K) upon addition of a 18-crown-6 and KBF_4 stock-solution in $CHCl_3$. ΔA taken at 384 nm. The association constant K_a was determined to be: $K_a = 1.2\times10^4$ M^{-1}.

1$_{[6]}$: In the following graphs of this section, **1**$_{[6]}$ is referred to as host.

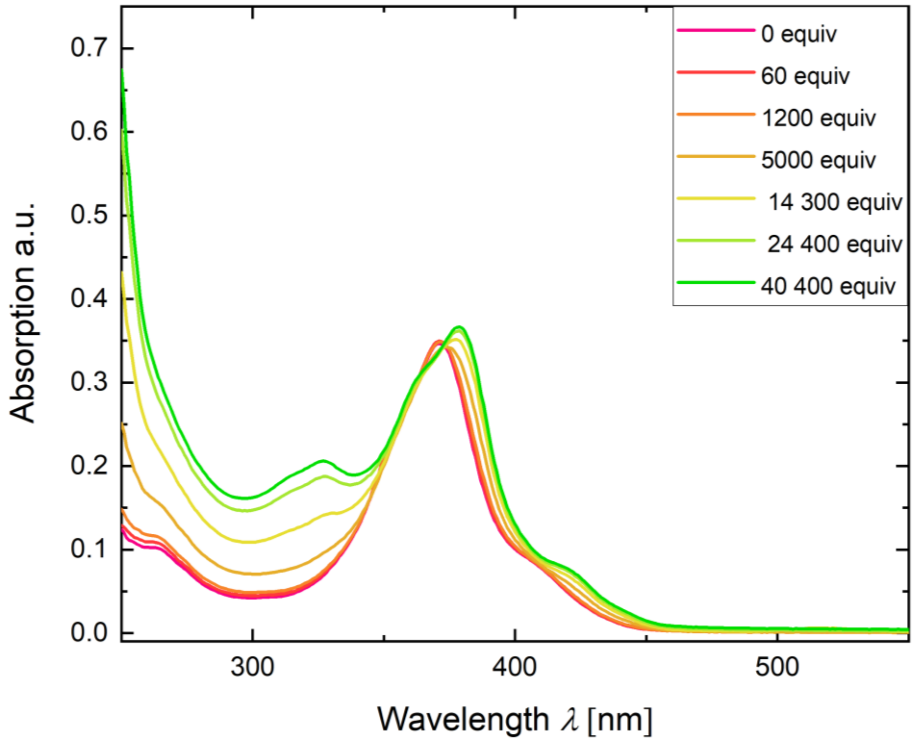

Figure S66. Isothermal UV/vis absorption binding titration. Spectra of **1**$_{[6]}$ at 25 °C, in $CHCl_3$, (c(**1**$_{[6]}$) = 3.7×10^{-6} mol L^{-1}) upon subsequent addition of a 15-crown-5 and $NaBF_4$ stock solution in $CHCl_3$.

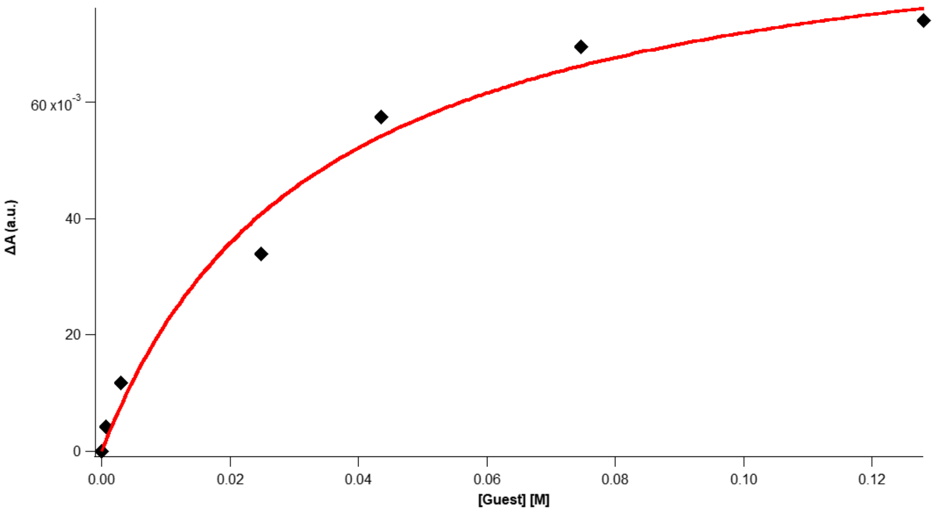

Figure S67. Non-linear least-square curve fitting analysis obtained from UV/vis spectroscopic isothermal binding titration (c(**1**$_{[6]}$) = 3.7×10^{-6} mol L^{-1}, in $CHCl_3$ at 298 K) upon addition of a 15-crown-5 and $NaBF_4$ stock solution in $CHCl_3$. ΔA taken at 380 nm. The association constant K_a was determined to be: $K_a = 28$ M^{-1}.

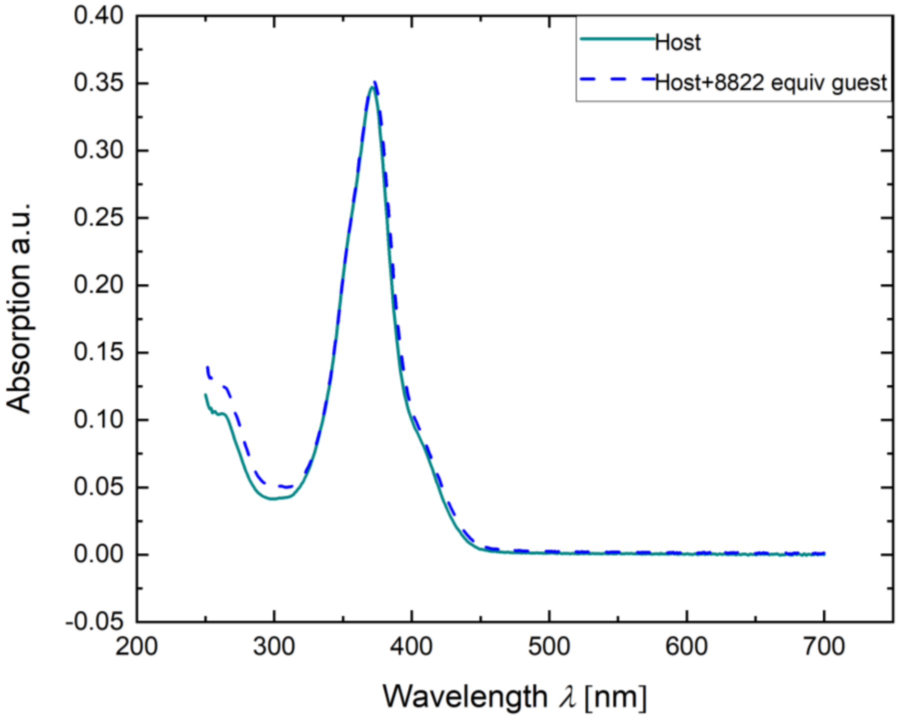

Figure S68. UV/vis absorption spectra of **1**[6] at 25 °C, in $CHCl_3$, (c(**1**[6]) = 3.7×10^{-6} M) upon addition of 8822 equiv 18-crown-6 and KBF_4 via stock solution in $CHCl_3$.

1[7]: In the following graphs of this section, **1**[7] is referred to as host.

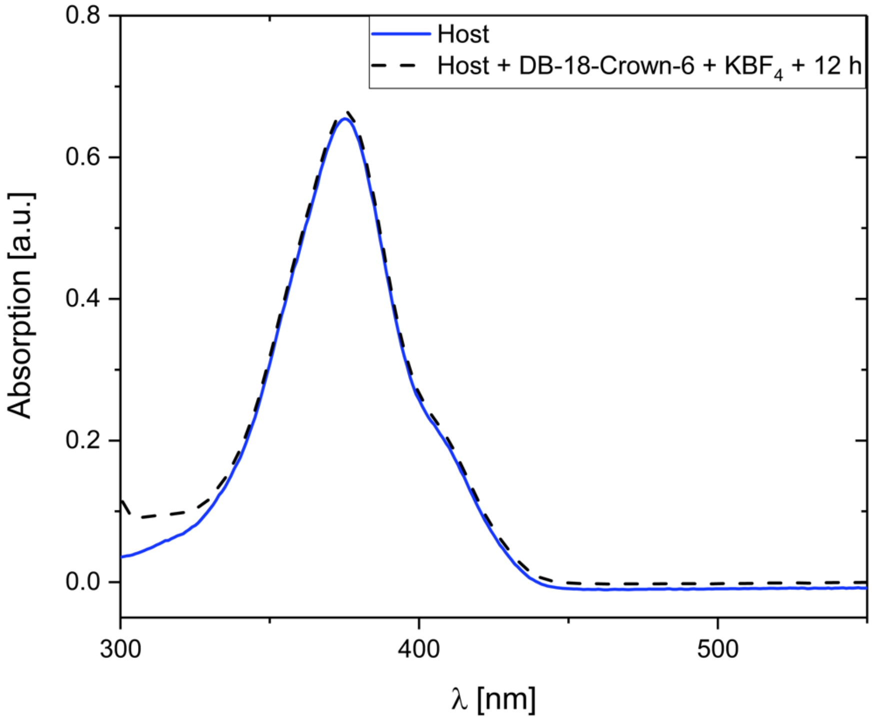

Figure S69. UV/vis absorption spectra of **1**[7] at 25 °C, in $CHCl_3$, (c(**1**[7]) = 3.6×10^{-6} M) upon addition of 870 equiv (3.0 mg) dibenzo-18-crown-6 and KBF_4 (2.4 mg 1990 equiv).

[10]CPP: In the following graphs of this section, **[10]CPP** is referred to as host.

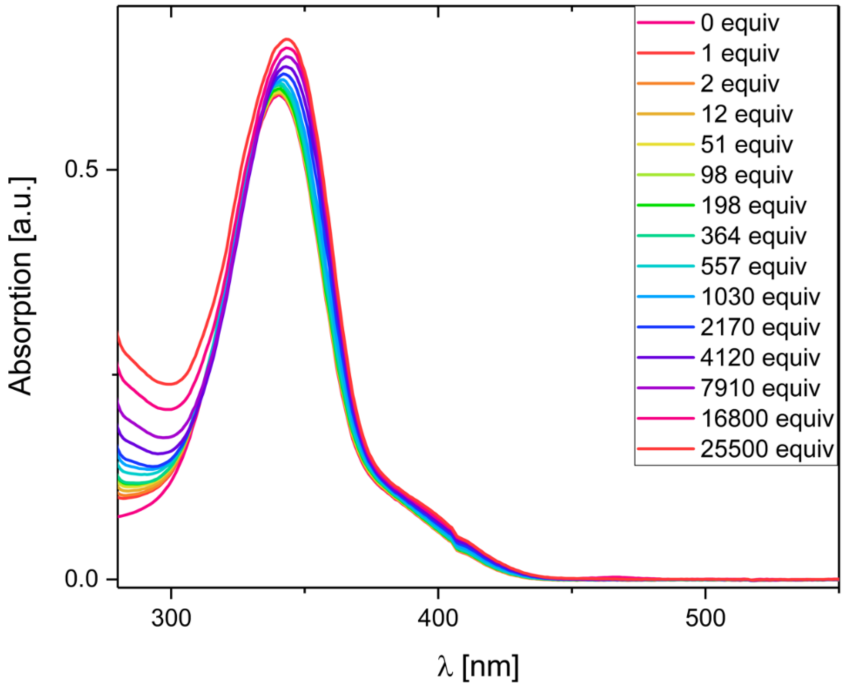

Figure S70. Isothermal UV/vis absorption binding titration. Spectra of **[10]CPP** at 25 °C, in $CHCl_3$, (c(**[10]CPP**) = 3.8×10^{-6} mol L^{-1}) upon subsequent addition of a 15-crown-5 and $NaBF_4$ stock-solution in $CHCl_3$.

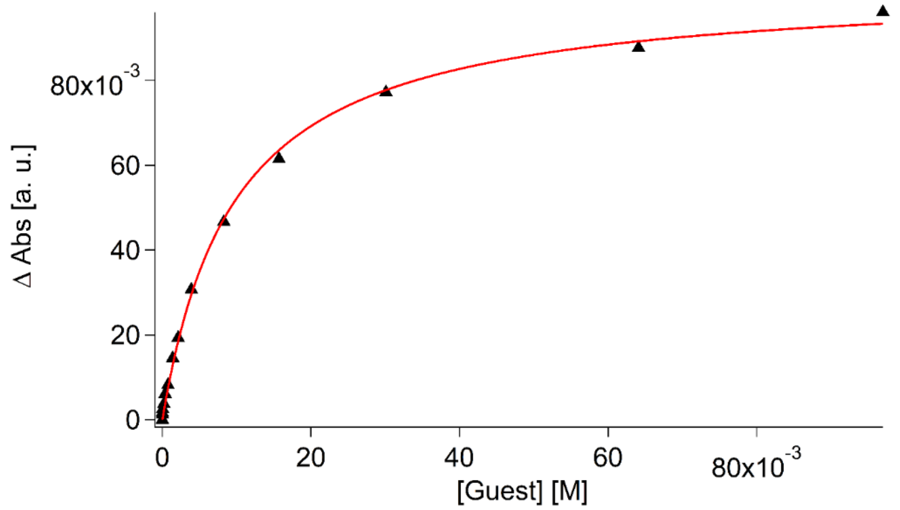

Figure S71. Non-linear least-square curve fitting analysis obtained from UV/vis spectroscopic isothermal binding titration (c(**[10]CPP**) = 3.8×10^{-6} mol L^{-1}, in $CHCl_3$ at 298 K) upon addition of a 15-crown-5 and $NaBF_4$ stock solution in $CHCl_3$. ΔA taken at 347 nm. The association constant K_a was determined to be: $K_a = 103$ M^{-1}.

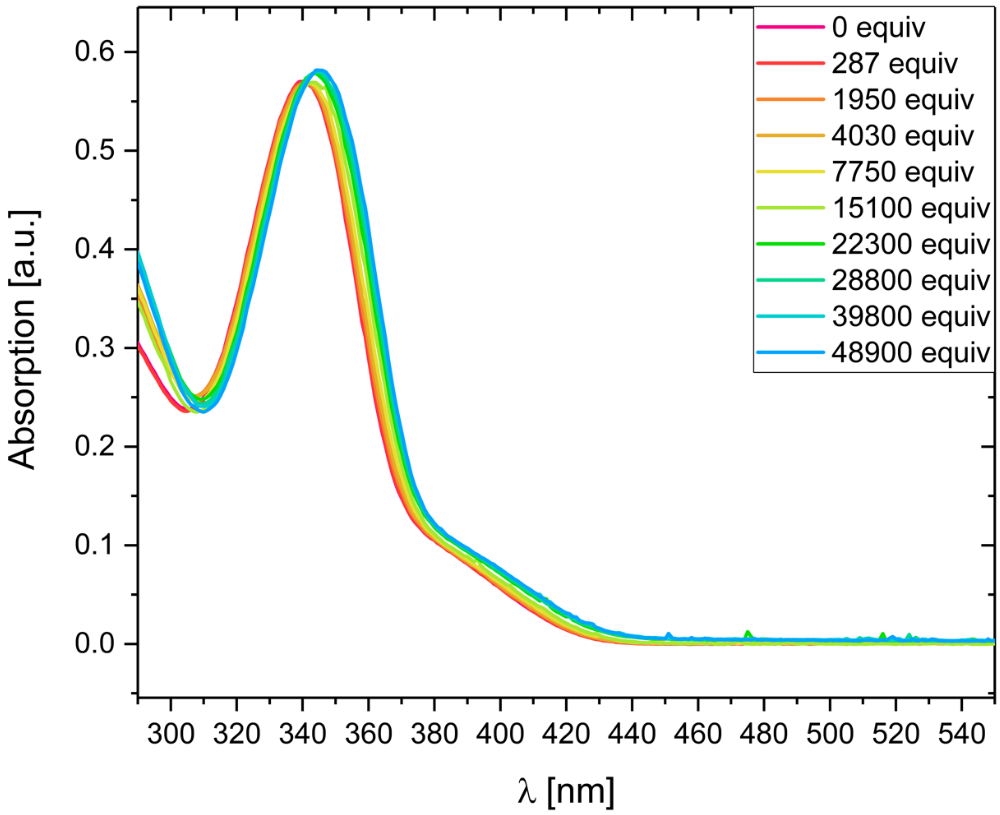

Figure S72. Isothermal UV/vis absorption binding titration. Spectra of **[10]CPP** at 25 °C, in $CHCl_3$, (c(**[10]CPP**) = 3.8×10^{-6} mol L^{-1}) upon subsequent addition of 18-crown-6 and KBF_4 stock-solution in $CHCl_3$.

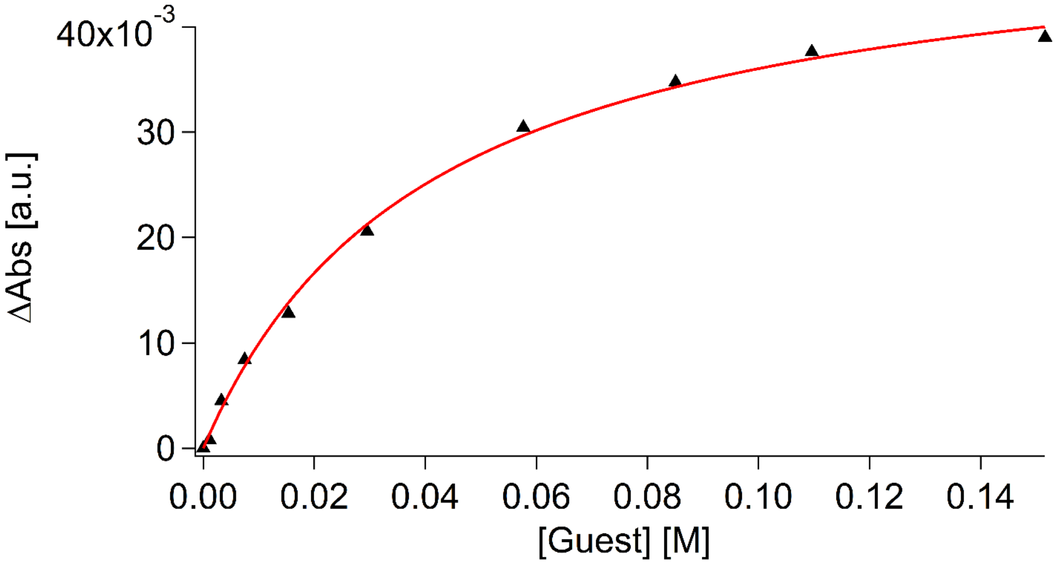

Figure S73. Non-linear least-square curve fitting analysis obtained from UV/vis spectroscopic isothermal binding titration (c(**[10]CPP**) = 3.8×10^{-6} mol L^{-1}, in $CHCl_3$ at 298 K) upon addition of a 18-crown-6 and KBF_4 stock solution in $CHCl_3$. ΔA taken at 346 nm. The association constant K_a was determined to be: $K_a = 24$ M^{-1}.

S9.2 ^{1}H NMR Binding Titration at Slow Exchange of $\mathbf{1}_{[5]}$···(15-crown-5·NaBF$_4$)

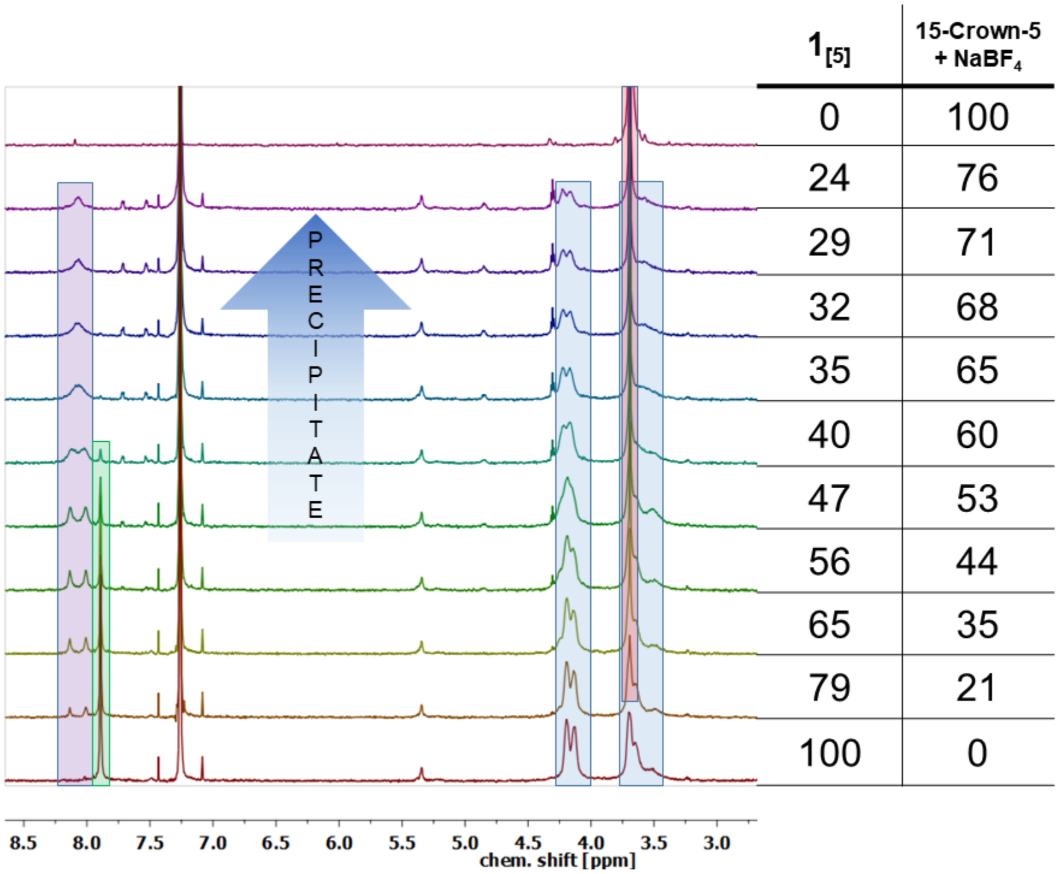

$\mathbf{1}_{[5]}$	15-Crown-5 + NaBF$_4$
0	100
24	76
29	71
32	68
35	65
40	60
47	53
56	44
65	35
79	21
100	0

Figure S74. Isothermal ^{1}H NMR binding titration (600 MHz, 25 °C, in CDCl$_3$) $c(\mathbf{1}_{[5]}) = 1.3\times10^{-3}$ M; The table on the right shows the molar distribution between [$\mathbf{1}_{[5]}$] and 15-crown-5/NaBF$_4$. At a molar ratio of 35:65 precipitation within the NMR tube was observed by eye (as indicated by the blue arrow).

Two distinct peaks evolve upon addition of 15-crown-5/NaBF$_4$ (Figure S74), assuming that these result from slow exchange, allows to determine the binding constant using the following equation

$$K_a = \frac{[HG]}{([H]_0 - a \cdot [HG])^a - ([G]_0 - b \cdot [HG])^b} \qquad \text{(Equation S3)}$$

, where $[H]_0$ is the host concentration, $[HG]$ is the concentration of the host-guest complex, $[G]$ is the concentration of the guest, a is the stoichiometry of the host and b is the stoichiometry of the guest. The concentration of the host-guest complex can be determined with the following equation

$$[HG] = \frac{n}{n+m}\frac{[H]_0}{a} \qquad \text{(Equation S4)}$$

, where n is the integral of the host-guest and m is the integral of the free host complex.

Three values are obtained for different ratios of [host]/[guest] and displayed in Table S10. The obtained values are prone to error due to precipitation, and are roughly in agreement with the binding obtained from UV-vis binding titrations.

Table S10. Calculated binding constants K_a at different host guest ratios using the integrals *n*, *m* of Figure S74. Guest: 15-crown-5/$NaBF_4$.

ratio (**1**[5]/guest)	65:35	56:44	47:53	**Ø**
$\frac{n}{m}$	0.35	1.00	0.74	–
K_a	1.3×10^3 M^{-1}	1.5×10^3 M^{-1}	31.3×10^3 M^{-1}	**1.1×10^4 M^{-1}**

S9.3 ^{1}H NMR of $\mathbf{1}_{[5]}$ and C_{60}

All spectra were recorded on samples, which were preheated to reflux and allowed to cool down to 25°C again.

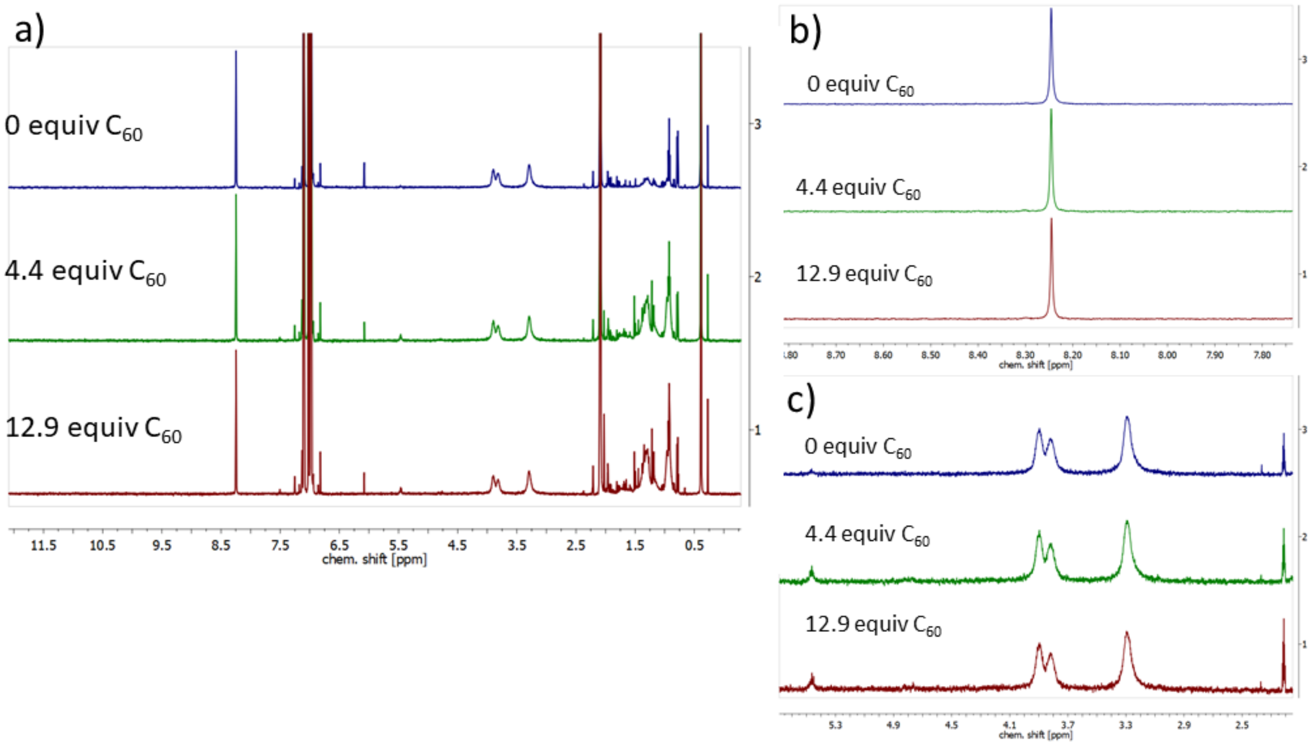

Figure S75. ^{1}H NMR spectra of $\mathbf{1}_{[5]}$ (500 MHz, 25 °C) in toluene-d_8 a) full spectrum, b) aromatic region, and c) showing the region of the ethylene glycol groups; upon addition of 0 equiv C_{60} $c(\mathbf{1}_{[5]}) = 4.77\times10^{-4}$ M (blue spectrum); 4.4 equiv C_{60} $c(\mathbf{1}_{[5]}) = 2.78\times10^{-4}$ M (green spectrum); 12.9 equiv C_{60} $c(\mathbf{1}_{[5]}) = 2.78\times10^{-4}$ M (red spectrum). No binding is detected.

The nuclear framework diameter of C_{60} is 7.1 Å, taking into account the van der Waals radii (2x1.7 Å) this adds up to 10.5 Å. The measured average diameter of the gate of $\mathbf{1}_{[5]}$ extracted from the crystal structure is 5.8 Å at the van der Waals radii. We therefore assume, that C_{60} cannot pass the gate of $\mathbf{1}_{[5]}$.

S9.4 ^{1}H NMR of $\mathbf{1}_{[6]}$ and C60

All spectra were recorded on samples, which were preheated to reflux and allowed to cool down to 25°C again. The low solubility of $\mathbf{1}_{[6]}$ excludes NMR spectroscopy with all common deuterated solvents, except for $CDCl_3$.

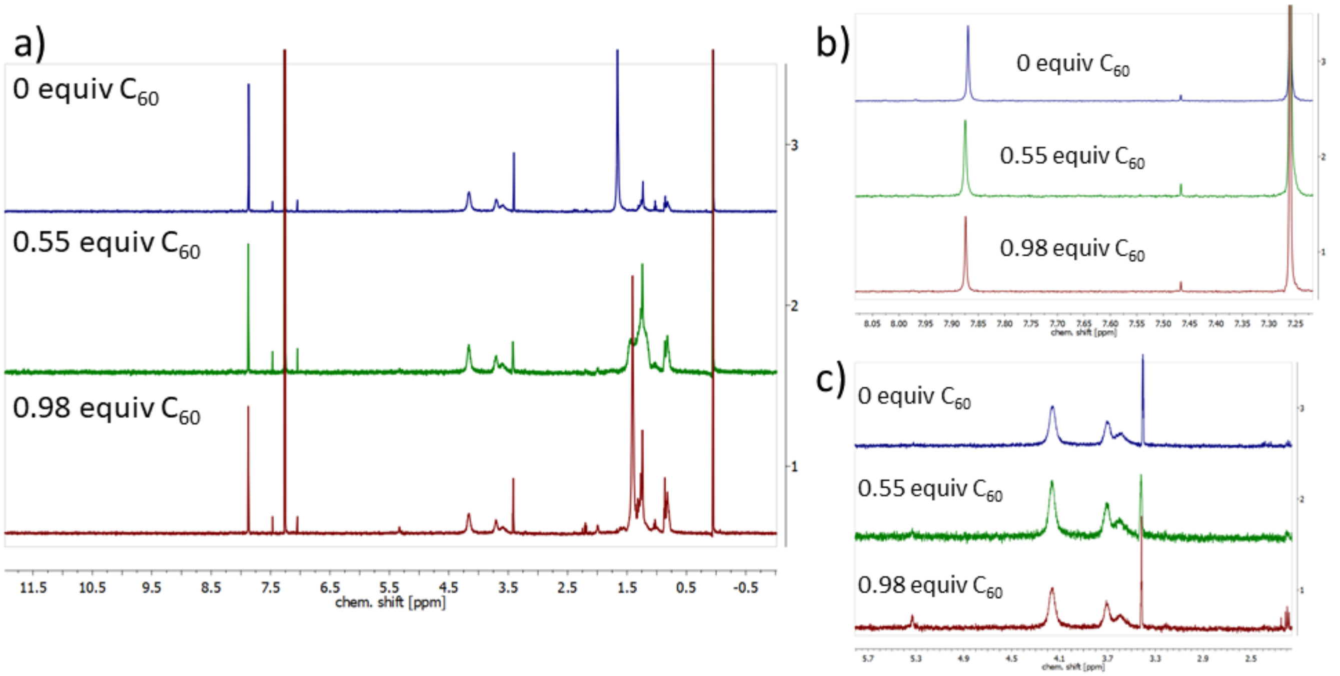

Figure S76. ^{1}H NMR spectra of $\mathbf{1}_{[6]}$ (500 MHz, 25 °C, in $CDCl_3$) a) full spectrum, b) aromatic region, and c) showing the region of the ethylene glycol groups; upon addition of 0 equiv C_{60}, $c(\mathbf{1}_{[6]}) = 2.11\times10^{-4}$ M (blue spectra); 0.55 equiv C_{60}, $c(\mathbf{1}_{[6]}) = 1.58\times10^{-4}$ M (green spectra); 0.98 equiv C_{60}, $c(\mathbf{1}_{[6]}) = 1.76\times10^{-4}$ M (red spectra). No binding is detected.

S9.5 ¹H NMR binding spectroscopy of [10]CPP and 15-crown-5·$NaBF_4$

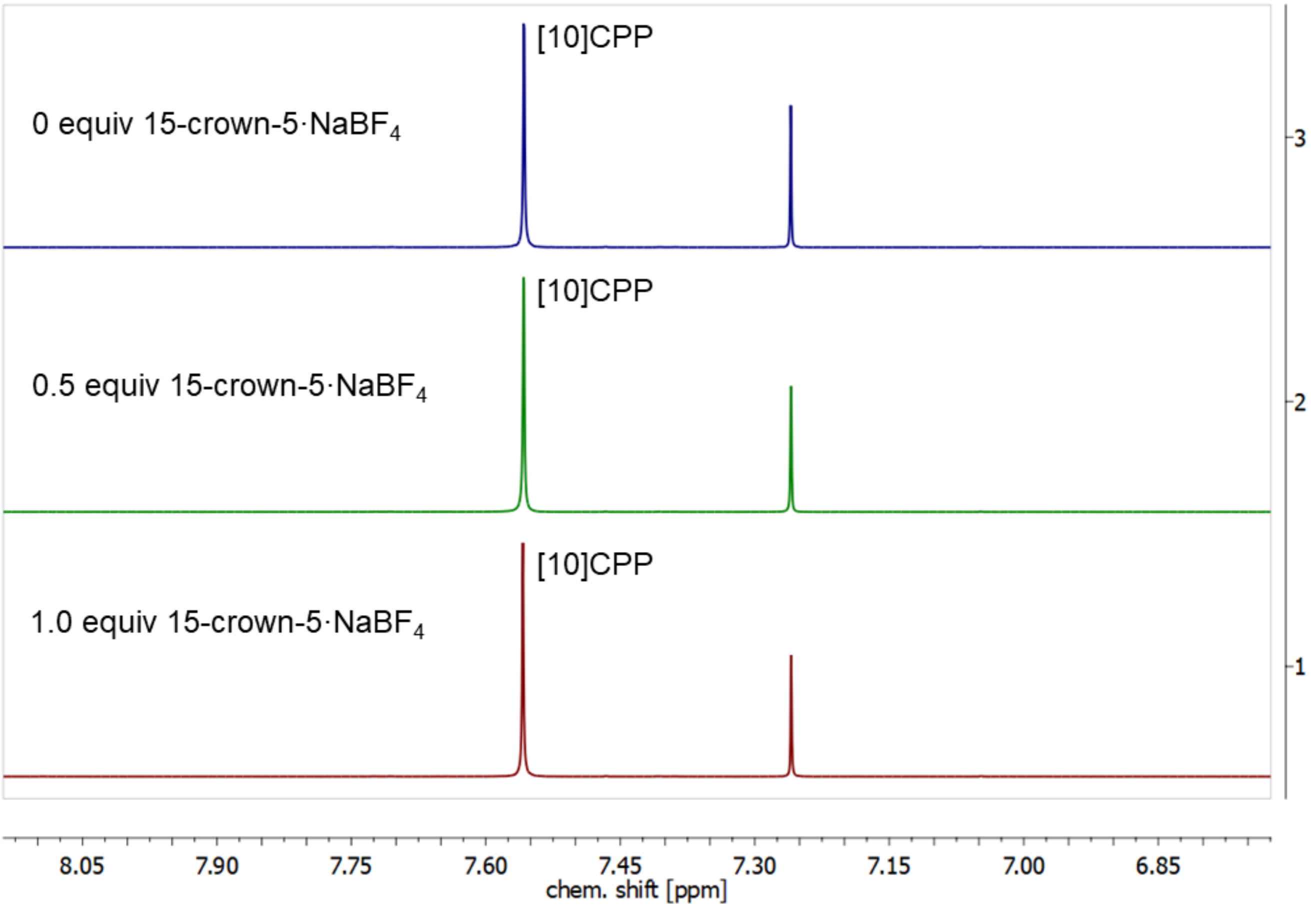

Figure S77. ¹H NMR spectra of **[10]CPP** (c(**[10]CPP**) = 1.49×10^{-4} M, 500 MHz, 25 °C, in $CDCl_3$) showing the aromatic region upon addition of 0 equiv 15-crown-5•$NaBF_4$ (blue spectra); 0.5 equiv 15-crown-5•$NaBF_4$ (green spectra); 1.0 equiv 15-crown-5•$NaBF_4$ (red spectra). No specific binding is detected by these NMR experiments. See also the corresponding UV/vis binding titrations, which revealed an association constant of K_a = 103 M^{-1} (298 K, $CHCl_3$).

S9.6 ^{1}H NMR binding spectroscopy of [10]CPP and 18-crown-6·KBF_4

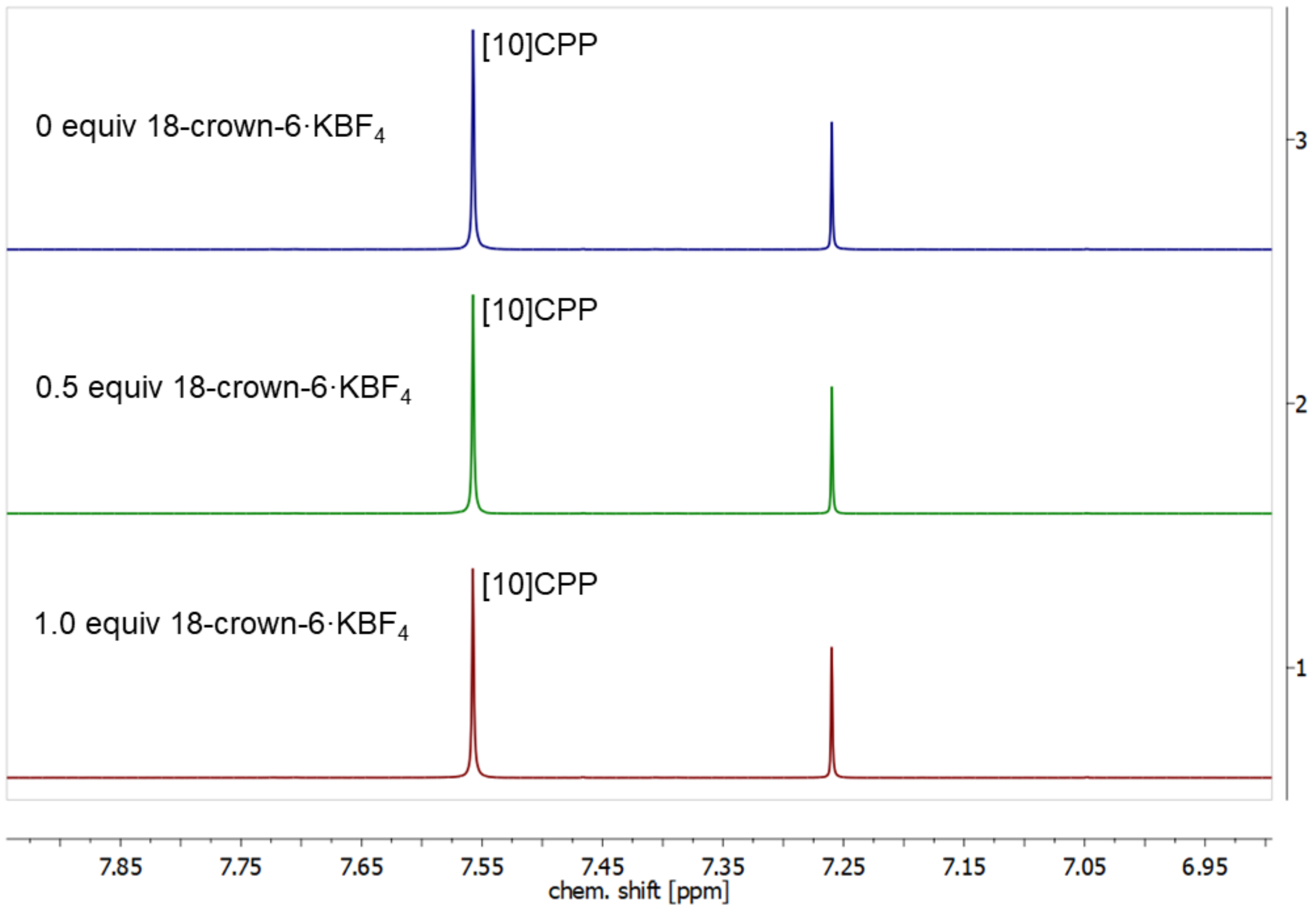

Figure S78. ^{1}H NMR spectra of **[10]CPP** (c(**[10]CPP**) = 1.49×10^{-3} M, 500 MHz, 25 °C, in $CDCl_3$) showing the aromatic region upon addition of 0 equiv 18-crown-6•KBF_4 (blue spectra); 0.5 equiv 18-crown-6•KBF_4 (green spectra); 1.0 equiv 18-crown-6•KBF_4 (red spectra). No binding is detected by these NMR experiments. See also the corresponding UV/vis binding titrations, which revealed an association constant of K_a = 24 M^{-1} (298 K, $CHCl_3$).

S10. Computational Methods

All calculations were conducted with Gaussian 16 Revision A.03 on the chccs-cluster of the Department of Chemistry of the Humboldt University of Berlin. The structures are confirmed ground-state minima according to the analysis of their analytical frequencies computed at the same level, which show no imaginary frequencies. The polarizable continuum model (PCM)[17] was used as solvent model and dichloromethane as solvent. The ring strains of the macrocycles were calculated using the homodesmotic equation (Figure S89). Homodesmotic reactions are a special case of the isodesmic reaction.[18] While in isodesmic reactions the type of bond broken in the reactant must be the same type of bonds that formed in the product,[19] in homodesmotic reactions the orbital hybridizations must also stay the same. The ring strain is the energetic difference between reactants and products.

S10.1 Geometry-Optimized Structures and Vertical Transitions

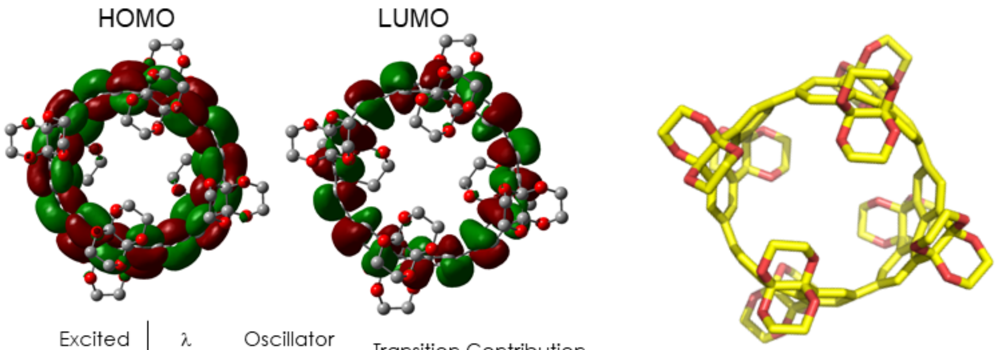

Excited State	λ [nm]	Oscillator Strength	Transition Contribution	
1	404.8	0.0000	454 -> 459	22%
			455 -> 458	22%
			**456 -> 457**	**62%**
2	336.5	1.5012	**455 -> 457**	**47%**
			**456 -> 458**	**45%**
			456 -> 459	16%
3	336.5	1.5013	**454 -> 457**	**47%**
			456 -> 458	16%
			**456 -> 459**	**45%**
4	296.1	0.0006	452 -> 457	13%
			454 -> 462	26%
			455 -> 461	26%
			456 -> 460	50%

453 = HOMO -3
454 = HOMO -2
455 = HOMO -1
456 = HOMO

457= LUMO
458 = LUMO +1
459 = LUMO +2
460 = LUMO +3

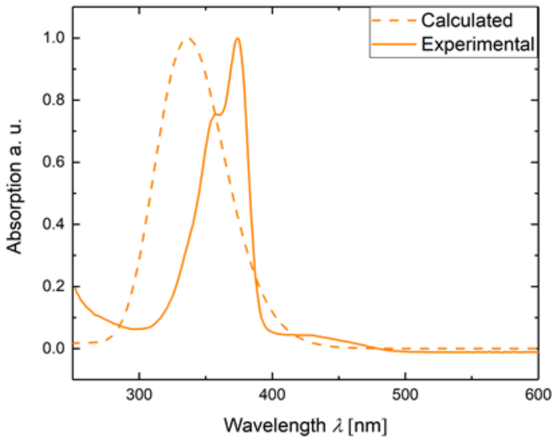

Figure S79. HOMO, LUMO representation, as well as geometry optimized structure of **1**$_{[4]}$ (level of theory: B3LYP-D3/def2SVP) (top). Calculated transitions (level of theory: TD-DFT/cam-B3LYP/6-31G(d,p)) and respective calculated UV/vis absorption spectrum compared to the experimental one.

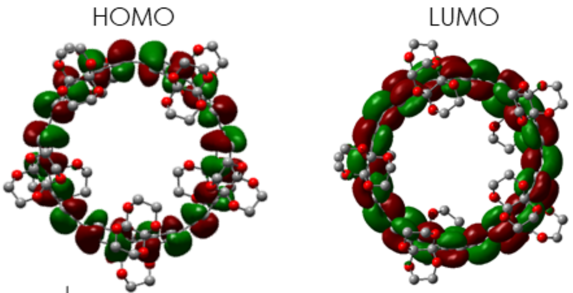

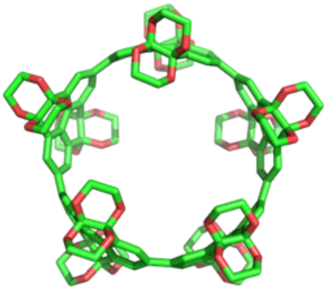

Excited State	λ [nm]	Oscillator Strength	Transition Contribution
1	396.2	0.0180	568 -> 573 26%<br>569 -> 572 28%<br>**570 -> 571 57%**
2	351.2	2.1252	566 -> 572 11%<br>567 -> 573 11%<br>568 -> 577 10%<br>**569 -> 571 46%**<br>**570 -> 572 46%**
3	345.8	2.1876	566 -> 573 12%<br>567 -> 572 12%<br>**568 -> 571 46%**<br>569 -> 577 11%<br>**570 -> 573 46%**
5	291.9	0.0296	566 -> 571 31%<br>568 -> 573 35%<br>569 -> 572 35%<br>570 -> 576 28%<br>570 -> 578 16%

567 = HOMO -3
568 = HOMO -2
569 = HOMO -1
570 = HOMO
571 = LUMO
572 = LUMO +1
573 = LUMO +2
574 = LUMO +3

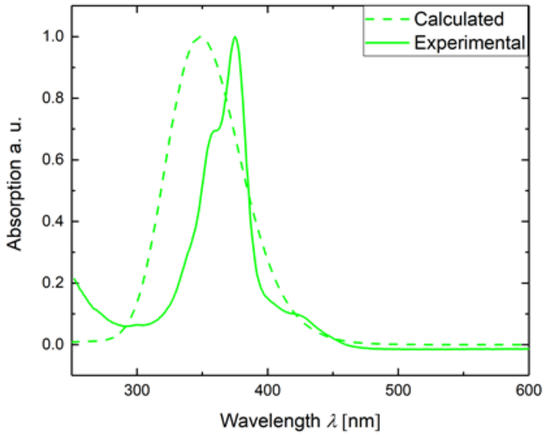

Figure S80. HOMO, LUMO representation, as well as geometry optimized structure of **1**[5] (level of theory: B3LYP-D3/def2SVP) (top). Calculated transitions (level of theory: TD-DFT/cam-B3LYP/6-31G(d,p)) and respective calculated UV/vis absorption spectrum compared to the experimental one.

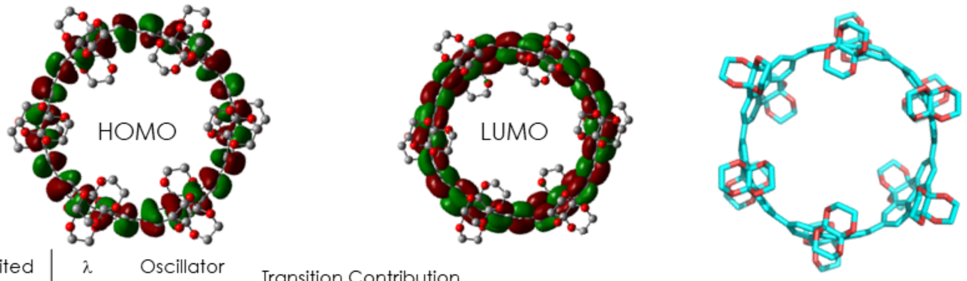

Excited State	λ [nm]	Oscillator Strength	Transition Contribution	
1	381.3	0.0000	680 -> 689	12%
			681 -> 688	13%
			682 -> 687	29%
			683 -> 686	32%
			**684 -> 685**	**52%**
2	351.8	2.7535	680 -> 686	13%
			681 -> 687	14%
			682 -> 688	13%
			**683 -> 685**	**45%**
			683 -> 689	13%
			**684 -> 686**	**44%**
3	346.4	2.8224	680 -> 687	14%
			681 -> 686	15%
			**682 -> 685**	**44%**
			682 -> 689	14%
			683 -> 688	14%
			**684 -> 687**	**44%**
5	302.0	0.0251	680 -> 685	32%
			682 -> 687	33%
			683 -> 686	31%
			683 -> 687	10%
			683 -> 696	13%
			684 -> 689	32%

681 = HOMO -3
682 = HOMO -2
683 = HOMO -1
684 = HOMO

685 = LUMO
686 = LUMO +1
687 = LUMO +2
688 = LUMO +3

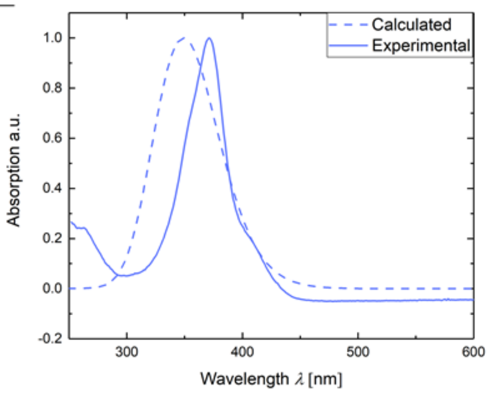

Figure S81. HOMO, LUMO representation, as well as geometry optimized structure of $\mathbf{1}_{[6]}$ (level of theory: B3LYP-D3/def2SVP) (top). Calculated transitions (level of theory: TD-DFT/cam-B3LYP/6-31G(d,p)) and respective calculated UV/vis absorption spectrum compared to the experimental one.

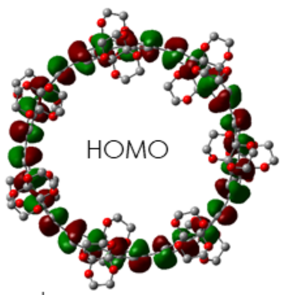

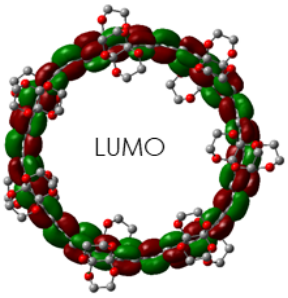

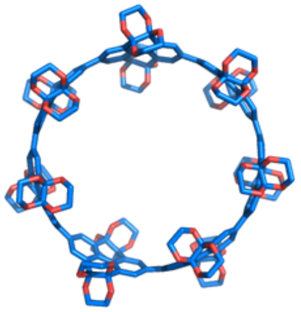

Excited State	λ [nm]	Oscillator Strength	Transition Contribution		
1	378.5	0.0390	794 -> 803	15%	794 = HOMO -4
			795 -> 802	15%	795 = HOMO -3
			796 -> 801	30%	796 = HOMO -2
			797 -> 800	33%	797 = HOMO -1
			**798 -> 799**	**48%**	798 = HOMO
2	355.4	3.4008	794 -> 800	12%	
			795 -> 800	10%	799 = LUMO
			795 -> 801	12%	800 = LUMO +1
			796 -> 802	12%	801 =LUMO +2
			**797 -> 799**	**43%**	802 = LUMO +3
			797 -> 802	10%	803 =LUMO +4
			797 -> 803	12%	
			**798 -> 800**	**43%**	
3	351.2	3.4347	794 -> 800	11%	
			794 -> 801	12%	
			795 -> 800	12%	
			795 -> 801	11%	
			**796 -> 799**	**42%**	
			796 -> 802	10%	
			796 -> 803	12%	
			797 -> 802	13%	
			**798 -> 801**	**42%**	

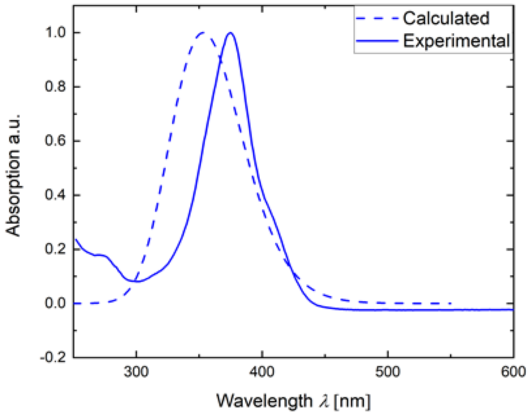

Figure S82. HOMO, LUMO representation, as well as geometry optimized structure of **1**[7] (level of theory: B3LYP-D3/def2SVP) (top). Calculated transitions (level of theory: TD-DFT/cam-B3LYP/6-31G(d,p)) and respective calculated UV/vis absorption spectrum compared to the experimental one.

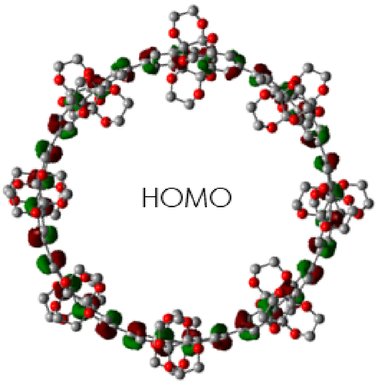

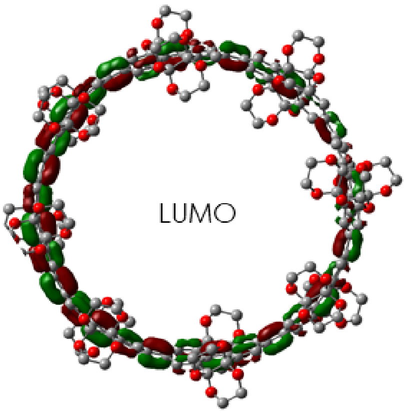

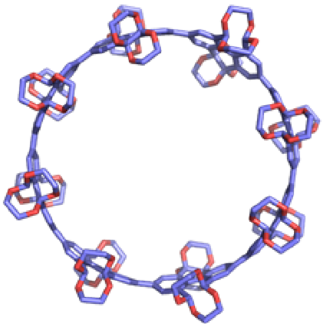

Excited State	λ [nm]	Oscillator Strength	Transition Contribution		
1	377.1	0.2237	908 -> 917	16%	908 = HOMO -4
			909 -> 916	17%	909 = HOMO -3
			910 -> 915	29%	910 = HOMO -2
			911 -> 914	32%	911 = HOMO -1
			**912 -> 913**	**46%**	912 = HOMO
2	357.9	3.8830	908 -> 915	15%	913 = LUMO
			909 -> 914	16%	914 = LUMO +1
			910 -> 917	15%	915 =LUMO +2
			**911 -> 913**	**39%**	916 = LUMO +3
			911 -> 914	15%	917 =LUMO +4
			911 -> 916	16%	
			**912 -> 914**	**39%**	
3	353.5	4.0549	908 -> 914	16%	
			909 -> 915	18%	
			**910 -> 913**	**39%**	
			910 -> 915	11%	
			910 -> 916	18%	
			911 -> 917	16%	
			**912 -> 915**	**39%**	

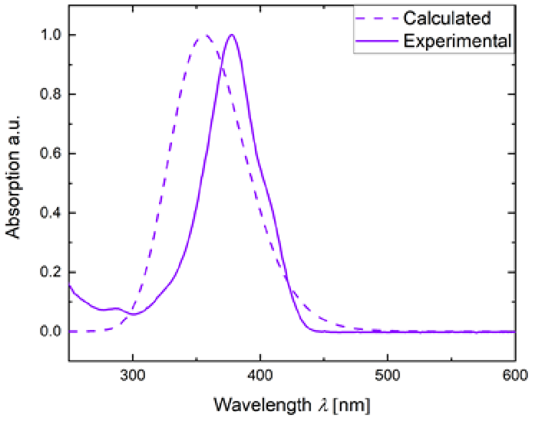

Figure S83. HOMO, LUMO representation, as well as geometry optimized structure of **1**[8] (level of theory: B3LYP-D3/def2SVP) (top). Calculated transitions (level of theory: TD-DFT/cam-B3LYP/6-31G(d,p)) and respective calculated UV/vis absorption spectrum compared to the experimental one.

S10.2 Strain-Viz calculations[20]

Calculations were run at the B3LYP/6-31G(d) level of theory, to compare the results with the ones obtained for the homodesmotic equation. Four fragments were used to calculate the strain, each fragment containing one pyrene unit less, truncated as indicated by the dashed red lines (Figure S84). For a detailed description how to perform calculations using Strain-Viz, see the cited literature.

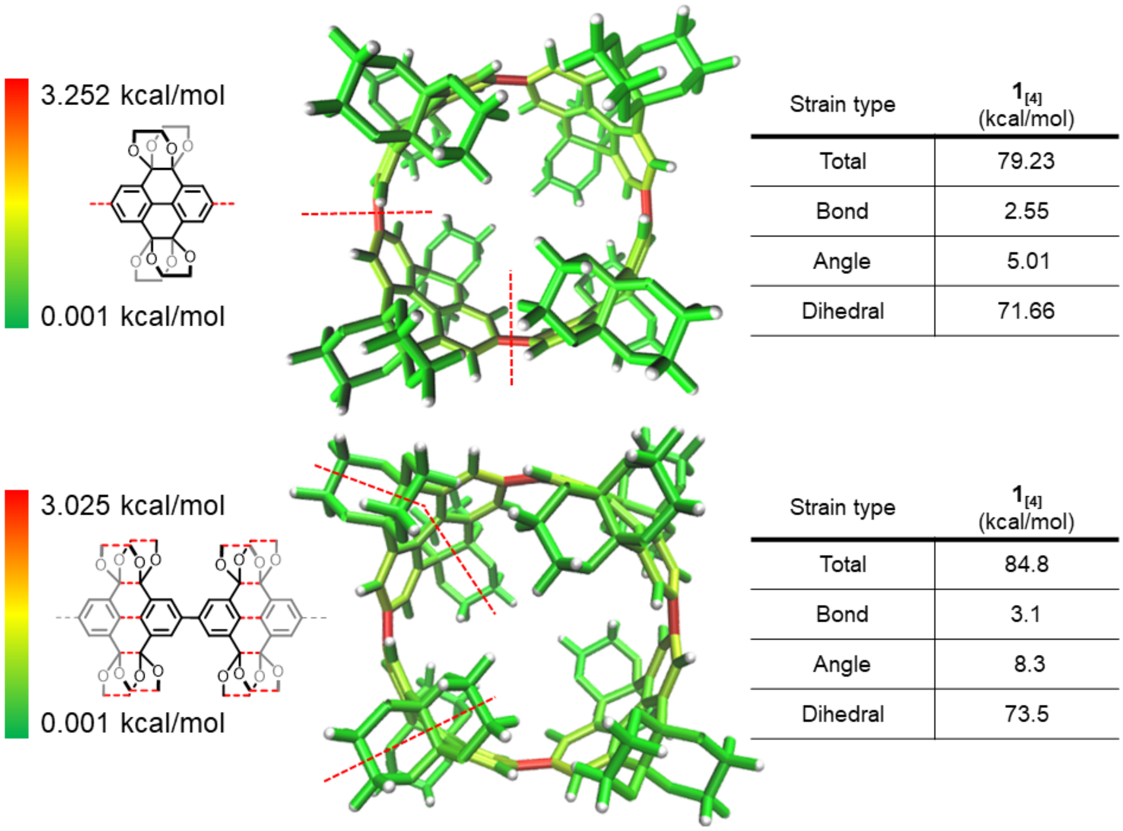

Strain type	**1**$_{[4]}$ (kcal/mol)
Total	79.23
Bond	2.55
Angle	5.01
Dihedral	71.66

Strain type	**1**$_{[4]}$ (kcal/mol)
Total	84.8
Bond	3.1
Angle	8.3
Dihedral	73.5

Figure S84. Color coded graphical representation calculated with Strain-Viz level of theory B3LYP/6-31G(d), two different ways to calculate a strain mapping trajectory with Strain-Viz are possible (top and bottom). The table states the different contributions to the total strain. Red dashed lines show the truncated bonds. The bottom strategy of fragmentation (cutting through pyrenylene) is evaluated to be unsuitable for the determination of strain parameters due to the resulting entropic freedom of residuals (alkyl) with free movement and rotation after the formal cut.

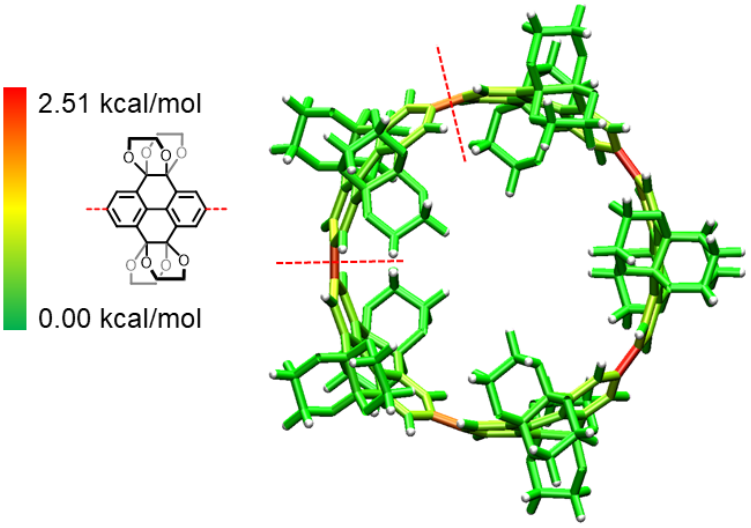

Strain type	$\mathbf{1}_{[5]}$ (kcal/mol)
Total	66.57
Bond	1.97
Angle	4.84
Dihedral	59.75

Figure S85. Color coded graphical representation of the molecular strain of $\mathbf{1}_{[5]}$ calculated with StrainViz, level of theory B3LYP/6-31G(d). The table states the different contributions to the total strain. Red dashed lines show the truncated bonds.

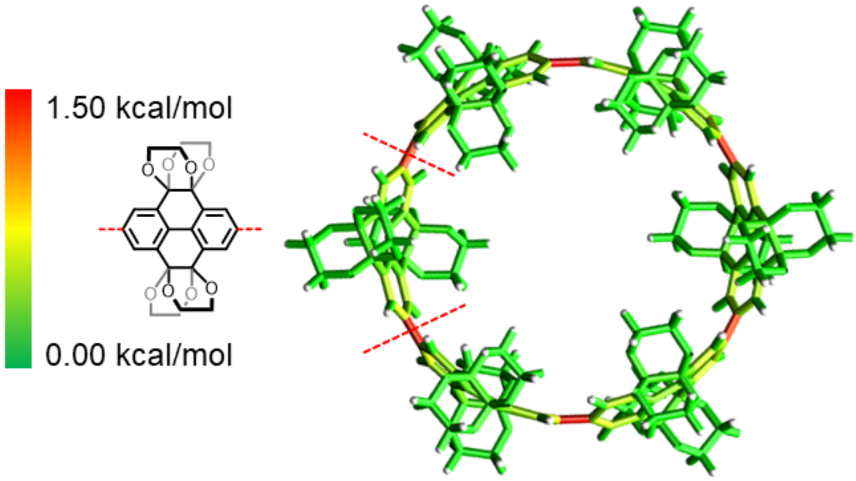

Strain type	$\mathbf{1}_{[6]}$ (kcal/mol)
Total	55.12
Bond	3.96
Angle	3.17
Dihedral	47.99

Figure S86. Color coded graphical representation of the molecular strain of $\mathbf{1}_{[6]}$ calculated with StrainViz, level of theory B3LYP/6-31G(d). The table states the different contributions to the total strain. Red dashed lines show the truncated bonds.

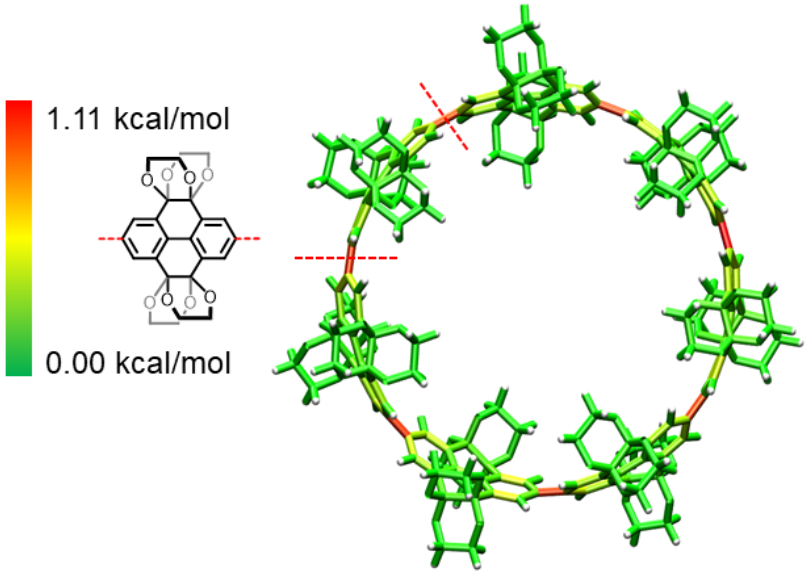

Strain type	$\mathbf{1}_{[7]}$ (kcal/mol)
Total	47.64
Bond	6.25
Angle	3.00
Dihedral	38.39

Figure S87. Color coded graphical representation of the molecular strain of $\mathbf{1}_{[7]}$ calculated with StrainViz, level of theory B3LYP/6-31G(d). The table states the different contributions to the total strain. Red dashed lines show the truncated bonds.

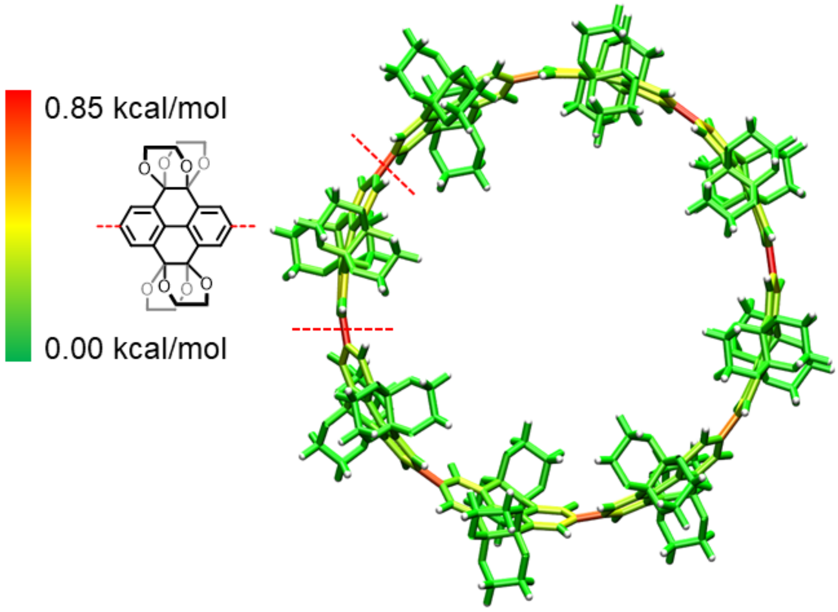

Strain type	$\mathbf{1}_{[8]}$ (kcal/mol)
Total	42.71
Bond	8.39
Angle	1.78
Dihedral	32.53

Figure S88. Color coded graphical representation of the molecular strain of $\mathbf{1}_{[8]}$ calculated with StrainViz, level of theory B3LYP/6-31G(d). The table states the different contributions to the total strain. Red dashed lines show the truncated bonds.

S10.3 Strain Calculations *via* Homodesmotic Equation[18]

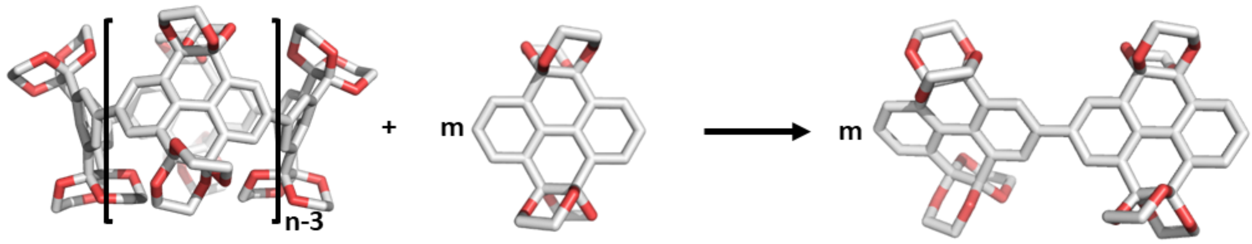

Figure S89. Homodesmotic reaction equation, with [*n*]cyclo-2,7-pyrenylenes and the monomer unit on the left, compared to the "linear" dimer on the right.

The strain energy of a molecule can be calculated using a hypothetical reaction equation that is fulfilling the prerequisites of a homodesmotic equation.[18] Energies are calculated on the DFT:B3LYP/6-31G(d) level of theory and inserted into the following equation:

$$[\Delta G(\text{cycle}) + m\ \Delta G(\text{monomer})] - \Delta G(\text{dimer}) = \text{ring strain energy}$$

Molecule	Homodesmotic equation B3LYP/6-31G(d)	StrainViz B3LYP/6-31G(d)
$\mathbf{1}_{[4]}$	87 kcal/mol	79 kcal/mol
$\mathbf{1}_{[5]}$	63 kcal/mol	67 kcal/mol
$\mathbf{1}_{[6]}$	51 kcal/mol	55 kcal/mol
$\mathbf{1}_{[7]}$	43 kcal/mol	47 kcal/mol
$\mathbf{1}_{[8]}$	37 kcal/mol	43 kcal/mol

S10.4 Atomic Coordinates of Calculated Structures

1[4] **6-31G(d)**

O	3.54746900	-5.85721800	2.89144500
O	1.37343000	-2.69614500	-2.86849100
O	0.81748300	-7.08976300	2.77148800
O	0.95687800	-4.83988700	2.93981000
O	3.08237400	-7.24520400	1.15974100
O	-0.31421100	-4.66332300	-3.87432200
O	1.08021100	-6.33547500	-3.23446100
O	2.64077900	-4.16959300	-4.02271800
C	-1.35126400	-5.14777100	-1.29398500
H	-1.98984000	-4.93514300	-2.14212800
C	-1.89998800	-5.20649400	0.00507000
C	0.01853600	-5.20994200	-1.49997700
C	4.23072500	-4.14286900	0.72500300
H	4.84450200	-4.18875200	1.61789700
C	3.12760000	-4.98252800	0.62981500
C	0.63614800	-5.04239300	-2.88133900
C	2.68687100	-4.14908700	-1.59309000
C	2.75691100	-5.98748100	1.71379300
C	3.77795200	-3.29075500	-1.47448700
H	3.95225000	-2.59142700	-2.28356400
C	2.28152300	-4.92045600	-0.49730300
C	4.50082100	-3.18335200	-0.27734200
C	1.22177500	-5.91515900	2.06597300
C	0.89007900	-5.37616200	-0.40518300
C	1.83619100	-4.02724600	-2.85033100
C	2.67179800	-8.36900300	1.94620700
H	3.18565000	-8.36061000	2.91428600
H	2.97912200	-9.25268900	1.37923300
C	-1.04208200	-5.61070700	1.04005000
H	-1.42194400	-5.75967900	2.04498100
C	0.53043500	-2.39837500	-3.98440800
H	1.07479200	-2.54842900	-4.92261900
H	0.26091600	-1.34289700	-3.88089200
C	-0.69320600	-3.28438000	-3.90801900
H	-1.27657600	-3.02254600	-3.01524700
H	-1.32863000	-3.17517200	-4.79342800
C	0.33781100	-5.68840400	0.84503100
C	3.19054000	-4.80597500	3.79095100
H	3.40278400	-3.82787100	3.33878400
H	3.83205400	-4.93567200	4.66859700
C	1.16999300	-8.31851900	2.13453600
H	0.65985700	-8.40498500	1.16478100
H	0.81415900	-9.11483900	2.79572900
C	1.72235500	-4.88749600	4.14858600
H	1.49801900	-5.80402000	4.70366800
H	1.40657100	-4.02022400	4.73635600
C	1.85028100	-6.39553200	-4.43990100
H	1.23956600	-6.08297100	-5.29481500
H	2.12982000	-7.44657300	-4.55815700
C	3.06654100	-5.50512600	-4.29404500
H	3.71317800	-5.87133800	-3.48406000
H	3.64947200	-5.45520300	-5.21909600
O	5.85730200	3.54742500	-2.89146200
O	2.69632600	1.37321400	2.86846200
O	7.08996600	0.81749800	-2.77147400
O	4.84008900	0.95678700	-2.93981000
O	7.24529300	3.08241000	-1.15974500
O	4.66362400	-0.31427100	3.87431900
O	6.33567700	1.08027600	3.23445500
O	4.16968000	2.64068600	4.02271300
C	5.14800600	-1.35129100	1.29401100
H	4.93534800	-1.98985500	2.14215800
C	5.20672500	-1.90002100	-0.00504200
C	5.21018700	0.01850700	1.49999100
C	4.14289800	4.23061800	-0.72501600
H	4.18875500	4.84440000	-1.61790800
C	4.98261400	3.12753400	-0.62982400
C	5.04262400	0.63612100	2.88134300
C	4.14919200	2.68677300	1.59308500
C	5.98758800	2.75688400	-1.71380000
C	3.29080100	3.77780400	1.47447400
H	2.59146200	3.95207100	2.28354900
C	4.92059100	2.28146500	0.49730200
C	3.18336200	4.50066300	0.27732700
C	5.91534100	1.22174100	-2.06596800
C	5.37636700	0.89004500	0.40519100
C	4.02739100	1.83608500	2.85032700
C	8.36911500	2.67187900	-1.94620300
H	8.36070300	3.18572300	-2.91428600
H	9.25278400	2.97924800	-1.37922800
C	5.61094000	-1.04212100	-1.04002600
H	5.75989200	-1.42198600	-2.04496000
C	2.39860100	0.53016400	3.98435000
H	2.54857600	1.07451300	4.92257700
H	1.34315100	0.26055300	3.88079600
C	3.28471900	-0.69339900	3.90796500
H	3.02297700	-1.27677900	3.01517000
H	3.17554400	-1.32884900	4.79335800
C	5.68861900	0.33777600	-0.84501900
C	4.80609000	3.19043600	-3.79098100
H	3.82797000	3.40264000	-3.33883200
H	4.93577100	3.83194400	-4.66863300
C	8.31870100	1.17006900	-2.13452100
H	8.40518800	0.65994600	-1.16476100
H	9.11504100	0.81426800	-2.79570800
C	4.88768100	1.72225000	-4.14859600
H	5.80422400	1.49794700	-4.70366000
H	4.02043200	1.40641800	-4.73637400
C	6.39568400	1.85035600	4.43989200

H	6.08317500	1.23962000	5.29480900
H	7.44670500	2.12997500	4.55814000
C	5.50518300	3.06654800	4.29403800
H	5.87134400	3.71321200	3.48405200
H	5.45522000	3.64947600	5.21908900
O	-5.85728600	-3.54746400	-2.89142700
O	-2.69634700	-1.37306700	2.86842200
O	-7.08996200	-0.81753700	-2.77148800
O	-4.84007800	-0.95682200	-2.93979800
O	-7.24529600	-3.08242800	-1.15972800
O	-4.66371400	0.31433800	3.87428600
O	-6.33571200	-1.08030300	3.23445200
O	-4.16964000	-2.64059800	4.02273500
C	-5.14802400	1.35129400	1.29400000
H	-4.93534200	1.98985500	2.14214400
C	-5.20672800	1.90001100	-0.00505800
C	-5.21023600	-0.01849900	1.49998700
C	-4.14289200	-4.23061500	-0.72496900
H	-4.18874300	-4.84440700	-1.61785500
C	-4.98261800	-3.12754000	-0.62979200
C	-5.04267500	-0.63609700	2.88134000
C	-4.14918900	-2.68673300	1.59310800
C	-5.98758700	-2.75690600	-1.71377800
C	-3.29078400	-3.77775700	1.47450800
H	-2.59143800	-3.95199900	2.28358100
C	-4.92060500	-2.28145700	0.49732500
C	-3.18334900	-4.50063900	0.27737300
C	-5.91534100	-1.22176800	-2.06596600
C	-5.37639800	-0.89004600	0.40519400
C	-4.02739000	-1.83601400	2.85033200
C	-8.36911200	-2.67191200	-1.94620000
H	-8.36069100	-3.18577000	-2.91427600
H	-9.25278500	-2.97927700	-1.37922800
C	-5.61094800	1.04210800	-1.04004100
H	-5.75989000	1.42196700	-2.04497800
C	-2.39863500	-0.52993900	3.98425900
H	-2.54853600	-1.07425400	4.92251700
H	-1.34320700	-0.26026200	3.88065200
C	-3.28483700	0.69356400	3.90785700
H	-3.02318300	1.27692900	3.01502500
H	-3.17567000	1.32905900	4.79321900
C	-5.68863600	-0.33778800	-0.84502500
C	-4.80606000	-3.19048600	-3.79093300
H	-3.82794700	-3.40268300	-3.33876300
H	-4.93572600	-3.83200800	-4.66857800
C	-8.31870200	-1.17010500	-2.13454000
H	-8.40519900	-0.65996800	-1.16478800
H	-9.11503800	-0.81431600	-2.79573800
C	-4.88764800	-1.72230500	-4.14857200
H	-5.80418100	-1.49801300	-4.70365600
H	-4.02038900	-1.40648200	-4.73634000
C	-6.39568600	-1.85036900	4.43990000
H	-6.08321100	-1.23960400	5.29480800
H	-7.44669500	-2.13003700	4.55814800
C	-5.50512400	-3.06651900	4.29406900
H	-5.87125200	-3.71321500	3.48409500
H	-5.45513400	-3.64942700	5.21913100
O	-3.54743100	5.85709600	2.89148900
O	-1.37341800	2.69618200	-2.86852100
O	-0.81745400	7.08967100	2.77154300
O	-0.95683300	4.83978200	2.93978700
O	-3.08236400	7.24515300	1.15982800
O	0.31418900	4.66339500	-3.87433900
O	-1.08024800	6.33552100	-3.23444100
O	-2.64079700	4.16963300	-4.02271700
C	1.35126100	5.14780100	-1.29401200
H	1.98982700	4.93518600	-2.14216500
C	1.90000100	5.20651900	0.00503800
C	-0.01854100	5.20997000	-1.49998600
C	-4.23071100	4.14283600	0.72500800
H	-4.84448100	4.18870000	1.61790900
C	-3.12758400	4.98249300	0.62983000
C	-0.63616700	5.04244000	-2.88134300
C	-2.68686800	4.14909200	-1.59309000
C	-2.75688600	5.98741300	1.71383400
C	-3.77794400	3.29075300	-1.47449400
H	-3.95224600	2.59143900	-2.28358200
C	-2.28151600	4.92044800	-0.49729500
C	-4.50081500	3.18333800	-0.27735100
C	-1.22174600	5.91508700	2.06599500
C	-0.89007500	5.37616200	-0.40518000
C	-1.83619600	4.02727700	-2.85034000
C	-2.67178500	8.36892800	1.94632500
H	-3.18562500	8.36050300	2.91440900
H	-2.97911900	9.25263200	1.37938300
C	1.04210300	5.61070000	1.04003400
H	1.42197400	5.75964600	2.04496600
C	-0.53042600	2.39843600	-3.98444500
H	-1.07479000	2.54849300	-4.92265100
H	-0.26089100	1.34296100	-3.88094100
C	0.69320400	3.28445800	-3.90805400
H	1.27658300	3.02262300	-3.01528800
H	1.32862300	3.17526900	-4.79346800
C	-0.33779500	5.68838200	0.84503500
C	-3.19048800	4.80581400	3.79094500
H	-3.40272800	3.82772800	3.33873600
H	-3.83199500	4.93546800	4.66860200
C	-1.16997600	8.31844500	2.13463500
H	-0.65985300	8.40494600	1.16487600
H	-0.81413900	9.11474700	2.79584900
C	-1.72230100	4.88733100	4.14857300
H	-1.49796800	5.80383200	4.70369300
H	-1.40650500	4.02003500	4.73630200
C	-1.85033000	6.39558900	-4.43987300
H	-1.23962000	6.08304900	-5.29479700
H	-2.12988300	7.44662900	-4.55810800

C	-3.06657900	5.50516500	-4.29401900
H	-3.71321200	5.87135600	-3.48402200
H	-3.64951700	5.45525100	-5.21906600

1[5] 6-31G(d)

O	6.92418400	-1.91080800	-3.44942900
O	5.33093900	-0.33804300	3.03512100
O	7.37973400	1.03409900	-3.68753700
O	5.33161500	0.33215900	-3.03515100
O	8.66844900	-1.01211800	-2.30971500
O	6.92612300	1.90303600	3.44955800
O	8.66943400	1.00228600	2.31000900
O	7.37816300	-1.04240400	3.68770700
C	6.41390800	2.90389300	0.85918000
H	6.33394700	3.51900900	1.74695200
C	6.00477100	3.40369700	-0.39634200
C	6.83354400	1.58960200	1.01400300
C	6.41056800	-2.91106400	-0.85909600
H	6.32997100	-3.52608700	-1.74687500
C	6.83174900	-1.59726300	-1.01388400
C	7.25883800	1.03566700	2.36809100
C	6.67099100	-1.27432400	1.37046000
C	7.25782000	-1.04383100	-2.36793300
C	6.25809500	-2.59707800	1.51144900
H	6.04770500	-2.94545900	2.51521500
C	6.87476500	-0.73004100	0.09583600
C	6.67797300	0.39532000	-2.62000500
C	6.87563200	0.72232900	-0.09571400
C	6.67726100	-0.40279700	2.62010800
C	9.29811200	-0.38404700	-3.43200900
H	9.08205400	-0.94441900	-4.34881400
H	10.37192000	-0.42299500	-3.22654300
C	6.26126600	2.59008600	-1.51137800
H	6.05136700	2.93871800	-2.51516000
C	5.12496900	0.47439600	4.19470400
H	5.68331100	0.06986500	5.04547900
H	4.05255300	0.42623600	4.40522500
C	5.56116700	1.88732600	3.87421600
H	4.91210200	2.30320100	3.09121000
H	5.51771500	2.53628400	4.75524200
C	6.67261000	1.26685300	-1.37035600
C	5.55929100	-1.89348300	-3.87422400
H	4.90965400	-2.30859500	-3.09128600
H	5.51516000	-2.54238700	-4.75525700
C	8.80067100	1.04187700	-3.54417500
H	9.09039500	1.61925000	-2.65481500
H	9.19215600	1.54179100	-4.43570100
C	5.12480000	-0.48003700	-4.19475400
H	5.68370900	-0.07616900	-5.04547100
H	4.05246300	-0.43060400	-4.40538500
C	9.29824500	0.37346600	3.43236100
H	9.08276500	0.93409000	4.34914700
H	10.37211900	0.41114100	3.22699700
C	8.79910200	-1.05186800	3.54447100
H	9.08821800	-1.62957200	2.65512900
H	9.18991600	-1.55225600	4.43602600
O	4.12137000	6.79621500	2.97553200
O	1.72708800	4.12827300	-2.86381500
O	1.41671000	8.04456800	3.07785000
O	1.52560800	5.78892800	2.95001600
O	3.63273900	8.38045500	1.42645900
O	0.04762000	6.16533100	-3.73995100
O	1.50038300	7.78346500	-3.08913800
O	2.96340100	5.61433300	-4.03885100
C	-0.89128300	6.65193600	-1.13518900
H	-1.55595200	6.55172900	-1.98443400
C	-1.41716700	6.69759900	0.17189600
C	0.47618300	6.60693900	-1.36239000
C	4.81129900	5.42148000	0.60050700
H	5.46728600	5.44237300	1.46316000
C	3.65343900	6.18968700	0.64108800
C	1.04317600	6.48656000	-2.77026600
C	3.13320700	5.51456900	-1.61481800
C	3.30869300	7.06919200	1.83697800
C	4.27483000	4.71946200	-1.62829800
H	4.44335100	4.12036300	-2.51393500
C	2.75626800	6.18592200	-0.44497500
C	5.09385000	4.57840400	-0.49695000
C	1.78434800	6.96574300	2.21719900
C	1.37499200	6.64748100	-0.27879200
C	2.21890800	5.44874600	-2.83087800
C	3.25847400	9.40459300	2.35507400
H	3.80447200	9.27794300	3.29679600
H	3.55443600	10.34814300	1.88720900
C	-0.51169700	6.92640000	1.21931100
H	-0.86494800	7.04454400	2.23799300
C	0.81770100	3.88148900	-3.94055800
H	1.31708300	4.04051300	-4.90204900
H	0.52617000	2.83113400	-3.84894500
C	-0.37509900	4.79952100	-3.78300200
H	-0.92188500	4.53851800	-2.86672900
H	-1.05695700	4.72791400	-4.63711700
C	0.86615900	6.90249100	1.00261100
C	3.78405000	5.62504400	3.72162400
H	3.97667000	4.72363200	3.12369800
H	4.45043100	5.62425400	4.59048800
C	1.76323900	9.34060500	2.58744500
H	1.22176800	9.55149500	1.65446400
H	1.43922600	10.05085200	3.35461300
C	2.32682900	5.66067500	4.12931900
H	2.12728600	6.49078800	4.81493800
H	2.02008100	4.72013600	4.59652400
C	2.21166500	7.87552800	-4.32880100
H	1.55036900	7.61588000	-5.16333400
H	2.51447900	8.92305000	-4.41705300

C	3.40885100	6.94889500	-4.28357100
H	4.10886700	7.26659100	-3.49794100
H	3.93853000	6.92053500	-5.24100000
O	-5.58307600	6.05111100	-2.76553400
O	-3.18238700	2.81521800	2.76801800
O	-7.62834800	3.88996800	-2.67519200
O	-5.45612900	3.26871200	-2.79048500
O	-7.05816100	6.07120700	-1.04161000
O	-5.53761600	1.88909500	3.92339800
O	-6.65109900	3.80520700	3.43318200
O	-4.02324600	4.44663200	4.09302500
C	-6.59595400	1.20301100	1.41507600
H	-6.61487600	0.53341500	2.26518700
C	-6.95764400	0.73612300	0.13418900
C	-6.08706100	2.47837300	1.60440200
C	-3.80466900	6.23256100	-0.57522500
H	-3.70669700	6.87923300	-1.44034000
C	-4.90012600	5.38017300	-0.50366500
C	-5.62468500	2.94959500	2.97655700
C	-4.16845900	4.63067400	1.67324700
C	-5.97252900	5.35214000	-1.58602300
C	-3.05500700	5.46100900	1.57338600
H	-2.32083100	5.41569900	2.36941500
C	-5.05594900	4.51205200	0.59523200
C	-2.80187400	6.21401400	0.41725600
C	-6.40414000	3.88101700	-1.94112200
C	-5.95608900	3.35784800	0.51268200
C	-4.25899900	3.71619600	2.88825200
C	-8.24292100	6.05879400	-1.84662900
H	-8.05184300	6.55189900	-2.80642600
H	-8.98604300	6.63222700	-1.28481200
C	-7.00391200	1.68571800	-0.89880700
H	-7.34730800	1.41219900	-1.89040800
C	-3.12083300	1.85099700	3.82523400
H	-2.98067000	2.35551500	4.78696400
H	-2.24765300	1.22882200	3.60832500
C	-4.39464200	1.03338300	3.81372500
H	-4.44826600	0.43787100	2.89308700
H	-4.45004900	0.35690700	4.67267800
C	-6.50808100	2.97737200	-0.71879600
C	-4.66950200	5.36963400	-3.62691200
H	-3.69276700	5.25822000	-3.13607600
H	-4.55275900	6.01190700	-4.50572600
C	-8.68564800	4.62558200	-2.05664700
H	-8.95807000	4.16446600	-1.09694400
H	-9.53929200	4.55849800	-2.73827100
C	-5.21050700	4.00453100	-3.99450800
H	-6.13085800	4.08733200	-4.58171900
H	-4.47262300	3.42215700	-4.55487900
C	-6.35657900	4.47870500	4.66229400
H	-6.23597000	3.75002300	5.47187700
H	-7.22594900	5.11027300	4.86768500
C	-5.09685500	5.30173500	4.48775800
H	-5.25452600	6.08653600	3.73440400
H	-4.78237300	5.76878200	5.42641300
C	6.00075500	-3.41039700	0.39639400
C	5.08850200	-4.58407600	0.49694000
C	4.80499900	-5.42676800	-0.60056700
C	4.26933800	-4.72429600	1.62828800
H	5.46094800	-5.44833500	-1.46323100
C	3.64628900	-6.19368900	-0.64118000
C	3.12683400	-5.51813500	1.61477300
H	4.43853900	-4.12544900	2.51396600
C	3.30056300	-7.07274300	-1.83711900
C	2.74913600	-6.18900000	0.44489400
C	2.21262200	-5.45137100	2.83084900
O	4.11351700	-6.80058000	-2.97567000
O	3.62319100	-8.38438200	-1.42668500
C	1.77632700	-6.96761400	-2.21730900
C	1.36735200	-6.64903300	0.27870400
O	1.72227200	-4.13035400	2.86387100
O	2.95694400	-5.61785900	4.03880500
C	1.03574000	-6.48787700	2.77018900
C	3.77746200	-5.62899400	-3.72168100
C	3.24780000	-9.40805600	-2.35535700
O	1.40750200	-8.04598500	-3.07802200
O	1.51885500	-5.79047200	-2.95004700
C	0.85822600	-6.90343200	-1.00270300
C	0.46859900	-6.60755300	1.36231300
C	0.81316700	-3.88262400	3.94063500
C	3.40091900	-6.95292900	4.28343600
O	0.04055200	-6.16559900	3.73990200
O	1.49151000	-7.78530800	3.08898000
H	3.97107500	-4.72783100	-3.12369800
H	4.44382900	-5.62887400	-4.59055600
C	2.32019700	-5.66301300	-4.12935500
H	3.79392600	-9.28194500	-3.29707700
H	3.54274000	-10.35195500	-1.88755200
C	1.75263300	-9.34242900	-2.58770500
C	-0.51965700	-6.92582500	-1.21938900
C	-0.89891800	-6.65102700	1.13512300
H	1.31237800	-4.04225500	4.90211400
H	0.52279900	-2.83194000	3.84908300
C	-0.38065200	-4.79932400	3.78303200
C	2.20270700	-7.87823700	4.32862600
H	4.10057100	-7.27135000	3.49777500
H	3.93064400	-6.92521400	5.24085800
H	2.11974200	-6.49286500	-4.81502400
H	2.01446400	-4.72211100	-4.59649400
H	1.21094500	-9.55279200	-1.65473100
H	1.42783900	-10.05227400	-3.35491500
C	-1.42486600	-6.69606000	-0.17195900
H	-0.87304500	-7.04354500	-2.23807300
H	-1.56346700	-6.55012100	1.98437900
H	-0.92715200	-4.53766300	2.86677700
H	-1.06242500	-4.72700900	4.63715500

H	1.54171200	-7.61790900	5.16318600
H	2.50436100	-8.92610000	4.41680700
C	-2.80903500	-6.21092200	-0.41729500
C	-3.81182600	-6.22831900	0.57521400
C	-3.06134700	-5.45764400	-1.57342600
H	-3.71456600	-6.87509200	1.44033200
C	-4.90631100	-5.37468100	0.50367800
C	-4.17385500	-4.62604000	-1.67326300
H	-2.32714400	-5.41317500	-2.36947700
C	-5.97864600	-5.34541300	1.58607200
C	-5.06117500	-4.50639100	-0.59522200
C	-4.26337700	-3.71146400	-2.88827000
O	-5.58995600	-6.04481800	2.76557600
O	-7.06511400	-6.06324600	1.04169800
C	-6.40856700	-3.87379400	1.94117000
C	-5.95998500	-3.35115300	-0.51265100
O	-3.18571800	-2.81173100	-2.76807400
O	-4.02850500	-4.44217700	-4.09304600
C	-5.62817800	-2.94328300	-2.97653500
C	-4.67558400	-5.36437900	3.62692900
C	-8.24983500	-6.04947100	1.84675300
O	-7.63276500	-3.88133900	2.67527000
O	-5.45983200	-3.26256700	2.79050100
C	-6.51150300	-2.97003700	0.71884200
C	-6.08997200	-2.47152900	-1.60436700
C	-3.12308300	-1.84758000	-3.82529000
C	-5.10311700	-5.29603100	-4.48775500
O	-5.53990700	-1.88288300	-3.92337700
O	-6.65559400	-3.79770400	-3.43313500
H	-3.69873300	-5.25408800	3.13607300
H	-4.55955800	-6.00677700	4.50574600
C	-5.21501800	-3.99865400	3.99452500
H	-8.05928900	-6.54277600	2.80655300
H	-8.99362800	-6.62206600	1.28496800
C	-8.69092100	-4.61575200	2.05676100
C	-7.00583900	-1.67781200	0.89886400
C	-6.59738600	-1.19557900	-1.41502900
H	-2.98353700	-2.35225600	-4.78702700
H	-2.24917500	-1.22641800	-3.60840800
C	-4.39594300	-1.02849300	-3.81373400
C	-6.36188800	-4.47153800	-4.66225800
H	-5.26168100	-6.08065200	-3.73440100
H	-4.78920000	-5.76343900	-5.42642000
H	-6.13544800	-4.08039400	4.58176200
H	-4.47645400	-3.41711800	4.55487000
H	-8.96283900	-4.15434000	1.09705800
H	-9.54447100	-4.54768200	2.73840500
C	-6.95849800	-0.72827100	-0.13413200
H	-7.34889200	-1.40389600	1.89047400
H	-6.61555800	-0.52596400	-2.26514100
H	-4.44884800	-0.43292800	-2.89308900
H	-4.45059600	-0.35194600	-4.67267900
H	-6.24045300	-3.74299200	-5.47184000
H	-7.23199600	-5.10209500	-4.86762900

1[6] 6-31G(d)

O	-7.93897800	1.42797000	3.98097300
O	-6.90180500	1.37219900	-2.77006100
O	-7.26977300	4.30623800	3.68394100
O	-5.76982700	2.80065900	2.90406600
O	-9.43925400	2.74582000	2.90151700
O	-7.85988700	3.88387800	-3.49969700
O	-9.60766900	3.74832400	-2.05841000
O	-9.13034700	1.22381700	-3.13169600
C	-6.75544700	5.11951200	-1.21996100
H	-6.71221500	5.58233400	-2.19934200
C	-6.01357900	5.65233700	-0.14531600
C	-7.50581300	3.96028900	-1.07235000
C	-8.33320100	-0.05752100	1.62810800
H	-8.41174700	-0.50366000	2.61246800
C	-8.18967700	1.31981700	1.54330000
C	-8.26666700	3.34947400	-2.24232800
C	-8.25054900	1.16770900	-0.86548200
C	-8.14713200	2.18815200	2.79402900
C	-8.33891100	-0.21986500	-0.76614200
H	-8.33983500	-0.79023500	-1.68789300
C	-8.08247500	1.94715800	0.28737000
C	-7.06104300	3.31630600	2.67554700
C	-7.59318100	3.32454800	0.18103100
C	-8.14975900	1.78217700	-2.25568800
C	-9.58152800	3.70819300	3.95290500
H	-9.41434600	3.23125000	4.92519100
H	-10.61589700	4.05932100	3.89272800
C	-6.22151900	5.08574500	1.12146600
H	-5.68676000	5.45993100	1.98678200
C	-6.62294600	1.88991400	-4.07583900
H	-7.36882000	1.53031100	-4.79250100
H	-5.63797100	1.49801500	-4.34695200
C	-6.61446400	3.40232300	-4.00873000
H	-5.78593400	3.73836200	-3.37006900
H	-6.50320100	3.85341800	-5.00000800
C	-7.01278900	3.95184700	1.29181700
C	-6.60195300	0.96787300	4.20247400
H	-6.32155300	0.22636500	3.44237200
H	-6.61392300	0.48168400	5.18312800
C	-8.59528300	4.83633100	3.73049100
H	-8.82575300	5.36653800	2.79562700
H	-8.60513200	5.55388100	4.55700400
C	-5.63319400	2.13036400	4.16256800
H	-5.81853400	2.82998800	4.98433500
H	-4.59611300	1.78497300	4.20362700
C	-10.53029400	3.17180300	-2.99029100
H	-10.29214900	3.50004000	-4.00828700
H	-11.51441600	3.55524100	-2.70537500
C	-10.46736600	1.66214800	-2.88270900

H	-10.79447200	1.33560500	-1.88560400
H	-11.09108400	1.17427100	-3.63833800
O	-2.79692600	6.19458700	-3.98514100
O	-2.11809300	7.39633500	2.67645300
O	0.03008500	7.09735100	-4.08225900
O	-0.47805600	5.40608200	-2.66546300
O	-2.38605300	8.39495300	-3.60690400
O	-0.36431700	9.55840000	2.70931300
O	-1.38341300	10.54336500	0.93696600
O	-3.31682800	9.30981200	2.51517400
C	1.17508600	8.56075200	0.56767400
H	1.63820700	9.02207800	1.43332000
C	1.96607400	7.90508900	-0.39677400
C	-0.21102700	8.58823700	0.46112500
C	-4.20488900	6.60033500	-1.59422500
H	-4.63838600	6.11004300	-2.45690500
C	-2.93311600	7.13848900	-1.72407900
C	-1.07458200	9.28799500	1.50359000
C	-3.02394600	7.87049300	0.57431300
C	-2.20483800	7.10607300	-3.06114700
C	-4.29495200	7.30973500	0.69595300
H	-4.79575700	7.39317700	1.65420000
C	-2.29494800	7.72550100	-0.61533300
C	-4.88010700	6.59051100	-0.35777500
C	-0.68140100	6.78381000	-2.88460800
C	-0.85290800	7.99095000	-0.64140100
C	-2.38677700	8.48173000	1.81655700
C	-1.66547100	8.62993900	-4.82246400
H	-2.02500700	7.95749400	-5.60951300
H	-1.88577300	9.66501300	-5.09971700
C	1.32227300	7.43290400	-1.54962700
H	1.88662600	6.91986200	-2.31996500
C	-1.47925500	7.77746800	3.90024900
H	-2.12769500	8.44999300	4.47148800
H	-1.33342600	6.84927200	4.46077300
C	-0.15729800	8.43930700	3.57394600
H	0.51465200	7.71055800	3.09945700
H	0.32991300	8.83975400	4.46870800
C	-0.06260200	7.48204500	-1.68130400
C	-2.52122300	4.80918300	-3.75917100
H	-2.99181200	4.47359300	-2.82488800
H	-2.97764600	4.27504100	-4.59920700
C	-0.18623700	8.42174600	-4.57134500
H	0.18596500	9.15994800	-3.84702100
H	0.39831300	8.50310800	-5.49314400
C	-1.02742400	4.57347400	-3.69208900
H	-0.54829200	4.79171200	-4.65228500
H	-0.79944000	3.54566000	-3.39519900
C	-2.29972400	11.33076600	1.70665500
H	-1.85959000	11.57761200	2.67931200
H	-2.45247900	12.24943100	1.13265400
C	-3.59199200	10.56006400	1.88083500
H	-4.07277300	10.39394900	0.90653400
H	-4.29298400	11.08444900	2.53783100
O	4.18907800	6.07462700	3.73289900
O	5.40456600	5.09236800	-2.88885200
O	6.40546200	4.12149500	4.06402000
O	4.65097100	3.50105600	2.77664700
O	6.28371200	6.76186800	3.19096800
O	8.15821700	4.68193600	-2.92677200
O	8.52602500	6.31527400	-1.39408100
O	6.43715900	7.09951500	-3.05968600
C	8.13124600	3.19311600	-0.53770100
H	8.74549900	2.90898500	-1.38457700
C	7.97587800	2.31864200	0.55720600
C	7.45634000	4.40655000	-0.59277900
C	3.75245800	7.11328300	1.16363200
H	3.12304000	7.36800200	2.00799900
C	4.87235500	6.32522700	1.38983700
C	7.59254300	5.33457900	-1.79371300
C	5.39864500	6.41915100	-0.96647800
C	5.24959900	5.88526000	2.79826500
C	4.25678600	7.18899300	-1.18348000
H	4.03787700	7.50410000	-2.19808400
C	5.67624000	5.91053100	0.31096900
C	3.36645400	7.48117800	-0.13961100
C	5.73995800	4.39573900	2.82981000
C	6.63942900	4.81796400	0.47812300
C	6.22018100	5.99674200	-2.17865600
C	6.88419000	6.44324900	4.45167700
H	6.14352600	6.52991200	5.25469100
H	7.67063500	7.18906100	4.60001900
C	7.27009900	2.80560800	1.66755500
H	7.12805100	2.18721700	2.54547300
C	6.02841100	4.55007600	-4.05877700
H	6.25970700	5.35132700	-4.76856400
H	5.29198000	3.87240900	-4.50036300
C	7.28092600	3.80818000	-3.64388800
H	7.01156200	2.94311100	-3.02294000
H	7.85207000	3.45841700	-4.50974300
C	6.62223900	4.03694700	1.64159200
C	3.12811100	5.11676500	3.67430700
H	2.57898600	5.21128000	2.72782900
H	2.45583600	5.36691200	4.50165000
C	7.45005900	5.03958600	4.39131600
H	8.25348200	4.98308300	3.64348000
H	7.84369700	4.71740800	5.36050500
C	3.67994200	3.71366900	3.80727200
H	4.13415100	3.55945800	4.79193100
H	2.90142500	2.96323000	3.64158600
C	8.71302700	7.37436800	-2.34023000
H	9.12626400	6.97688800	-3.27404700
H	9.43746700	8.05404800	-1.88192000
C	7.38469700	8.05888400	-2.58798800
H	7.02006800	8.53103100	-1.66481200
H	7.45917700	8.82044400	-3.37063900

C	-8.30105200	-0.87051100	0.47647500
C	-7.97587300	-2.31863100	0.55733200
C	-8.13125300	-3.19312600	-0.53755600
C	-7.27008200	-2.80557600	1.66768300
H	-8.74551500	-2.90901200	-1.38443100
C	-7.45634700	-4.40656100	-0.59261800
C	-6.62222200	-4.03691500	1.64173700
H	-7.12802500	-2.18716800	2.54558800
C	-7.59256200	-5.33461400	-1.79353200
C	-6.63942400	-4.81795400	0.47828300
C	-5.73992800	-4.39568500	2.82995300
O	-8.15824900	-4.68199300	-2.92659700
O	-8.52603900	-6.31530200	-1.39387100
C	-6.22020400	-5.99678300	-2.17847700
C	-5.67623700	-5.91052500	0.31114000
O	-4.65094200	-3.50100200	2.77676100
O	-6.40541900	-4.12141700	4.06416400
C	-5.24956900	-5.88520500	2.79843100
C	-7.28096600	-3.80825000	-3.64374000
C	-8.71305100	-7.37441400	-2.33999800
O	-6.43719100	-7.09957300	-3.05948400
O	-5.40459700	-5.09242200	-2.88869900
C	-5.39865500	-6.41916900	-0.96630100
C	-4.87234000	-6.32520000	1.39000700
C	-3.67990300	-3.71359500	3.80738000
C	-7.45001300	-5.03950200	4.39148900
O	-4.18903900	-6.07455500	3.73305700
O	-6.28367800	-6.76180700	3.19116100
H	-7.01159600	-2.94316900	-3.02281200
H	-7.85211900	-3.45850400	-4.50959600
C	-6.02845500	-4.55015400	-4.05862800
H	-9.12629800	-6.97695100	-3.27381800
H	-9.43748500	-8.05408500	-1.88166800
C	-7.38472300	-8.05893400	-2.58775800
C	-4.25679800	-7.18901500	-1.18330000
C	-3.75244600	-7.11325900	1.16380500
H	-4.13410100	-3.55936500	4.79204100
H	-2.90138700	-2.96315900	3.64167200
C	-3.12807300	-5.11669400	3.67443700
C	-6.88414200	-6.44316300	4.45187200
H	-8.25344400	-4.98301400	3.64366100
H	-7.84364000	-4.71730500	5.36067600
H	-6.25975800	-5.35141800	-4.76839800
H	-5.29202900	-3.87249500	-4.50023600
H	-7.02008400	-8.53106300	-1.66457600
H	-7.45921100	-8.82050900	-3.37039300
C	-3.36645500	-7.48117900	-0.13943400
H	-4.03789900	-7.50414100	-2.19790000
H	-3.12301800	-7.36796200	2.00817100
H	-2.57895700	-5.21122700	2.72795500
H	-2.45578900	-5.36682500	4.50177700
H	-6.14347000	-6.52981100	5.25487900
H	-7.67058500	-7.18897300	4.60023600
C	-1.96607800	-7.90509500	-0.39660400
C	-1.17508000	-8.56074000	0.56784900
C	-1.32228800	-7.43293200	-1.54947200
H	-1.63819200	-9.02204900	1.43350800
C	0.21103200	-8.58822800	0.46128500
C	0.06258500	-7.48207600	-1.68116300
H	-1.88664900	-6.91990500	-2.31981400
C	1.07459700	-9.28796600	1.50375500
C	0.85290200	-7.99096200	-0.64125900
C	0.68137200	-6.78386500	-2.88448600
O	0.36434500	-9.55834800	2.70949000
O	1.38342200	-10.54334800	0.93715100
C	2.38679600	-8.48169600	1.81669200
C	2.29494200	-7.72551300	-0.61521100
O	0.47802900	-5.40613200	-2.66536500
O	-0.03012700	-7.09742700	-4.08212400
C	2.20480700	-7.10613100	-3.06103500
C	0.15733600	-8.43923900	3.57410500
C	2.29974000	-11.33073500	1.70684500
O	3.31685400	-9.30976500	2.51531500
O	2.11812200	-7.39628500	2.67657100
C	3.02395200	-7.87048400	0.57443100
C	2.93309900	-7.13852300	-1.72397400
C	1.02738700	-4.57354400	-3.69201300
C	0.18619000	-8.42183200	-4.57118700
O	2.79688500	-6.19466300	-3.98505300
O	2.38601600	-8.39502100	-3.60677100
H	-0.51461800	-7.71049800	3.09960900
H	-0.32986600	-8.83966800	4.46887900
C	1.47929700	-7.77739500	3.90038100
H	1.85961700	-11.57756100	2.67951200
H	2.45248800	-12.24941000	1.13286000
C	3.59201100	-10.56003000	1.88099700
C	4.29496000	-7.30972300	0.69604600
C	4.20487300	-6.60036600	-1.59414400
H	0.54824600	-4.79180000	-4.65220000
H	0.79940700	-3.54572400	-3.39514000
C	2.52118600	-4.80925500	-3.75910600
C	1.66542100	-8.63003000	-4.82231800
H	-0.18600500	-9.16002000	-3.84684600
H	-0.39837000	-8.50321100	-5.49297800
H	2.12774300	-8.44991000	4.47162500
H	1.33347500	-6.84918800	4.46088900
H	4.07278200	-10.39393400	0.90668700
H	4.29301000	-11.08440300	2.53799500
C	4.88010400	-6.59051900	-0.35770100
H	4.79577500	-7.39314700	1.65429000
H	4.63836200	-6.11009000	-2.45683700
H	2.99178500	-4.47364700	-2.82483400
H	2.97760000	-4.27512800	-4.59915700
H	2.02494900	-7.95760000	-5.60938300
H	1.88571900	-9.66511000	-5.09955400
C	6.01357800	-5.65234000	-0.14527100

C	6.75543500	-5.11953600	-1.21993300
C	6.22153000	-5.08572400	1.12149800
H	6.71219400	-5.58237700	-2.19930600
C	7.50580200	-3.96031000	-1.07235200
C	7.01280200	-3.95182300	1.29181900
H	5.68677900	-5.45989400	1.98682600
C	8.26664500	-3.34951700	-2.24234900
C	7.59318300	-3.32454500	0.18101600
C	7.06106900	-3.31625500	2.67553700
O	7.85985300	-3.88394500	-3.49970400
O	9.60764900	-3.74836400	-2.05843700
C	8.14973600	-1.78222000	-2.25573800
C	8.08247700	-1.94715300	0.28732400
O	5.76985500	-2.80060400	2.90405800
O	7.26980800	-4.30616800	3.68394800
C	8.14715900	-2.18809800	2.79398700
C	6.61442500	-3.40240000	-4.00873400
C	10.53026500	-3.17186000	-2.99033800
O	9.13031700	-1.22387700	-3.13176600
O	6.90177800	-1.37225200	-2.77010700
C	8.25054000	-1.16772500	-0.86554500
C	8.18969100	-1.31978800	1.54324000
C	5.63323400	-2.13028400	4.16254800
C	8.59531800	-4.83626000	3.73049500
O	7.93901600	-1.42789400	3.98091800
O	9.43928100	-2.74576400	2.90147400
H	5.78590200	-3.73842700	-3.37005800
H	6.50315200	-3.85351400	-5.00000200
C	6.62290700	-1.88999300	-4.07587300
H	10.29211000	-3.50011700	-4.00832500
H	11.51439000	-3.55529300	-2.70542400
C	10.46733800	-1.66220300	-2.88278300
C	8.33890300	0.21985000	-0.76623200
C	8.33321700	0.05755100	1.62802100
H	5.81858200	-2.82989300	4.98432700
H	4.59615300	-1.78489200	4.20361100
C	6.60199300	-0.96779300	4.20242300
C	9.58156500	-3.70811800	3.95287800
H	8.82578000	-5.36648500	2.79564000
H	8.60517500	-5.55379400	4.55702200
H	7.36877400	-1.53040300	-4.79254900
H	5.63792900	-1.49809900	-4.34698300
H	10.79445300	-1.33564100	-1.88568800
H	11.09104900	-1.17434100	-3.63842800
C	8.30105500	0.87052000	0.47637300
H	8.33981800	0.79020200	-1.68799400
H	8.41177200	0.50370900	2.61237100
H	6.32158600	-0.22630000	3.44231000
H	6.61397300	-0.48158500	5.18306800
H	9.41439300	-3.23115600	4.92515700
H	10.61593400	-4.05924600	3.89269800

1[7] 6-31G(d)

O	-2.99881400	-11.03076600	2.35634600
O	-3.13217300	-9.46280900	-3.73724500
O	-5.60730900	-9.63503600	2.53679500
O	-5.25121900	-10.95077500	0.72233600
O	-2.95768600	-8.77920600	2.59153000
O	-4.40571100	-7.01530600	-4.10845100
O	-3.09296700	-5.98266900	-2.57832900
O	-1.56288800	-7.84017700	-3.97587300
C	-5.77674100	-6.77448800	-1.61811400
H	-5.96421500	-6.00971900	-2.36360000
C	-6.61878800	-6.89596500	-0.50341100
C	-4.62029400	-7.53964700	-1.74145500
C	-1.05106700	-9.87221300	0.60419000
H	-0.70320100	-10.27894400	1.54752700
C	-2.41277300	-9.60427400	0.47232700
C	-3.67946600	-7.22424500	-2.89702000
C	-2.03065200	-8.83239200	-1.78331600
C	-3.30700900	-9.80727100	1.68924500
C	-0.67986900	-9.11804800	-1.64463000
H	-0.02660400	-8.91095300	-2.48340100
C	-2.91007900	-9.01547600	-0.70001500
C	-0.14396400	-9.56653000	-0.42207600
C	-4.83749400	-9.75257000	1.34271000
C	-4.26028000	-8.44333100	-0.73129500
C	-2.58399700	-8.32381800	-3.10821900
C	-3.73092400	-8.78746500	3.79748600
H	-3.56756000	-9.72215200	4.34406200
H	-3.36126400	-7.94768900	4.39364000
C	-6.32186800	-7.90404300	0.43352000
H	-6.98995500	-8.08801100	1.26816700
C	-3.81906000	-9.18435400	-4.96314900
H	-3.11933800	-8.78046700	-5.70331900
H	-4.20178600	-10.14807800	-5.31189000
C	-4.94161800	-8.20380700	-4.69212700
H	-5.68743300	-8.65343400	-4.02146600
H	-5.43859300	-7.88953400	-5.61534500
C	-5.15587700	-8.65519600	0.33468000
C	-3.43664000	-12.20650600	1.67216300
H	-2.91750800	-12.29960800	0.70798800
H	-3.15453500	-13.04979500	2.31025500
C	-5.19223800	-8.61077400	3.44267100
H	-5.34863900	-7.61959600	2.99452900
H	-5.83882000	-8.70809500	4.32057600
C	-4.93488700	-12.13764800	1.45982800
H	-5.46480800	-12.13747000	2.41887300
H	-5.29315100	-12.96950600	0.84633300
C	-2.13739400	-5.53278500	-3.54674900
H	-2.62594500	-5.38203100	-4.51509000
H	-1.76502900	-4.57458600	-3.17293100
C	-1.02515100	-6.55487000	-3.64728000
H	-0.47464700	-6.60368300	-2.69837000
H	-0.32213700	-6.31300400	-4.45055500

O	-7.72175200	-4.23730000	3.62402300
O	-8.34330700	-2.69186500	-2.94136500
O	-8.88515800	-1.53588800	4.00493400
O	-7.04940000	-1.70160000	2.69058500
O	-9.91319800	-3.95977800	3.10036800
O	-10.67669800	-1.17310900	-2.99847100
O	-11.72646200	-2.54497600	-1.52585200
O	-10.11612900	-4.07637700	-3.20361900
C	-10.12488300	0.10272000	-0.55371600
H	-10.57629900	0.62804000	-1.38756300
C	-9.65350500	0.81315700	0.56835300
C	-9.97703000	-1.27561500	-0.65122100
C	-7.81714200	-5.35123800	1.05336300
H	-7.38074000	-5.87540600	1.89562200
C	-8.45917000	-4.14281200	1.28752600
C	-10.46072100	-2.03221400	-1.88234100
C	-8.94450100	-3.95856300	-1.07300700
C	-8.60719000	-3.60188300	2.70355200
C	-8.26819500	-5.15634600	-1.29888900
H	-8.20121600	-5.52428900	-2.31724200
C	-8.98584400	-3.40092300	0.21384000
C	-7.63398000	-5.84202600	-0.25269500
C	-8.41530200	-2.04677800	2.75646600
C	-9.40117700	-2.00783200	0.40399500
C	-9.48082700	-3.19441900	-2.27756000
C	-10.30794600	-3.43726900	4.37446800
H	-9.66895700	-3.84793500	5.16429700
H	-11.33695700	-3.77632500	4.52575900
C	-9.18652000	0.05591600	1.65347300
H	-8.81856100	0.54190400	2.54888700
C	-8.65157100	-1.90085600	-4.09536600
H	-9.17304200	-2.51002200	-4.84123000
H	-7.68775400	-1.57640500	-4.49827800
C	-9.49609400	-0.71935700	-3.66755900
H	-8.91214700	-0.06312900	-3.00805800
H	-9.84559600	-0.13762400	-4.52641400
C	-9.07555700	-1.32943000	1.58665800
C	-6.35330400	-3.82704900	3.55058500
H	-5.91247000	-4.13738000	2.59364000
H	-5.84122600	-4.35140900	4.36410000
C	-10.21813600	-1.92553100	4.34139400
H	-10.92915100	-1.51630300	3.61010700
H	-10.42393900	-1.48448000	5.32178400
C	-6.24838800	-2.32439200	3.70108100
H	-6.57789800	-2.00338800	4.69507700
H	-5.22586200	-1.97757600	3.52520000
C	-12.31668700	-3.39826800	-2.51364500
H	-12.50613400	-2.83425400	-3.43376000
H	-13.26822000	-3.72868500	-2.08677500
C	-11.38944500	-4.56628900	-2.77947000
H	-11.27807700	-5.17950300	-1.87416200
H	-11.75625800	-5.19918600	-3.59369700
C	2.27785500	9.67599900	0.56200900
C	2.15528800	8.67592500	-1.61728100
H	2.84363300	10.03480600	1.41482000
C	0.89706800	9.84099900	0.56776700
C	0.77764600	8.86601700	-1.63829400
H	2.60585500	8.17019600	-2.46306500
C	0.19718900	10.55063600	1.72075300
C	0.12008800	9.39237900	-0.51723800
C	0.00324500	8.31215400	-2.82648300
O	1.00661300	10.61079600	2.89190000
O	0.03209800	11.88288000	1.28431200
C	-1.19184800	9.89996200	2.05801400
C	-1.34085400	9.32226100	-0.41792600
O	0.02350400	6.91066500	-2.66941300
O	0.68411100	8.57537400	-4.05445300
C	-1.46608900	8.85036600	-2.89857000
C	1.11776000	9.38886900	3.62776600
C	-0.71478500	12.70983300	2.18464800
O	-1.95146000	10.77406600	2.89327300
O	-1.02520100	8.70954700	2.79450700
C	-1.98191700	9.50038100	0.81720300
C	-2.11416000	8.91871300	-1.52196200
C	-0.70289200	6.20603100	-3.68222100
C	0.62993500	9.93756900	-4.48264200
O	-2.22837900	8.07342400	-3.82104300
O	-1.49214900	10.17562800	-3.38265200
H	1.65323300	8.63450500	3.03531100
H	1.71122500	9.62841400	4.51578100
C	-0.25829600	8.87413200	3.99336000
H	-0.18308100	12.80428900	3.13802500
H	-0.77520900	13.69012600	1.70278500
C	-2.08802300	12.10494000	2.39182700
C	-3.32530700	9.16138700	0.97084500
C	-3.45483700	8.60137600	-1.35873700
H	-0.25550600	6.38988100	-4.66496500
H	-0.61004600	5.14542200	-3.43086700
C	-2.14798700	6.65590500	-3.64888900
C	-0.81644900	10.36201500	-4.63190300
H	1.14499600	10.58382600	-3.75816000
H	1.16539900	9.97652000	-5.43652900
H	-0.76876400	9.56220700	4.67555500
H	-0.20443800	7.88122000	4.44914300
H	-2.64950200	12.09903900	1.44696500
H	-2.66434000	12.65344400	3.14355900
C	-4.07485400	8.63949900	-0.09512500
H	-3.76443900	9.26703500	1.95667200
H	-4.00103100	8.25020900	-2.22537500
H	-2.60809400	6.36043400	-2.69607400
H	-2.72531300	6.22260600	-4.47231300
H	-1.31294600	9.78313100	-5.41876300
H	-0.90177500	11.42923900	-4.85650800
C	-5.37726800	7.95440400	0.11098800
C	-6.25134800	7.67861700	-0.95987600
C	-5.68083700	7.37746100	1.35371300

H	-6.11945300	8.17004600	-1.91710800
C	-7.26574400	6.73769400	-0.83956700
C	-6.72876600	6.47162100	1.49999700
H	-5.04807500	7.55843600	2.21493200
C	-8.18237200	6.41120200	-2.01180800
C	-7.48472500	6.08094900	0.38590800
C	-6.90416100	5.78943100	2.85089200
O	-7.65580300	6.86203100	-3.25772500
O	-9.35625300	7.16015200	-1.78444800
C	-8.50786900	4.87656600	-2.08682400
C	-8.34241800	4.89488200	0.45592700
O	-5.81125200	4.90936600	2.97884700
O	-6.77732600	6.72929100	3.91863800
C	-8.26517400	5.01728100	2.97328700
C	-6.59220800	6.07767600	-3.80155900
C	-10.41013300	6.90566400	-2.72095600
O	-9.61037900	4.65203800	-2.96666600
O	-7.42658300	4.15640900	-2.63652100
C	-8.76702400	4.25849500	-0.71892500
C	-8.60489800	4.27067100	1.68964900
C	-5.82645500	4.14594900	4.19110700
C	-7.88823200	7.61521600	4.06553200
O	-8.24186900	4.16043400	4.11159900
O	-9.33458600	5.91588100	3.17492800
H	-5.70134100	6.14827500	-3.16208200
H	-6.36437400	6.51935000	-4.77700400
C	-7.01810200	4.62989100	-3.92500000
H	-10.09738700	7.20022600	-3.72893800
H	-11.24621700	7.53444200	-2.40113200
C	-10.77027900	5.43490800	-2.67689600
C	-9.28779700	2.96713100	-0.66830200
C	-9.17645800	3.00668400	1.72831400
H	-5.76331500	4.81320400	5.05707600
H	-4.93607400	3.51172300	4.15318000
C	-7.09226800	3.31622600	4.23014800
C	-9.15276000	6.80899300	4.28024000
H	-7.98750000	8.25119600	3.17457600
H	-7.65829100	8.24762900	4.92882800
H	-7.83600800	4.51938100	-4.64483600
H	-6.18056500	3.99241800	-4.22391200
H	-11.17059700	5.16860400	-1.68867900
H	-11.50929000	5.17440400	-3.44110600
C	-9.44216400	2.28394300	0.54782100
H	-9.49386100	2.47218500	-1.61023500
H	-9.36866700	2.56458800	2.69872600
H	-7.07694700	2.57622000	3.41863400
H	-7.20539600	2.79211700	5.18444300
H	-9.09901600	6.24270900	5.21674100
H	-10.04059900	7.44808400	4.29164200
C	2.93050700	9.02212400	-0.50081900
C	4.32314600	8.51755600	-0.40086600
C	4.85403100	8.13693000	0.84040400
C	5.07563100	8.20653300	-1.55039300
C	6.01361900	7.36905500	0.93034000
H	4.32143900	8.36050100	1.75805900
C	6.20458200	7.40246300	-1.47809300
H	4.75543800	8.55779800	-2.52461200
C	6.44334600	6.87191300	2.30502300
C	6.66002300	6.92833700	-0.23340300
C	6.98715300	7.03567300	-2.73263400
C	7.89490700	6.27275200	2.31289700
O	6.33687000	7.90963000	3.28104700
O	5.49246100	5.89855900	2.67287700
C	7.66301100	5.86247400	-0.16352100
C	7.49378900	5.55104800	-2.68827900
O	6.24079200	7.26345800	-3.92529700
O	8.07456500	7.93434700	-2.76749700
C	8.16208300	5.42319200	1.07660500
O	8.12227700	5.55702000	3.52388300
O	8.86055000	7.30118800	2.27266800
C	7.33895900	8.92327100	3.18115600
C	5.75883800	5.28128900	3.93816500
C	8.01309000	5.14413200	-1.31528200
O	6.45030100	4.65007600	-2.98471500
O	8.48564300	5.34131600	-3.69461100
C	5.21855900	6.30358400	-4.20816400
C	9.01441500	7.68306200	-3.81910000
C	8.89849300	4.24911200	1.16196100
C	7.11308700	4.60698700	3.87975700
C	8.71056600	8.29084200	3.29747500
H	7.24451100	9.45763400	2.22539200
H	7.14374100	9.62326700	3.99971800
H	5.72480300	6.02983300	4.73695400
H	4.95733800	4.55176600	4.08650700
C	8.70662800	3.94172300	-1.20836000
C	5.80527000	4.90832700	-4.23765400
C	9.56355900	6.27910100	-3.67024700
H	4.42470400	6.35643700	-3.45095300
H	4.80122500	6.58942300	-5.17887100
H	8.53145500	7.81100800	-4.79431300
H	9.79842500	8.43657800	-3.69958000
H	9.26693100	3.95250900	2.13670500
C	9.10982300	3.42929900	0.03476500
H	7.08914100	3.78468100	3.15210700
H	7.41034600	4.20799500	4.85473200
H	8.84654100	7.83453000	4.28433600
H	9.50631200	9.02053200	3.12103600
H	8.85565900	3.37253300	-2.11788800
H	6.52061100	4.79803500	-5.05967300
H	5.02220700	4.15001300	-4.33005000
H	10.12602700	6.18633500	-2.73064300
H	10.21873500	6.00838900	-4.50421700
C	9.53035000	2.00891300	0.16533800
C	9.85525800	1.22395700	-0.95969000
C	9.41626200	1.34119400	1.39488900
C	9.87856200	-0.16270400	-0.89378900

H	10.04619000	1.68827800	-1.91976600
C	9.49080500	-0.04619900	1.48489000
H	9.18675000	1.88581600	2.30275600
C	10.17690500	-1.00164200	-2.13015200
C	9.64510500	-0.82165300	0.32734400
C	9.21699300	-0.69236800	2.83693500
C	9.29056000	-2.29690700	-2.18212500
O	10.03149700	-0.26063000	-3.33855100
O	11.54455200	-1.33545800	-2.03172400
C	9.38821100	-2.26356000	0.36397400
C	9.60769600	-2.21205100	2.87631400
O	9.88276400	0.01216600	3.88614600
O	7.83778000	-0.51749700	3.07240000
C	9.14092900	-2.96840900	-0.82269200
O	9.81339700	-3.20920000	-3.14907200
O	7.98001100	-1.99636400	-2.60592200
C	8.68492000	-0.01167100	-3.75345700
C	12.00745100	-2.23093700	-3.04961300
C	9.21269100	-2.92930600	1.59160900
O	11.00707200	-2.36595700	2.97281500
O	9.05571800	-2.83014200	4.03576400
C	11.29832500	-0.17930500	3.92491600
C	7.38394100	-1.10629300	4.29724000
C	8.58798400	-4.24661600	-0.77665700
C	11.20080400	-3.51181700	-2.98976600
C	7.92329500	-1.31540000	-3.86524200
H	8.18201900	0.65392700	-3.03911300
H	8.76032600	0.49825100	-4.71909000
H	11.91762900	-1.75983600	-4.03480300
H	13.06340700	-2.40980300	-2.82659400
C	8.70846200	-4.22210100	1.62157200
C	11.60436600	-1.65636400	4.06464000
C	7.66247900	-2.59387300	4.25885500
H	11.76074500	0.22230700	3.01237900
H	11.65730300	0.39320900	4.78597200
H	7.88223200	-0.63311900	5.15009400
H	6.30975400	-0.90444100	4.34323200
H	8.30668100	-4.70956500	-1.71578200
C	8.29900600	-4.87669200	0.44308300
H	11.36976200	-4.02537800	-2.03289100
H	11.45779200	-4.19221200	-3.80777900
H	8.33966600	-1.94944400	-4.65530700
H	6.86118300	-1.13965200	-4.05950900
H	8.59277200	-4.70553300	2.58473700
H	11.22640500	-2.04399300	5.01726900
H	12.67796300	-1.85340900	3.99177400
H	7.06368500	-3.06351500	3.46688200
H	7.42730200	-3.07589800	5.21303400
C	7.40862300	-6.06434700	0.48484500
C	6.55167900	-6.24613700	1.58056600
C	7.23765900	-6.92419100	-0.61765500
H	6.63828900	-5.61546300	2.45733300
C	5.50160800	-7.15781200	1.54494600
H	7.92246400	-6.88222700	-1.45734900
C	6.16905100	-7.81121200	-0.68183000
C	4.53519900	-7.17567400	2.72181900
C	5.25496200	-7.90543900	0.38460500
C	5.98273700	-8.73017500	-1.88325800
O	5.23597500	-7.09202900	3.96401500
O	3.79040100	-5.98257100	2.62001400
C	3.59473400	-8.42859800	2.71639800
C	3.99005200	-8.62971900	0.22852300
O	6.71960000	-8.29157300	-3.02142700
O	6.54986600	-9.96291600	-1.49507500
C	4.46796500	-8.90938400	-2.25757000
C	5.92642400	-8.28594600	4.33796900
C	2.78618400	-5.84253700	3.63237200
O	4.29173200	-9.57923700	3.14415300
O	2.52437900	-8.24330600	3.63860900
C	3.10378900	-8.76178700	1.31351500
C	3.56525000	-9.05682900	-1.03847400
C	6.17126300	-7.16753400	-3.71553300
C	6.38028800	-11.01699500	-2.45049300
O	4.31198700	-10.01163800	-3.15083400
O	3.98272100	-7.78001600	-2.95015100
C	4.94324600	-9.43605300	4.41174000
H	6.72066900	-8.50829900	3.61159200
H	6.38329200	-8.07895600	5.31089900
H	3.25269100	-5.80478000	4.62261100
H	2.28850300	-4.89032600	3.42647200
C	1.81953700	-7.00291300	3.52838400
C	1.80288100	-9.19685400	1.10541700
C	2.25037800	-9.47547400	-1.23717100
C	4.74072700	-7.45253200	-4.12122600
H	6.20731300	-6.27219100	-3.08007000
H	6.81643900	-7.01031100	-4.58569900
C	4.90111800	-11.23172300	-2.69675100
H	6.89811600	-10.76667900	-3.38309600
H	6.84569400	-11.89922300	-2.00122200
H	4.20112000	-9.26427600	5.19936900
H	5.45091400	-10.38829600	4.59141400
H	1.27849300	-6.95514600	2.57405500
H	1.09246000	-7.00036200	4.34657300
H	1.13534400	-9.23338500	1.95756500
C	1.32088500	-9.48664900	-0.18561400
H	1.95076100	-9.74597400	-2.24386900
H	4.69092700	-8.27377500	-4.84399000
H	4.26317500	-6.56415600	-4.54500300
H	4.40606900	-11.57269400	-1.77666000
H	4.72315300	-11.96604200	-3.48860700

$1_{[8]}$ 6-31G(d)

O	-4.04631300	-11.26235100	0.88963700
O	-3.72428600	-9.46185200	-5.16141000
O	-6.86027800	-10.35076000	0.76482800

O	-6.06444500	-11.51890600	-1.01065300
O	-4.43086600	-9.04621200	1.15377600
O	-5.35463200	-7.25117200	-5.61333400
O	-4.44621900	-6.09892000	-3.88698800
O	-2.45213700	-7.58225700	-5.13971100
C	-7.05443800	-7.41899800	-3.33611600
H	-7.29361600	-6.67568100	-4.08870100
C	-7.99404000	-7.75341700	-2.35050400
C	-5.76395500	-7.94095500	-3.31462100
C	-2.13811500	-9.75671200	-0.60908600
H	-1.83706300	-10.15358100	0.35429600
C	-3.50128400	-9.69314500	-0.89144100
C	-4.75917300	-7.40204900	-4.32463600
C	-2.99647200	-8.76164900	-3.06137100
C	-4.48327100	-10.08939200	0.20416400
C	-1.64095800	-8.84460100	-2.77395400
H	-0.93854100	-8.49934100	-3.52299800
C	-3.95139800	-9.13647500	-2.09826800
C	-1.18096900	-9.27473900	-1.51508900
C	-5.94602200	-10.28983600	-0.32729400
C	-5.36865900	-8.80714500	-2.28454100
C	-3.46845100	-8.28277400	-4.42825200
C	-5.32972200	-9.22634200	2.25477900
H	-5.06978200	-10.13424600	2.80911500
H	-5.18899800	-8.35337900	2.89922900
C	-7.62661600	-8.71917800	-1.39539000
H	-8.34752600	-9.06251400	-0.66101900
C	-4.29152400	-9.23605800	-6.45744200
H	-3.58731800	-8.67781800	-7.08429600
H	-4.45198200	-10.22972200	-6.88609000
C	-5.59436200	-8.47824000	-6.30454700
H	-6.32719100	-9.08632500	-5.75560300
H	-6.01926000	-8.20058000	-7.27430600
C	-6.33362800	-9.22885400	-1.34992200
C	-4.18511900	-12.47294300	0.14294700
H	-3.54475500	-12.44329100	-0.74970700
H	-3.83874600	-13.27530800	0.80183300
C	-6.74616300	-9.29778900	1.72428200
H	-7.02233300	-8.33644400	1.26905900
H	-7.46525700	-9.53431700	2.51495600
C	-5.63515600	-12.65768500	-0.25450700
H	-6.26905200	-12.78266800	0.63039500
H	-5.76380800	-13.51741700	-0.91861400
C	-3.47214500	-5.43642700	-4.70302000
H	-3.85604800	-5.31283100	-5.72109200
H	-3.32344700	-4.45300000	-4.24766000
C	-2.19323200	-6.24678300	-4.69480700
H	-1.76339700	-6.25903800	-3.68424500
H	-1.45063300	-5.84088800	-5.38891000
O	-10.15784600	-5.46981200	1.57613800
O	-10.39968600	-3.98216000	-5.00422800
O	-12.03845800	-3.19374900	1.79966800
O	-10.10048200	-2.83220400	0.68516200
O	-12.27267500	-5.78706700	0.81428300
O	-13.03670100	-3.16737600	-5.37594500
O	-13.84196400	-4.82347200	-4.04866400
O	-11.67083600	-5.79048600	-5.49951800
C	-13.20385200	-1.87987200	-2.88302900
H	-13.69339800	-1.48597200	-3.76637900
C	-13.10602200	-1.10179800	-1.71292600
C	-12.64003900	-3.14798500	-2.95803400
C	-9.68868900	-6.54473200	-0.96855900
H	-9.23999000	-6.96167400	-0.07425800
C	-10.63421800	-5.53782600	-0.82681400
C	-12.73409800	-3.96988300	-4.23780700
C	-10.86176800	-5.43188800	-3.23112700
C	-11.07775600	-5.08201900	0.55714500
C	-9.88389200	-6.41658800	-3.36083300
H	-9.60337400	-6.73559700	-4.35912700
C	-11.20185900	-4.93407900	-1.96387300
C	-9.23849300	-6.95261000	-2.23716000
C	-11.32106000	-3.53488900	0.61262000
C	-12.00348700	-3.71297600	-1.83751500
C	-11.42847800	-4.80213500	-4.49782900
C	-12.92512200	-5.41623100	2.03461400
H	-12.28561700	-5.65640200	2.89135200
H	-13.83477800	-6.02231700	2.07821800
C	-12.55205400	-1.71665100	-0.57964800
H	-12.45208200	-1.17140300	0.35115000
C	-10.76490300	-3.26329900	-6.18865000
H	-10.99476300	-3.96393700	-6.99838100
H	-9.88621000	-2.66998400	-6.45777400
C	-11.95401200	-2.37869900	-5.87873900
H	-11.66844500	-1.61213600	-5.14558900
H	-12.33729000	-1.88418900	-6.77691300
C	-12.02626700	-3.00380500	-0.62824100
C	-8.94942200	-4.70669200	1.63867100
H	-8.34578100	-4.87386600	0.73640000
H	-8.40169500	-5.08642400	2.50737200
C	-13.24347500	-3.93564700	1.99888300
H	-13.96030400	-3.71815100	1.19478000
H	-13.65976800	-3.58669800	2.94914800
C	-9.26835100	-3.23337000	1.77965200
H	-9.77131200	-3.02813300	2.73073400
H	-8.36442900	-2.62174800	1.70496900
C	-14.04283400	-5.77030400	-5.10490500
H	-14.26535900	-5.24755300	-6.04178000
H	-14.91025800	-6.36591700	-4.80553400
C	-12.80156900	-6.62690100	-5.24763500
H	-12.63972100	-7.21971100	-4.33642700
H	-12.87245900	-7.30500000	-6.10393600
C	-5.89814000	12.27577400	-0.11770900
C	-5.21940200	11.40732800	-2.25106700
H	-5.70406600	12.84580100	0.78435500
C	-7.16437000	11.72755200	-0.29381200
C	-6.49138400	10.88361500	-2.45418400

H	-4.46997500	11.21956600	-3.01091700
C	-8.26556200	11.96568700	0.73259800
C	-7.46716400	10.98745300	-1.45204100
C	-6.72682200	10.04870800	-3.70550500
O	-7.74638000	12.35588100	2.00110800
O	-8.99009600	13.07369500	0.24323300
C	-9.20268100	10.71675300	0.89611100
C	-8.71550000	10.22536100	-1.55376700
O	-6.07177800	8.82233400	-3.46873900
O	-6.07939400	10.62394800	-4.84138000
C	-8.24665800	9.82601700	-4.00850500
C	-7.15375000	11.30538100	2.77037700
C	-10.15065400	13.40534800	1.01500900
O	-10.38814100	11.08525100	1.60117800
O	-8.58194800	9.71966800	1.67602800
C	-9.53600700	10.04304300	-0.43002600
C	-9.04542200	9.54808600	-2.74177200
C	-6.22300000	7.87246500	-4.52960500
C	-6.70649500	11.80413400	-5.34764300
O	-8.40548200	8.79901900	-4.98616500
O	-8.82463000	10.98900500	-4.56022400
H	-6.25173100	10.92878600	2.26895600
H	-6.86572800	11.75925400	3.72388400
C	-8.14623400	10.17806100	2.96163800
H	-9.85673400	13.70374800	2.02745200
H	-10.61112200	14.25597900	0.50393200
C	-11.07987300	12.20962100	1.05427900
C	-10.58482900	9.12558100	-0.46246500
C	-10.10463000	8.65214000	-2.76592400
H	-5.77348700	8.26010500	-5.45016300
H	-5.68146400	6.97843100	-4.20694300
C	-7.69681600	7.58482900	-4.72297200
C	-8.14356100	11.49568200	-5.71419300
H	-6.66763400	12.60593600	-4.59708200
H	-6.12060800	12.10705100	-6.22121000
H	-9.00587000	10.50620100	3.55564800
H	-7.67973800	9.31301000	3.44184800
H	-11.44475900	11.97624100	0.04414500
H	-11.93864600	12.38413900	1.71014900
C	-10.85793400	8.36748600	-1.61180500
H	-11.16434100	8.98450500	0.44326800
H	-10.29776500	8.12050300	-3.68959100
H	-8.10229200	7.09314100	-3.82818000
H	-7.87256400	6.94240000	-5.59208300
H	-8.18968000	10.76521000	-6.52964100
H	-8.68751500	12.39942700	-6.00412200
C	-11.75738300	7.18585900	-1.58942600
C	-12.29237200	6.63840300	-2.77211100
C	-11.95241300	6.46736500	-0.39995500
H	-12.24668800	7.19239100	-3.70290000
C	-12.84844300	5.36585200	-2.79166500
C	-12.55480500	5.21150000	-0.39894400
H	-11.56440100	6.84279800	0.54012400
C	-13.39812200	4.77046200	-4.08173600
C	-12.94554800	4.61416100	-1.60596400
C	-12.61263900	4.44162700	0.91452500
O	-12.91476700	5.44553600	-5.24086700
O	-14.78841000	5.00632800	-4.03766400
C	-13.09811700	3.23366600	-4.18949200
C	-13.28773500	3.18983200	-1.64931900
O	-11.28986000	4.03147600	1.17477700
O	-12.97780800	5.29929600	1.99623300
C	-13.58192700	3.20924500	0.85670300
C	-11.56414900	5.15472900	-5.60661000
C	-15.52064700	4.41832200	-5.11974000
O	-13.89253100	2.65166000	-5.22375400
O	-11.75655600	3.00540200	-4.56074500
C	-13.29441000	2.49900800	-2.86960000
C	-13.45856800	2.45548600	-0.46137100
C	-11.15357700	3.25112200	2.36875800
C	-14.35091800	5.69338100	2.00436200
O	-13.36899100	2.36626800	1.98584300
O	-14.92827900	3.62720700	0.92199500
H	-10.87269900	5.53077700	-4.83972900
H	-11.38481200	5.69569500	-6.54125700
C	-11.37747000	3.66153300	-5.77628900
H	-15.20565400	4.86050100	-6.07145200
H	-16.56999400	4.66503700	-4.93349000
C	-15.29227900	2.92071100	-5.11948400
C	-13.32216400	1.10646200	-2.88801300
C	-13.54017400	1.07024500	-0.49832800
H	-11.44660200	3.84424000	3.24140300
H	-10.09140200	2.99934800	2.43856500
C	-12.00743500	2.00723200	2.24105700
C	-15.23134600	4.46115300	2.04682300
H	-14.57859400	6.29453000	1.11281800
H	-14.48116300	6.31631600	2.89491300
H	-11.97496100	3.28225600	-6.61208600
H	-10.32529700	3.40704100	-5.93526400
H	-15.69711300	2.47245100	-4.20140500
H	-15.75788600	2.43873500	-5.98489200
C	-13.38692300	0.35678200	-1.70339900
H	-13.21671000	0.61456100	-3.84804500
H	-13.68404400	0.54365400	0.43779600
H	-11.62261800	1.37235900	1.43148400
H	-12.02136100	1.42708900	3.16916200
H	-15.07930200	3.90560300	2.97890800
H	-16.28960300	4.71931100	1.94542900
C	-4.87725200	12.07428900	-1.06541500
C	4.54525100	10.13335300	0.60289900
C	4.74835000	9.49235400	1.83551000
C	5.42526000	9.79850400	-0.44557800
C	5.70416100	8.49217600	1.99156900
H	4.11476100	9.71296900	2.68625500
C	6.34352500	8.76474300	-0.31993200
H	5.37457600	10.31747600	-1.39529300

C	5.77465100	7.76209800	3.32699200
C	6.46652000	8.06618400	0.89471000
C	7.25671000	8.38698400	-1.47965000
C	7.08524200	6.91415200	3.48871300
O	5.64488900	8.67385700	4.41860800
O	4.63096300	6.93894600	3.37376900
C	7.24869500	6.83080700	0.97221000
C	7.48202300	6.83587700	-1.56349800
O	6.77803400	8.87909800	-2.72858500
O	8.47381400	9.05577400	-1.22999400
C	7.44627100	6.18017400	2.20324900
O	6.96152000	6.03102300	4.60047100
O	8.18914900	7.74825500	3.76556800
C	6.79257600	9.49442800	4.64649800
C	4.54900000	6.13275500	4.55563500
C	7.68686900	6.19360100	-0.19713500
O	6.36027600	6.19169500	-2.12474500
O	8.57510000	6.54770500	-2.43623500
C	5.66153400	8.17513100	-3.28065700
C	9.51833100	8.74287900	-2.15945000
C	7.98581200	4.90183400	2.24362300
C	5.76968100	5.23975500	4.62867800
C	8.00284700	8.61625300	4.89010500
H	6.96553200	10.15262600	3.78357000
H	6.55531500	10.10988000	5.51999400
H	4.47420000	6.77144100	5.44224600
H	3.63238800	5.54500800	4.45031700
C	8.17478100	4.89061200	-0.14635100
C	5.98637700	6.70188700	-3.41057200
C	9.78088900	7.25112300	-2.12992700
H	4.77510300	8.30787100	-2.64582500
H	5.47076400	8.63573500	-4.25511800
H	9.23591500	9.06906800	-3.16658700
H	10.39094000	9.31110300	-1.82413800
H	8.12830700	4.44797900	3.21662200
C	8.28516900	4.18882800	1.06520600
H	5.76404100	4.52895400	3.79142400
H	5.80623300	4.67916400	5.56809700
H	7.87799100	8.02568100	5.80449000
H	8.92050100	9.20754800	4.96109100
H	8.40684100	4.41199600	-1.08997100
H	6.79592100	6.53866600	-4.12998800
H	5.10675400	6.12629000	-3.71341800
H	10.15586900	6.94870400	-1.14215700
H	10.50723300	6.95155200	-2.89196900
C	8.52026800	2.72003200	1.08167400
C	8.88847000	2.01279200	-0.08054900
C	8.20993300	1.95495200	2.21763800
C	8.79132600	0.62922200	-0.15145000
H	9.21705900	2.53960900	-0.96819000
C	8.15893100	0.56487200	2.17518700
H	7.93522500	2.43036300	3.15111200
C	9.15774600	-0.11303500	-1.43077300
C	8.38143000	-0.11497800	0.96947200
C	7.68883300	-0.17526900	3.42094900
C	8.20989100	-1.33754900	-1.68795600
O	9.16910600	0.74327100	-2.56981700
O	10.48911000	-0.54195100	-1.24389700
C	8.04331100	-1.53502500	0.84582500
C	7.99051000	-1.71352400	3.36389900
O	8.25792400	0.39135900	4.60191100
O	6.30211900	0.06598400	3.50810400
C	7.89773200	-2.12204300	-0.41959600
O	8.76927800	-2.18570500	-2.69189800
O	6.96280700	-0.91424300	-2.19094000
C	7.88235200	1.12068100	-3.06899200
C	10.99604300	-1.36460100	-2.30161000
C	7.71233800	-2.29447800	1.98331400
O	9.35806000	-1.96405300	3.60467800
O	7.27206200	-2.39126600	4.39163500
C	9.64625900	0.10753700	4.78765700
C	5.67019200	-0.59746900	4.60974000
C	7.30988400	-3.37886100	-0.54675100
C	10.11716500	-2.59035400	-2.44358800
C	7.05994800	-0.11329300	-3.37512300
H	7.35790400	1.74780100	-2.33551000
H	8.07387800	1.71390300	-3.96872300
H	11.02706000	-0.79674500	-3.23813700
H	12.01422900	-1.63244000	-2.00439600
C	7.17162400	-3.56539300	1.84410400
C	9.85604200	-1.39243900	4.82040000
C	5.87556200	-2.09079600	4.46739000
H	10.23716800	0.55848500	3.97820400
H	9.92645600	0.57760800	5.73557000
H	6.08203200	-0.23132200	5.55626300
H	4.61029800	-0.33416500	4.54602800
H	7.11992800	-3.75113400	-1.54725800
C	6.88354700	-4.10464700	0.57563600
H	10.16755100	-3.20381000	-1.53310900
H	10.41149900	-3.20362800	-3.30114500
H	7.50946700	-0.69502400	-4.18698800
H	6.03166200	0.14960500	-3.64003100
H	6.93900600	-4.12575700	2.74237800
H	9.34704000	-1.83846100	5.68207800
H	10.91899500	-1.64900300	4.85270300
H	5.35785300	-2.45410500	3.56932100
H	5.49925700	-2.63712500	5.33803600
C	6.00083900	-5.28782000	0.42121200
C	5.01852700	-5.54836500	1.38831000
C	5.98894200	-6.07933000	-0.74287100
H	4.97853300	-4.96648800	2.30158400
C	4.01361600	-6.48593600	1.17556500
H	6.76799700	-5.96867800	-1.48914200
C	4.96857900	-6.99293900	-0.98403600
C	2.91151000	-6.60127100	2.22019900
C	3.94091800	-7.18030900	-0.04086400

C	4.96994400	-7.84831100	-2.24536600
O	3.44462500	-6.52402500	3.54326900
O	2.11305400	-5.45023000	2.05850600
C	2.05836700	-7.90446000	2.05934400
C	2.74872800	-7.96613800	-0.37329000
O	5.79699900	-7.30089900	-3.26892800
O	5.56806200	-9.06812200	-1.86326100
C	3.51993800	-8.08956700	-2.79655100
C	4.15549600	-7.68941000	3.96627500
C	0.98227900	-5.40513800	2.93740000
O	2.76730400	-9.02877200	2.53457700
O	0.87462700	-7.81561000	2.84778300
C	1.76046300	-8.21174900	0.59743500
C	2.50222400	-8.36467900	-1.69589700
C	5.25852900	-6.16532800	-3.95174700
C	5.57236600	-10.06829300	-2.88924800
O	3.53308200	-9.14016200	-3.76242100
O	3.04179700	-6.94773900	-3.47336600
C	3.24540600	-8.89727700	3.87850600
H	5.04741700	-7.83721200	3.34141000
H	4.47204900	-7.49340700	4.99562200
H	1.31701100	-5.37349900	3.97979400
H	0.45536500	-4.47759300	2.69492300
C	0.11148700	-6.61688300	2.68093400
C	0.53405600	-8.74625000	0.22869100
C	1.25971500	-8.88334500	-2.05652800
C	3.90047300	-6.50213100	-4.53050600
H	5.17113700	-5.31323700	-3.26384400
H	5.98248800	-5.91600600	-4.73397200
C	4.14616700	-10.35084800	-3.31463400
H	6.17347000	-9.73106800	-3.74095800
H	6.03995900	-10.94923400	-2.43972500
H	2.40145100	-8.80060200	4.57052800
H	3.78615200	-9.82419800	4.09107000
H	-0.30725600	-6.57006300	1.66680100
H	-0.71119500	-6.68509300	3.39967300
H	-0.21766500	-8.87534100	0.99791700
C	0.22846900	-9.02953800	-1.11578600
H	1.09754600	-9.13595800	-3.09879700
H	3.98107900	-7.27593800	-5.30127500
H	3.41663500	-5.61614700	-4.95210000
H	3.57556900	-10.77801700	-2.47809400
H	4.10427200	-11.04191600	-4.16234200
O	2.11710300	12.19905100	-3.48331200
O	0.60931200	13.83446200	2.43836800
O	-0.77869900	12.48619600	-4.02636900
O	0.19677600	14.06467900	-2.71876000
O	0.78414200	10.44912300	-2.94621700
O	-1.89274300	12.82052500	3.12255900
O	-1.33788700	10.72374600	2.46970300
O	0.88070500	11.93049600	3.64410100
C	-2.94387600	12.30495700	0.51731500
H	-3.61224600	12.16586800	1.35985300
C	-3.45148000	12.37887900	-0.78827300
C	-1.57237100	12.30704000	0.76453500
C	2.84766700	11.17327200	-0.92546000
H	3.40876400	10.83848900	-1.78945600
C	1.58900700	11.72073000	-1.15514400
C	-1.09468800	12.08464400	2.19421600
C	1.27460000	11.95458900	1.22577600
C	1.08345800	11.78047700	-2.59083000
C	2.55338100	11.45541100	1.43792600
H	2.91524800	11.40726100	2.45817400
C	0.75597300	12.05515400	-0.07805600
C	3.34217400	10.97558800	0.37346000
C	-0.17385600	12.70364400	-2.75402200
C	-0.66219300	12.34820100	-0.30185300
C	0.42210900	12.43649800	2.39322700
C	0.24562700	10.31259700	-4.26695200
H	0.97874300	10.64650700	-5.00901100
H	0.05219400	9.24404800	-4.39929100
C	-2.52810000	12.56874200	-1.83404200
H	-2.87605900	12.71651000	-2.84999500
C	-0.20077000	14.50528600	3.41112000
H	0.06404800	14.16708400	4.41908200
H	0.03665300	15.56835700	3.30968200
C	-1.66132900	14.23066000	3.11720600
H	-1.94194000	14.65762000	2.14414100
H	-2.31592800	14.64730700	3.88925700
C	-1.15957200	12.52554700	-1.60630200
C	2.45169800	13.58572100	-3.39619400
H	2.86319800	13.81499900	-2.40329000
H	3.22680700	13.75744300	-4.14970000
C	-1.03224800	11.11903800	-4.36212500
H	-1.79077000	10.69534900	-3.69007100
H	-1.42994000	11.13069200	-5.38184900
C	1.21617700	14.42167700	-3.66001400
H	0.85389700	14.26956100	-4.68282500
H	1.40846300	15.48610000	-3.49643100
C	-0.90742600	10.31284000	3.77317100
H	-1.46160300	10.86093200	4.54260900
H	-1.14489200	9.24720200	3.84123100
C	0.58207200	10.55442700	3.89827900
H	1.12592400	9.91000600	3.19444700
H	0.94194000	10.35356200	4.91231100

S11. References

[1] U. Mueller, R. Förster, M. Hellmig, F. U. Huschmann, A. Kastner, P. Malecki, S. Pühringer, M. Röwer, K. Sparta, M. Steffien, M. Ühlein, P. Wilk, M. S. Weiss, *Eur. Phys. J. Plus* **2015**, *130*, 141.

[2] W. Kabsch, *Acta Crystallogr. Sect. D* **2010**, *66*, 125–132.

[3] G. M. Sheldrick, *Acta Crystallogr. Sect. A* **2015**, *71*, 3–8.

[4] G. M. Sheldrick, *Acta Crystallogr. Sect. C* **2015**, *71*, 3–8.

[5] O. V. Dolomanov, L. J. Bourhis, R. J. Gildea, J. A. K. Howard, H. Puschmann, *J. Appl. Crystallogr.* **2009**, *42*, 339–341.

[6] B. Rees, L. Jenner, M. Yusupov, *Acta Crystallogr. Sect. D Biol. Crystallogr.* **2005**, *61*, 1299–1301.

[7] J. Hu, D. Zhang, F. W. Harris, *J. Org. Chem.* **2005**, *70*, 707–708.

[8] J. Merz, M. Dietz, Y. Vonhausen, F. Wöber, A. Friedrich, D. Sieh, I. Krummenacher, H. Braunschweig, M. Moos, M. Holzapfel, C. Lambert, T. B. Marder, *Chem. Eur. J.* **2020**, *26*, 438–453.

[9] M. O. Banikhaled, J. D. Mottishaw, H. Sun, *Cryst. Growth Des.* **2015**, *15*, 2235–2242.

[10] D. N. Coventry, A. S. Batsanov, A. E. Goeta, J. A. K. Howard, T. B. Marder, R. N. Perutz, *Chem. Commun.* **2005**, 2172–2174.

[11] S. Hitosugi, W. Nakanishi, T. Yamasaki, H. Isobe, *Nat. Commun.* **2011**, *2*, 492–495.

[12] L. J. Barbour, *J. Appl. Crystallogr.* **2020**, *53*, 1141–1146.

[13] D. P. van Heerden, L. J. Barbour, *Chem. Soc. Rev.* **2021**, *50*, 735–749.

[14] S. Mecozzi, J. Rebek, *Chem. Eur. J.* **1998**, *4*, 1016–1022.

[15] J. R. Long, R. S. Drago, *J. Chem. Educ.* **1982**, *59*, 1037.

[16] P. Thordarson, *Chem. Soc. Rev.* **2011**, *40*, 1305–1323.

[17] S. Miertuš, E. Scrocco, J. Tomasi, *Chem. Phys.* **1981**, *55*, 117–129.

[18] S. E. Wheeler, K. N. Houk, P. V. R. Schleyer, W. D. Allen, *J. Am. Chem. Soc.* **2009**, *131*, 2547–2560.

[19] P. Müller, *Pure Appl. Chem.* **1994**, *66*, 1077–1184.

[20] C. E. Colwell, T. W. Price, T. Stauch, R. Jasti, *Chem. Sci.* **2020**, *11*, 3923–3930.

S12. Author Contributions

N.G. and O.D. devised the project. N.G. carried out most of the synthesis and characterization. K.N. investigated the Ir-catalyzed borylations reaction to obtain compounds **2**, **3**, and **4**. N.G. carried out the

supramolecular binding experiments, theoretical calculations, single-crystal growth, and X-ray crystallography (beamline and in-house diffractometer). S.W. carried out the mass spectrometry (MALDI-TOF-MS). N.G. and O.D. wrote the manuscript, with input from the other authors.
